# Synthesis and surface spectroscopy of α-pinene isotopologues and their corresponding secondary organic material†
†Electronic supplementary information (ESI) available: Detailed information for the synthesis of isotopologues, SOM collection, and supplementary SFG data. See DOI: 10.1039/c9sc02399b


**DOI:** 10.1039/c9sc02399b

**Published:** 2019-07-31

**Authors:** Mary Alice Upshur, Marvin M. Vega, Ariana Gray Bé, Hilary M. Chase, Yue Zhang, Aashish Tuladhar, Zizwe A. Chase, Li Fu, Carlena J. Ebben, Zheming Wang, Scot T. Martin, Franz M. Geiger, Regan J. Thomson

**Affiliations:** a Department of Chemistry , Northwestern University , Evanston , IL 60208 , USA . Email: r-thomson@northwestern.edu ; Email: f-geiger@northwestern.edu; b John A. Paulson School of Engineering and Applied Sciences , Harvard University , Cambridge , MA 02138 , USA; c William R. Wiley Environmental Molecular Sciences Laboratory , Pacific Northwest National Laboratory , Richland , WA 99352 , USA; d Department of Earth and Planetary Sciences , Harvard University , Cambridge , MA 02138 , USA

## Abstract

Atmospheric aerosol–cloud interactions remain among the least understood processes within the climate system, leaving large uncertainties in the prediction of future climates. In particular, the nature of the surfaces of aerosol particles formed from biogenic terpenes, such as α-pinene, is poorly understood despite the importance of surface phenomena in their formation, growth, radiative properties, and ultimate fate. Herein we report the coupling of a site-specific deuterium labeling strategy with vibrational sum frequency generation (SFG) spectroscopy to probe the surface C–H oscillators in α-pinene-derived secondary organic aerosol material (SOM) generated in an atmospheric flow tube reactor. Three α-pinene isotopologues with methylene bridge, bridgehead methine, allylic, and vinyl deuteration were synthesized and their vapor phase SFG spectra were compared to that of unlabeled α-pinene. Subsequent analysis of the SFG spectra of their corresponding SOM revealed that deuteration of the bridge methylene C–H oscillators present on the cyclobutane ring in α-pinene leads to a considerable signal intensity decrease (*ca.* 30–40%), meriting speculation that the cyclobutane moiety remains largely intact within the surface bound species present in the SOM formed upon α-pinene oxidation. These insights provide further clues as to the complexity of aerosol particle surfaces, and establish a framework for future investigations of the heterogeneous interactions between precursor terpenes and particle surfaces that lead to aerosol particle growth under dynamically changing conditions in the atmosphere.

## Abstract 2

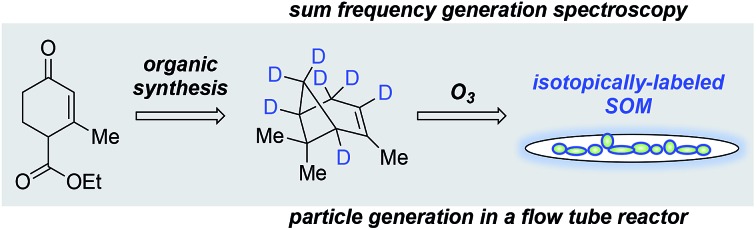
The synthesis and surface-specific spectroscopic analysis of α-pinene isotopologues and their corresponding secondary organic material is reported.

## Introduction

Acquiring mechanistic insight into the formation, growth, and climate-relevant properties of aerosol particles containing secondary organic material (SOM) from gas-phase terpenes presents an ongoing challenge in the atmospheric science and modeling communities.^1^–^4^ While the preparation and use of SOM precursor isotopologues holds the promise for enabling detailed molecular studies of aerosol particle chemistry and physics, labeling these precursors is not yet widely accessible. Here, we present the synthesis and characterization of several deuterium-labeled α-pinene isotopologues and particle-phase SOM prepared from them that enable us to begin elucidating mechanisms driving gas:particle conversion, cloud activation, and the ultimate fate of particles containing SOM.

The formation, composition, and physicochemical properties of biogenic SOM-containing particles are reasonably well known to depend on the structure and reactivity of terpene volatile organic compound (VOC) precursors, tropospheric conditions of temperature and relative humidity (RH), and the extent of particle phase state heterogeneity.^4^–^7^ In contrast, heterogeneous phenomena occurring at particle surfaces remain elusive, despite their intrinsic importance in key formation and growth processes, *i.e.* condensational growth (gas:particle conversion),^8^ coagulation (particle:particle merging),^9^ and cloud droplet activation (cloud condensation nucleation activity).^10^,^11^ Scientific insights into the surface-specific molecular origins of SOM-containing particle formation, growth, and cloud activation are therefore critical to the study of such constituents in the climate system, yet direct studies of the particle interface are notoriously challenging to carry out nondestructively with chemical specificity and under tropospherically relevant conditions of temperature, pressure, and RH.^4^,^12^,^13^

Our previous work has demonstrated the applicability of vibrational sum frequency generation (SFG) spectroscopy as a nondestructive nonlinear optical probe to analyze surface-localized oscillators of particle-phase SOM.^8^,^13^–^15^ SFG spectroscopy has revealed molecular information regarding the structure, dynamics, and orientations of terpenes and their oxidation products at atmospheric model interfaces in the C–H and C=O vibrational stretching regions.^16^–^19^ Notably, the SFG intensity obtained from α-pinene-derived SOM in the C–H region is independent of particle size^8^,^20^ and can be readily tracked under varying conditions of RH and temperature in real time.

These initial studies have unlocked the prospect of using SFG spectroscopy to probe the dynamics of VOC uptake onto or into the particle phase under varying RH, temperature, and consequently particle viscosity.^13^,^16^ Surface-selectively investigating such early-stage heterogeneous interaction events leading to particle generation would provide critical new insights useful to solving the outstanding uncertainties within aerosol particle mechanisms. Unfortunately, the SFG spectra of most terpenes and oxidation products relevant to biogenic SOM remain complex and congested, with overlapping spectral lineshapes that make vibrational mode assignments challenging.^16^–^19^ Moreover, the signal frequencies and intensities obtained from SOM derived from a particular terpene overlap significantly with those of the precursor molecule, making the aforementioned dynamic studies nearly impossible.^8^,^13^ For instance, the highest SFG signal intensity obtained from α-pinene-derived SOM collected on a filter sample occurs at 2950 cm^–1^, shifted only by ∼15 cm^–1^ from the otherwise similarly shaped SFG spectrum of α-pinene itself.^8^,^21^–^24^ Site-specific deuterium labeling offers the opportunity to address these limitations by “silencing” signal intensity generating C–H oscillators in the SFG spectra of both the precursor terpene and its corresponding SOM, thereby enabling vibrational mode elucidation of the highly congested SFG spectra. We also envision probing the interactions between unlabeled VOCs and labeled particle surfaces using deuterated precursors and preparing labeled SOM from them to distinguish the unlabeled gas-phase molecules from those in the particle phase.

With such goals in mind, we present here results from a detailed isotope-labeling study in which we synthesized novel α-pinene isotopologues and their corresponding SOM, and characterized them using SFG spectroscopy. We expand upon our previous work on methyl C–D α-pinene isotopologues^22^ by presenting the synthesis and SFG spectroscopy of three new analogues with methylene bridge, bridgehead methine, allylic, and vinyl deuteration. Specifically, we report on the development of a strategy for the *de novo* synthesis of α-pinene isotopologues adapted from a previous total synthesis completed by Thomas and Fallis.^25^,^26^ We then provide SFG spectra of the isotopologues at vapor:solid interfaces to investigate contributions from such C–H stretches on the complex SFG spectrum of α-pinene in the pursuit of identifying high signal intensity generating oscillators. Spectra of the ring-substituted isotopologues revealed up to 80% suppression of the dominant SFG signal intensity (around 2930 cm^–1^) observed in the unlabeled α-pinene spectrum. Following the preparation of SOM from all accessible isotopologues, the ring-substituted SOM SFG spectra reveal a considerable, albeit far from complete reduction of the 2950 cm^–1^ signal intensity, indicating that individual oscillators on the SOM surface can be chemically accessed and thus identified. These first attempts toward identifying the surface oscillators in α-pinene-derived SOM will enable future investigations of adsorption, desorption, and reactive uptake of the gas-phase species onto or into aerosol particles, with direct implications for elucidating aerosol formation and growth mechanisms. Beyond such surface spectroscopic studies, our general synthetic route offers the opportunity to utilize site-specific isotopically labeled α-pinene analogues in new mass spectroscopy-coupled chamber studies on SOM formation, growth, and properties within the greater atmospheric community.

## Results and discussion

### Synthesis of α-pinene isotopologues

**Fig. 1:**
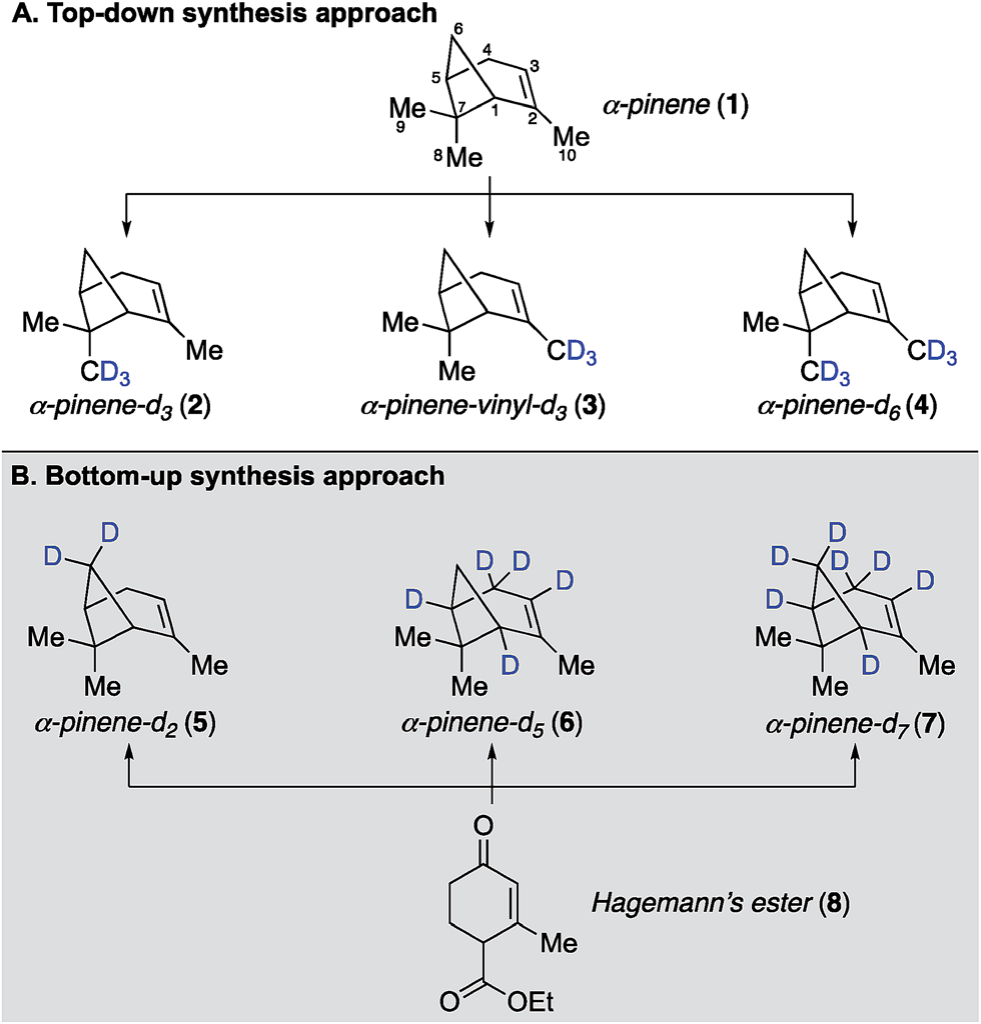
α-Pinene isotopologues synthesized in this study. Atom numbering throughout this manuscript follows the convention indicated by compound **1**.

Our earlier site-specific deuterium labeling of C8 and C10 methyl C–H oscillators in α-pinene utilized naturally occurring α-pinene as a convenient starting material, which allowed a top-down approach to access endo-methyl and/or vinyl-methyl deuterated analogs **2–4** (Fig. 1A). This approach enabled the unambiguous spectral assignment of the vinyl C10 methyl symmetric stretch within the complex vibrational spectrum of α-pinene,^22^ but was not broadly applicable to labeling less accessible sites like the C6 methylene bridge and C1/C5 bridgehead positions. We therefore considered that a more general approach capable of labeling any position on α-pinene might involve a *de novo* or bottom-up synthesis, wherein specific steps in the synthetic sequence would enable either hydrogen or deuterium incorporation and thus allow for the generation of multiple isotopologues in a unified fashion (Fig. 1B). Within this context, the previously reported total synthesis of α-pinene and β-pinene completed by Thomas and Fallis in 1973 (ref. 25 and 26) represented an ideal starting point for our approach. Their synthesis utilized Hagemann's ester as a starting material, and our analysis of their route indicated that it should be feasible to incorporate deuterium atoms at several positions with high levels of control. The successful development of such a bottom-up approach that allows for deuteration at C1 and C3 to C6, as exemplified by the synthesis of three new analogues **5–7** with methylene bridge, bridgehead methine, allylic, and vinyl deuteration, is described below.

**Scheme 1:**
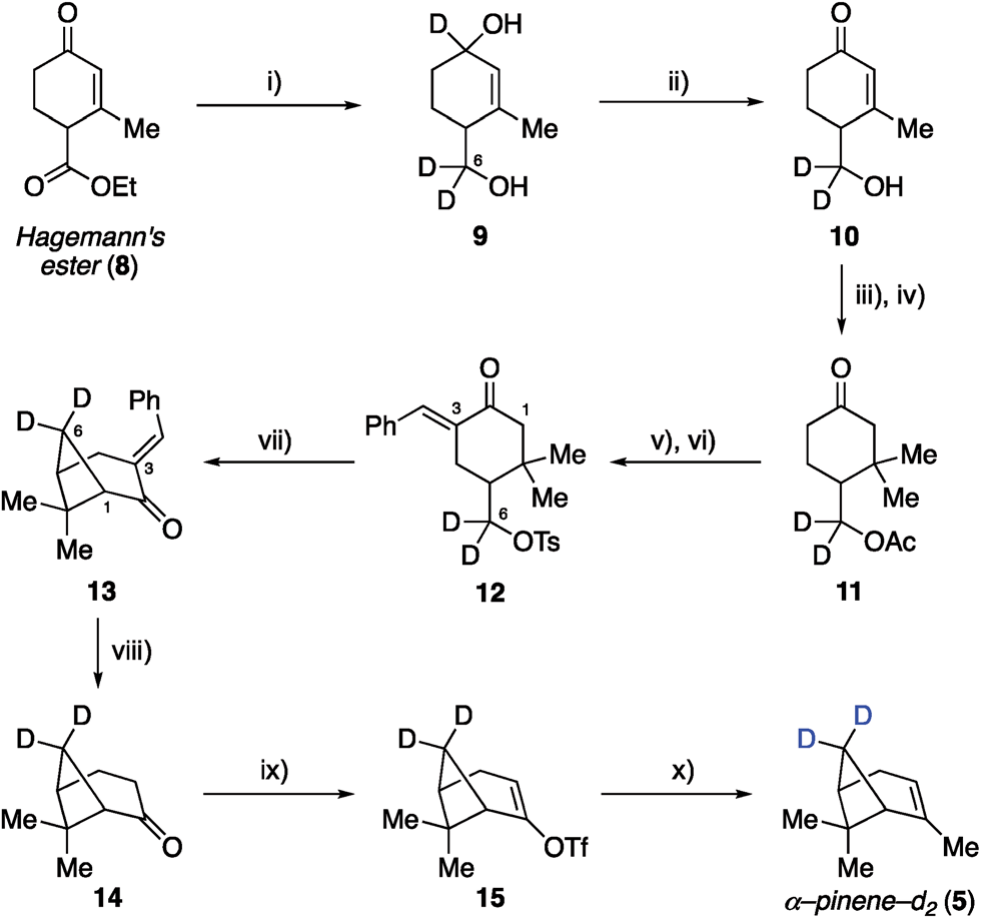
Synthesis of deuterated α-pinene-*d*_2_ (**5**). Reagents and conditions: (i) LiAlD_4_ (1.1 eq.), Et_2_O, 0 °C, 0.5 h, 72%; (ii) DDQ (1.3 eq.), 1,4-dioxane, 18 h, 78%; (iii) Ac_2_O (1.3 eq.), DMAP (5 mol%), DCM, 12 h, 89%; (iv) MeMgBr (1.5 eq.), CuBr·DMS (1 eq.), THF, –30 °C, 0.5 h, 75%; (v) benzaldehyde (2.2 eq.), 10% NaOH (1.25 eq.), EtOH, 12 h; (vi) TsCl (1.5 eq.), NEt_3_ (2 eq.), DCM, 12 h, 73% over 2 steps; (vii) NaH (2 eq.), DME, 80 °C, 1 h, 88%; (viii) 7 M KOH (10 eq.), GABA (0.25 eq.), DMSO, 100 °C, 0.75 h, 41% yield (71% brsm); (ix) LDA (1.5 eq.), Comins' reagent (2 eq.), THF, –78 to 0 °C, 2 h; (x) MeLi (2.7 eq.), CuI (2 eq.), THF, –5 °C, 14 h, 51% over 2 steps.

Synthetic efforts commenced with targeting deuteration of the C6 methylene bridge of α-pinene (Scheme 1), referred to here as α-pinene-*d*_2_ (**5**). Reduction of Hagemann's ester (**8**) with LiAlD_4_ generated diol **9** (72% yield), wherein the labeled hydroxyl methyl appendage represented incorporation of deuterium at the site that would eventually become the C6 methylene bridge within the target. Selective oxidation of the allylic hydroxyl group within **9** by DDQ provided enone **10**, which was converted to ketone **11** following acetylation and methyl cuprate 1,4-addition (52% yield over three steps from **9**). Ketone **11** was then transformed into tosylate **12** in 73% yield by first conducting a base-promoted aldol condensation with benzaldehyde followed by tosylation of the liberated primary alcohol. As demonstrated by Thomas and Fallis, installation of the benzylidene group on the less hindered C3 side of the ketone within **11** was necessary to enforce production of the desired regioisomer during the subsequent formation of the cyclobutane ring. Accordingly, exposure of tosylate **12** to sodium hydride in dimethoxyethane at 80 °C afforded methylene bridge labeled bicycloheptane **13** in 88% yield. Removal of the benzylidene blocking group was accomplished using γ-aminobutyric acid (GABA) as a retro–aldol catalyst, as described by Thomas and Fallis.^25^ Somewhat disappointingly, the best reproducible yield obtained for nopinone-*d*_2_ (**14**) under optimized conditions was 41% yield (71% yield based on recovered starting material), in part due to product volatility. With advanced material in hand, however, the synthesis of α-pinene-*d*_2_ (**5**) was completed *via* triflate **15**, which underwent methylation with methyl cuprate under the procedure we had developed previously (51% yield over two steps from **14**).^22^

**Scheme 2:**
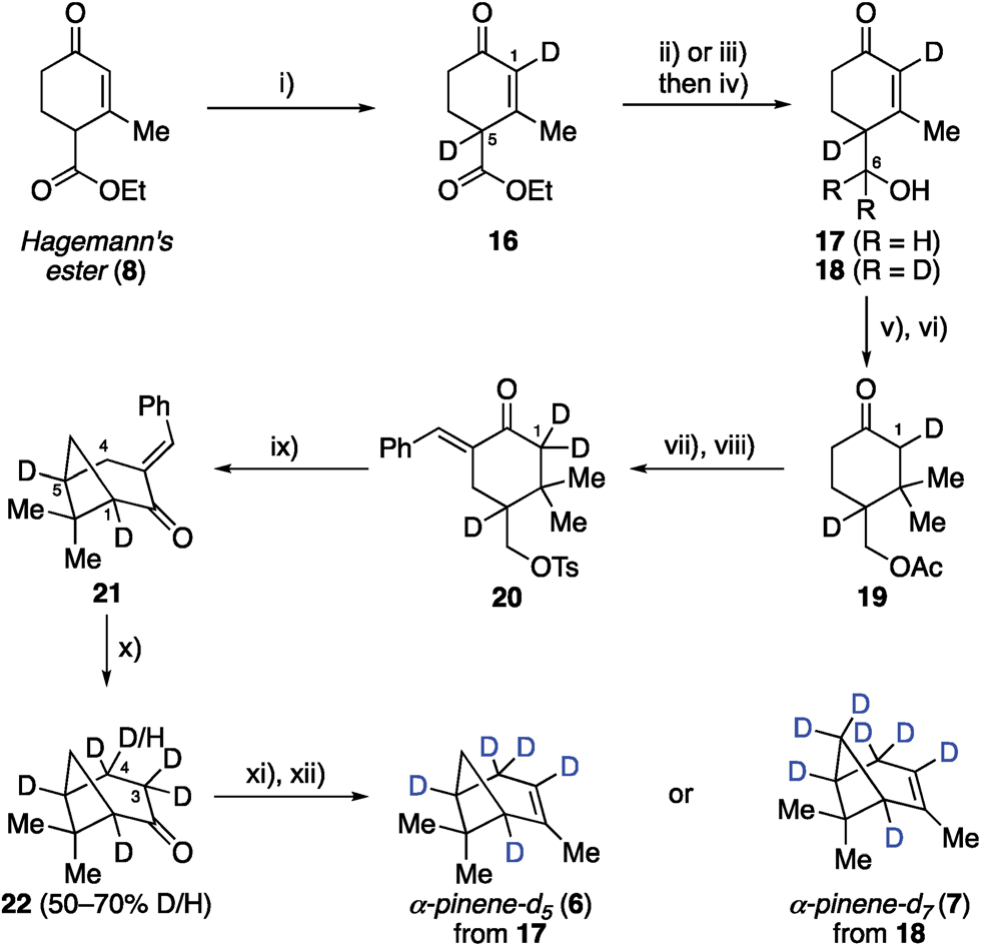
Synthesis of deuterated α-pinene-*d*_5_ (**6**) and α-pinene-*d*_7_ (**7**). Reagents and conditions: (i) NaH (4 eq.), THF, 0 °C, 1.5 h, then D_2_O, D_2_SO_4_, pH 2, 1 h, 89%; (ii) LiAlH_4_ (1.2 eq.), Et_2_O, 0 °C, 3 h, 94%; (iii) LiAlD_4_ (0.9 eq.), Et_2_O, 0 °C, 3 h, 83%; (iv) DDQ (1.2 eq.), 1,4-dioxane, 18 h, 91% of **17** or 78% of **18**; (v) Ac_2_O (1.3 eq.), DMAP (5 mol%), DCM, 12 h, 85%; (vi) MeMgBr (1.5 eq.), CuBr·DMS (1 eq.), THF, –30 °C, 0.5 h, 74%; (vii) benzaldehyde (2.2 eq.), 10% NaOD (1.25 eq.), EtOH, 12 h; (viii) TsCl (1.5 eq.), NEt_3_ (2 eq.), DCM, 12 h, 63% over 2 steps; (ix) NaH (2 eq.), DME, 80 °C, 1 h, 93%; (x) 7 M NaOD (7 eq.), *d*_6_-DMSO, 90 °C, 0.75 h, 20% yield (41% brsm); (xi) LDA (1.5 eq.), Comins' reagent (2 eq.), THF, –78 to 0 °C, 2 h; (xii) MeLi (2.7 eq.), CuI (2 eq.), THF, –5 °C, 14 h, 36% over 2 steps.

Having established the viability of modifying the Thomas and Fallis synthesis to generate methylene bridge labeled analogue, α-pinene-*d*_2_ (**5**), we turned our attention to installing deuterium at the two bridgehead positions (Scheme 2). We reasoned that deprotonation of ester **8** to yield an extended enolate might allow for direct labeling at the desired position(s) upon quenching with an appropriate source of deuterium. To that end, Hagemann's ester (**8**) was treated with excess sodium hydride to generate a solution of the requisite enolate, which was quenched by the slow addition of D_2_O and then carefully acidified with deuterated sulfuric acid. Under optimized conditions, we found that ester **16** could be generated in 88% yield with complete incorporation of two deuterium atoms at both C1 and C5. We were initially surprised to see labeling of the enone vinyl position, but this can be rationalized by considering the highly delocalized nature of the enolate formed from Hagemann's ester. It was important that the reaction mixture be kept at a pH of ∼2.0 during the quench in order to ensure complete deuteration at both positions, lest an inseparable mixture of partially labeled species form. From this point deuterated Hagemann's ester (**8**) was treated similarly to the previous modified synthesis presented. Reduction with LiAlH_4_ followed by allylic oxidation with DDQ provided enone **17** (85% over two steps). Acetylation of the free hydroxyl and conjugate addition with the cuprate reagent produced acetate **19**. When benzylidene formation was conducted under the original conditions, hydrogen–deuterium exchange had occurred adjacent to the ketone, removing the C1 deuterium label previously inserted. The exchange could be easily avoided by using 10% NaOD during the aldol condensation, allowing ketone **20** to be isolated with complete deuterium installation at C1. Subsequent treatment of ketone **20** with sodium hydride induced cyclization as before to generate cyclobutane **21** in 89% yield, which now possessed deuterium labels at both C1 and C5 bridgehead carbons.

Removing the benzylidene group within **21** proved to be problematic due to unforeseen loss of the bridgehead deuterium atoms. GABA was found to facilitate this process, as was the use of NaOH and regular DMSO. To circumvent this issue, we conducted the retro–aldol reaction in the absence of GABA using NaOD and deuterated DMSO as the solvent. Under our best conditions found, the benzilidene group could be cleaved without loss of bridgehead deuterium atoms in 20% yield (41% brsm), however, we now observed additional deuteration α- and β- to the ketone at C3 and C4, respectively (*i.e.*, **22** was the major product). While deuteration next to the ketone was not unexpected, we were surprised to see deuteration at the β-position. We reason that deprotonation of **21** at the C4 allylic position must be facilitating this exchange process before the desired retro–aldol reaction takes place. Unfortunately, despite much experimentation, the efficiency of this allylic exchange could not be rendered 100% effective; ^1^H NMR spectroscopy of **22** shows 50–70% of double to single deuteration at C4. This inefficiency, however, was not expected to be problematic in the planned SOM synthesis and spectroscopic analysis, and so we pressed forward to generate the desired α-pinene-*d*_5_ (**6**) as in the prior analogue synthesis. We also prepared α-pinene-*d*_7_ (**7**), substituting LiAlD_4_ for the reduction of deuterated Hagemann's ester (**8**) to generate alcohol **18**, which was subjected to the same sequence as described for α-pinene-*d*_5_ (**6**, see ESI† for complete details).

### SFG spectroscopy of α-pinene isotopologues

With synthetic isotopologues **5–7** in hand, we expected that deuteration of any position contributing to the SFG response of unlabeled α-pinene would change the SFG signal intensity obtained from the deuterated analogues, effectively leading to intensity “silencing” of the deuterated C–H oscillators within the SFG spectra. However, we note that SFG signal generation is coherent in nature, akin to a vector field of oscillators adding up in space to produce a final signal vector arriving at the detector (*i.e.*, a total signal at a given frequency), and thus, elimination of one or more vectors by deuteration does not necessarily lead to an overall total signal reduction. Moreover, as is the case in all vibrational spectroscopies, we caution that the observed signal could be produced not only from the coherent superposition of the electric fields generated from SFG-active oscillators at a given frequency, but also those coupled to modes oscillating at proximate frequencies.

**Fig. 2:**
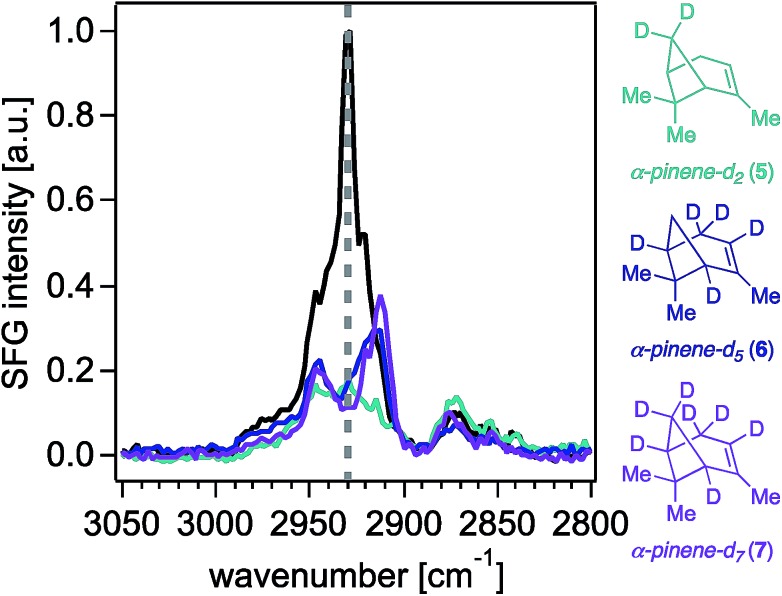
Sub 1 cm^–1^-resolution ssp-polarized vapor-phase SFG spectra of (–)-α-pinene (black trace) and isotopologues **5–7** in contact with fused silica. Intensities are scaled based the highest intensity peak of the vapor (–)-α-pinene spectrum collected on the same day as each of the isotopologues.

Fig. 2 shows the 0.6 cm^–1^ resolution SFG spectra of vapors of isotopologues **5–7** and of unlabeled α-pinene in contact with fused silica (Fig. S2† shows that spectra obtained on CaF_2_ are comparable, while Fig. S3† shows standard-resolution spectra obtained at NU). The SFG response of unlabeled α-pinene is predominantly observed within two vibrational frequency regions centered around 2930 cm^–1^ and 2880 cm^–1^. Previous atomistic simulation studies accounting for Fermi resonances and nonlocal modes have reported that the bridge methylene group on α-pinene contributes considerably to the dominant spectral feature around 2930–2940 cm^–1^.^27^ Specifically, static DFT calculations indicate that the 2930–2940 cm^–1^ peak is 77% composed of the bridgehead methylene symmetric stretch and a Fermi resonance (FR), with the remaining 23% of the SFG response at this frequency due to overtone bands of the *gem*-dimethyl CH_3_ symmetric stretches (19%), the asymmetric stretch of the vinyl CH_3_ (1%), and other stretches (3%).^27^ MD simulations indicate that the bridgehead methylene symmetric stretch and FR contribute 79%, with the remaining contributions from the overtone band FR of the *gem*-dimethyl CH_3_ symmetric stretches (12%), the *gem*-dimethyl CH_3_ asymmetric stretch (3%), vinyl CH_3_ asymmetric stretch (1%), bridging methine CHs (4%), and other modes (1%).^27^ At 2880 cm^–1^, static DFT calculations indicate that ∼75% of the SFG response is due to the *gem*-dimethyl CH_3_ symmetric stretches and their Fermi resonance, with the remainder being due to the allylic CH_2_ symmetric and asymmetric stretching modes (∼20%) and other modes.^27^ The C–H symmetric stretch on the vinyl CH_3_ group was found to contribute to the 2880 cm^–1^ peak by a somewhat greater extent (*ca.* 2%) in the ensemble-averaged spectrum from MD simulations than in the static DFT calculations.^27^ Isotopologues **5–7** each exhibit a marked decrease in the SFG response in the 2930 cm^–1^ region relative to unlabeled α-pinene, while their methyl symmetric stretching modes between ∼2850 cm^–1^ and ∼2880 cm^–1^ are largely unchanged, as expected. The latter outcome suggests that the molecular orientation distributions of the isotopologues **5–7** are comparable to that of the unlabeled compound, which is an important result given the dependence of SFG responses on molecular orientation.

**Fig. 3:**
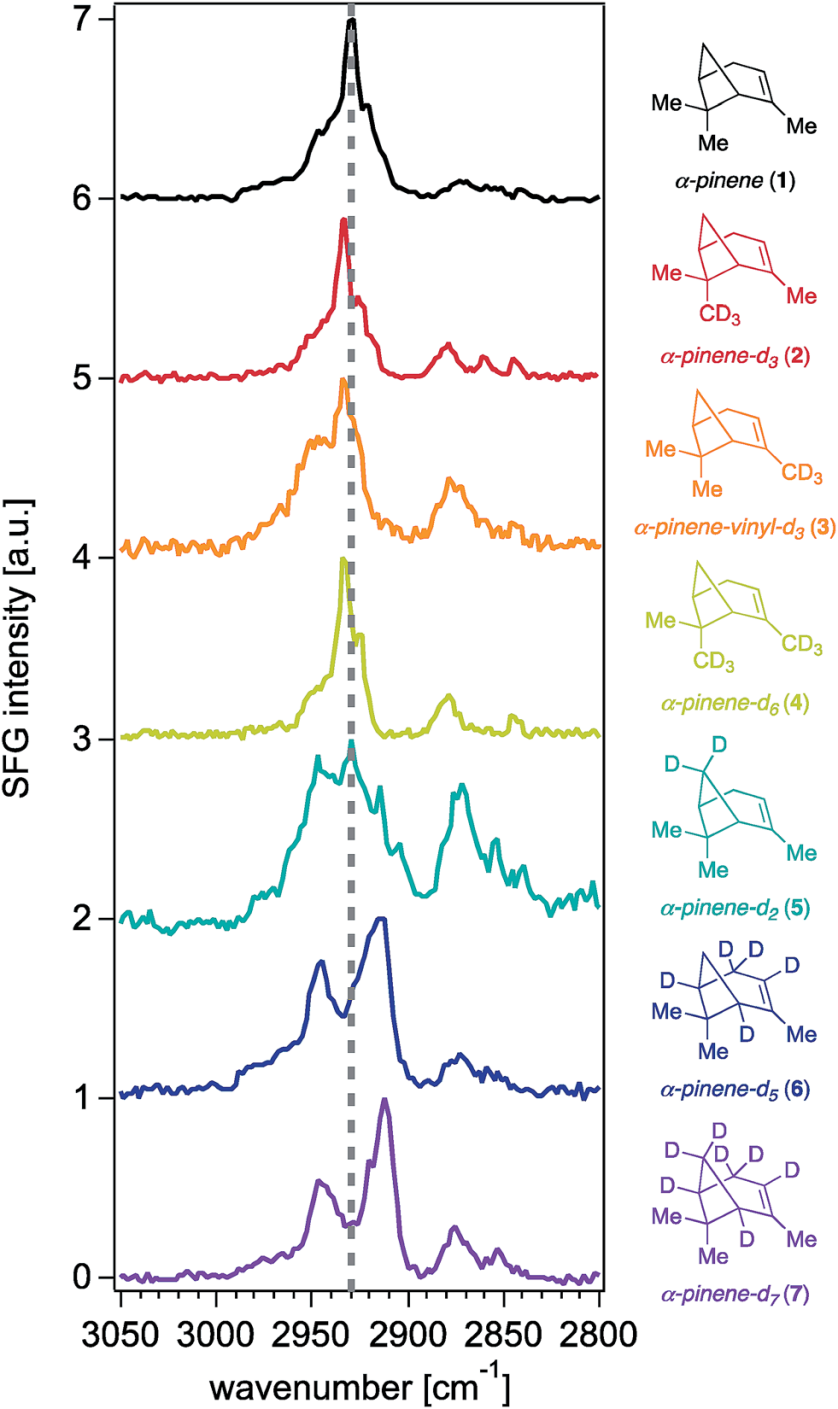
Sub 1 cm^–1^-resolution ssp-polarized vapor-phase SFG spectra of (–)-α-pinene and isotopologues **2–7** in contact with fused silica, with all maximum intensities normalized to 1 and offset for clarity.

Fig. 3 shows that contributions to the 2930 cm^–1^ signal from the methyl groups seem to be minor if not negligible, as deuterating one or two of those groups (isotopologues **2–4**) still produces the highest peak intensity at that frequency.^22^ The overall reduction of signal intensity in the 2930 cm^–1^ region exhibited in the spectra of isotopologues **5** and **7** is consistent with the previously reported contributions from deuteration of the bridge methylene group,^27^ but is rather unexpected for isotopologue **6**, given its bridge methylene group is not deuterated. The results shown in Fig. 2 and 3 then suggest that the two bridging methine groups on the ring (deuterated in isotopologues **6** and **7**) also contribute somewhat to the overall SFG response at ∼2930 cm^–1^, perhaps to a larger extent than the *ca.* 4% previously reported by MD simulations.^27^ Similar interactions between C–H oscillators in isotopologues of isoprene epoxydiol have already been described earlier.^28^ Additionally, given the aforementioned coherent nature of the SFG process, this outcome may reveal how the vector field addition that produces the observed SFG signal may be ultimately influenced by deuteration pattern. The band centered at 2840 cm^–1^ disappears completely in the spectra of isotopologues **6** and **7**, likely due to silencing of the allylic methylene group, which agrees with predictions from the discussed computations,^27^ though we cannot unambiguously assign this band given that there exists partial deuteration at that position (*ca.* 50–70% of double to single deuteration at C4). Synthetic efforts are underway to selectively deuterate the allylic and vinyl positions of α-pinene, though such studies are beyond the scope of this work. Nevertheless, we caution that the unambiguous assignment of the 2930 cm^–1^ peak will continue to be challenging.

### SFG spectroscopy of isotope-labeled α-pinene-derived SOM

Given the results presented in the previous section, isotopologues **2–7** should serve as test molecules for probing SOM formation pathways that populate the aerosol gas:particle interface with surface-active α-pinene oxidation products such as pinonaldehyde, pinonic acid, and other proposed higher order products.^29^–^31^ This organic pool has already been shown to be key for understanding droplet growth^32^,^33^ as well as cloud activation potential.^11^ The known surface-active α-pinene oxidation products contain the dimethylcyclobutane group,^11^ which we hypothesize is net-ordered at the particle surface, given its nonpolar character. Supporting this hypothesis is the observation that the SFG spectra of α-pinene-derived SOM deposited onto Teflon filters or optical windows are dominated by an intense peak at ∼2945–50 cm^–1^, with a smaller peak at ∼2880 cm^–1^. Moreover, the lineshapes are highly conserved through the particle growth process, as SFG spectra of size-resolved particles containing SOM show.^8^ In general, the SFG spectra of α-pinene-derived SOM and of α-pinene vapor in contact with optical windows are similar in lineshape but shifted by ∼15 cm^–1^.^8^,^13^,^23^,^24^ Given this information, and the results presented in the previous section, deuterating all or part of the four-membered ring should then lead to considerable reductions in SFG signal intensity from α-pinene-derived SOM near ∼2945–50 cm^–1^. We note that our previous work comparing the spectra of α-pinene-derived SOM using standard *versus* high resolution SFG systems indicates that no gain in spectral resolution is observed in the α-pinene-derived SOM spectra collected at increased spectral resolving power.^13^ Therefore, it was concluded that the Northwestern University SFG system is appropriate for analyzing the surfaces of α-pinene-derived SOM,^13^ and all SOM spectra presented herein were collected using this system.

**Fig. 4:**
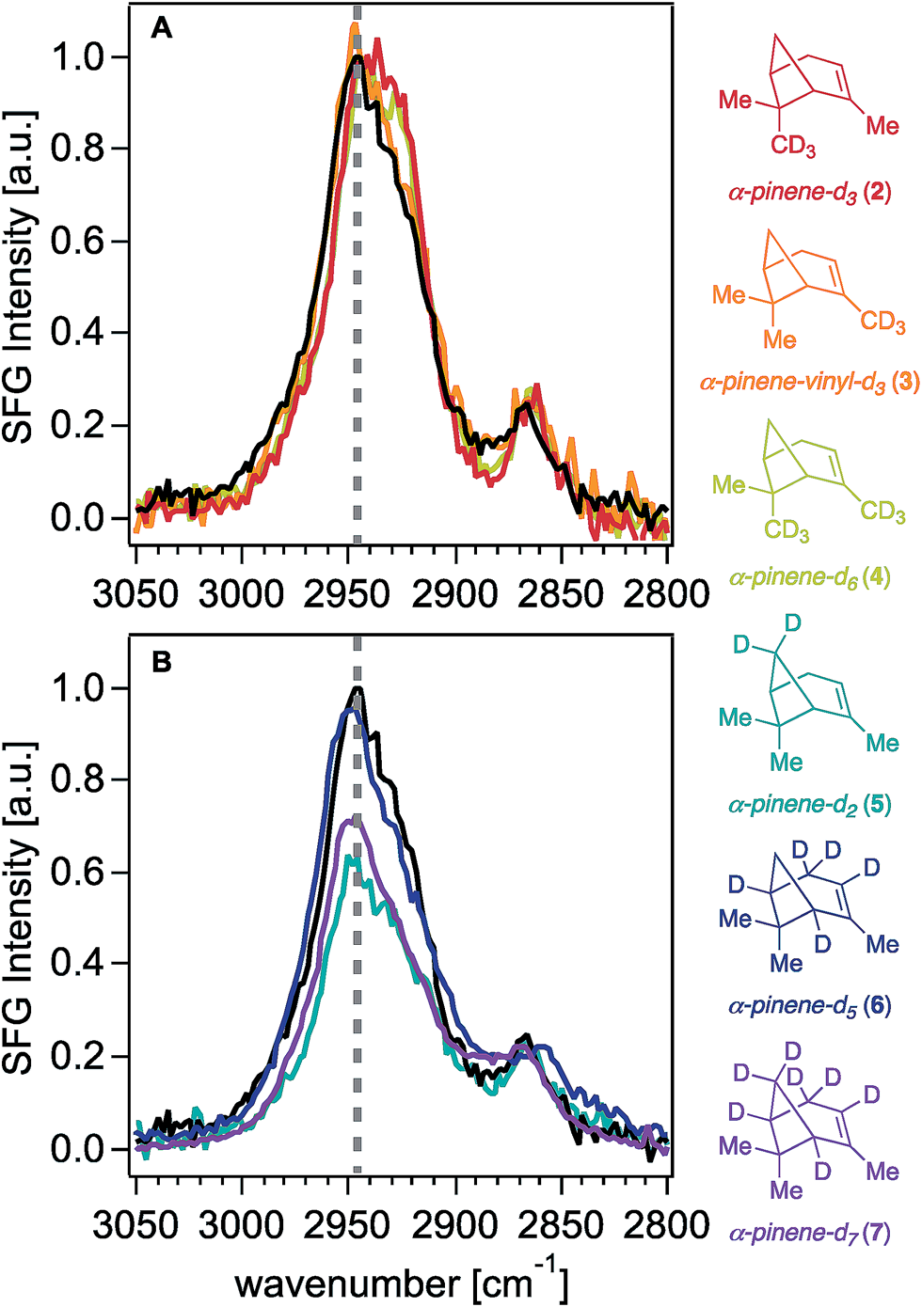
Standard resolution ssp-polarized SFG spectra of SOM produced from (–)-α-pinene (black trace) and α-pinene isotopologues (A) **2–4** and (B) **5–7** in contact with fused silica. Intensities are scaled based the highest intensity peak of the (–)-α-pinene SOM SFG spectrum collected on the same day as each of the isotopologue SOM samples.

Fig. 4 shows the standard resolution SFG spectra of SOM derived from isotopologues **2–7** compared to that of SOM derived from unlabeled α-pinene. As perhaps anticipated, the SFG spectral lineshapes for SOM derived from isotopologues **2–4** are largely invariant with methyl group deuteration (Fig. 4A). In contrast, and surprisingly to us, none of the complex lineshape changes observed for the ring-labeled isotopologues (**5–7**) are observed for the SOM prepared from them (Fig. 4B). Yet, we note a 30 to 40 percent signal intensity reduction in the 2950 cm^–1^ region for SOM prepared from isotopologues **5** and **7**, which are the only isotopologues we studied that feature a deuterated methylene group on the four-membered ring. It appears that this reduction originates from deuterating the CH_2_ group on the four-membered ring, since isotope labeling of the methine positions (isotopologue **6**) on the same ring do not result in any changes in the surface SFG spectrum of the corresponding SOM. We conclude that the high-intensity peak seen in the SFG spectra of α-pinene-derived SOM at ∼2950 cm^–1^ contains considerable contributions (*ca.* 30%) from the methylene group on the four-membered ring, while the methyl groups do not contribute.

Together, our findings support the hypothesis that the surface-active constituents of α-pinene-derived SOM conserve the cyclobutane functionality, as the bridge methylene group was found to contribute *ca.* 30% of spectral intensity in the SFG response at ∼2950 cm^–1^. While isotope labeling of these positions in isotopologues **5** and **7** resulted in significant changes in the signal intensities and lineshapes within the vapor phase SFG spectra, it appears as though the bridge methylene oscillators in the SOM do not contribute nearly as much intensity as initially expected to the ssp-polarized SFG spectra. It is possible that this outcome may arise from differences in the orientation distributions of the cyclobutane moieties present in the SOM surface-active oxidation products compared to those present in the precursor isotopologues. However, we expect it is more likely that the remaining signal intensity around 2950 cm^–1^ in the SFG spectra of SOM prepared from the bridge methylene deuterated isotopologues **5** and **7** is attributed to C–H oscillators of α-pinene and its oxidation products that have not yet been identified through deuteration.

## Conclusions

Dually motivated to synthesize α-pinene isotopologues for probing the interfacial chemistry of atmospheric organic aerosol particles by surface-specific vibrational spectroscopy, and for other MS-coupled chamber studies within the greater atmospheric community, we have successfully synthesized three different isotopologues of α-pinene by modifying a previous synthesis completed by Thomas and Fallis. These modifications allowed for deuterium labeling of the vinylogous position, both methine protons, the methylene bridgehead, and the allylic methylene positions of α-pinene. The synthesis and isolation of α-pinene isotopologues opened the possibility of using these isotopologues for vibrational mode elucidation of the high congested SFG spectrum of α-pinene and its corresponding SOM.^18^,^22^

With this goal in mind, we provided SFG spectra of the ring- and methyl-substituted isotopologues at vapor:solid interfaces to observe the relative contributions from silenced C–H oscillators on the SFG spectrum of α-pinene. The ring-substituted isotopologues revealed a significant (50–80%) suppression of the SFG signal intensity in the broad band, centered around 2930 cm^–1^, that is emblematic of unlabeled α-pinene. In contrast, the methyl-deuterated isotopologues we previously examined^22^ do not show such a signal intensity reduction, emphasizing the coherent nature of the SFG signal generation process from the various C–H oscillators in the highly strained bicyclic system of α-pinene, and the need for complementary experimental and computational studies for further spectral deconvolution. Future work will include accessing the C–D region, carrying out internal heterodyne phase-resolved measurements, improving SFG calculations, and synthesizing other α-pinene isotopologues.

By subsequently preparing and collecting SFG spectra of SOM derived from the α-pinene isotopologues, we then made the first attempts thus far toward identifying the surface oscillators in α-pinene-derived SOM. From the SOM SFG data, we speculate the presence of the dimethylcyclobutane ring at the SOM surface, as SOM prepared from the ozone oxidation of two isotopologues featuring a deuterated bridge methylene group shows up to 40% less signal intensity than SOM prepared from the methyl-substituted isotopologues to which we had access. Yet, given the substantially higher (up to 80%) SFG signal intensity reductions observed in the SOM precursor isotopologues that are bridge-deuterated, we conclude that SOM formation leads to other SFG active modes in the C–H stretching region at the particle surface.

Ultimately, our synthesis of isotopically labeled SOM and analysis of their surface-active constituents opens the door to future investigations of the dynamics of gas-phase VOC adsorption on and/or uptake into the particle phase under varying tropospherically realistic conditions.^13^,^16^ Beyond the SFG studies presented here, our syntheses of α-pinene isotopologues have been used in mechanistic chamber-based studies, including the demonstration of particle–particle chemical exchange using single-particle mass spectrometry,^34^ as well as identification of intramolecular hydrogen migration in autoxidation leading to highly oxygenated multifunctional organic compounds.^35^ More broadly speaking, synthetically accessing isotopologues of terpene hydrocarbons that are not commercially available offers the scientific community at large new opportunities to elucidate mechanistic questions in aerosol formation and growth, terpenoid biosynthesis, sustainable biofuels, and food and flavor chemistry, among other areas of study.^34^,^36^–^39^

## Methods

### Synthesis of α-pinene isotopologues

See ESI† for the synthetic procedures that were followed.

### Collection of synthetic secondary organic material (SOM)

A complete description of the Harvard flow tube reactor used in this work has been previously published.^8^,^13^,^40^ In brief, SOM was produced from (–)-α-pinene (99%, optical purity ee: 97% (GLC), Sigma Aldrich, Inc.) and the synthesized α-pinene isotopologues by introducing a solution of each precursor in 1-butanol (1 : 625 v/v dilution ratio)^8^,^41^,^42^ at selected injection rates that altered the gas phase concentration in the range of 125–300 ppb, which subsequently changed the organic particle mass loading in the flow tube. 1-Butanol was used as an OH scavenger to ensure that ozonolysis products were generated. Excess ozone (53 ppm) was introduced *via* a flow inlet arranged perpendicular to the VOC inlet to promote rapid mixing passed through the reactor (4 SLPM flow rate) and ensure the precursor was fully reacted. The particles were produced in the absence of nucleating seed particles and collected for 10 h on Teflon filters (0.2–0.45 μm pore size, Sigma-Aldrich Inc.) with a collection efficiency of 98%. From the flow rate, collection time, and particle mass concentration (obtained from a scanning mobility particle sizer), the mass of the particles collected on the filters was estimated in the range of 0.1–1.8 mg (see ESI† for more details on SOM collection). Following particle collection, samples were stored in a –20 °C freezer and sealed using Teflon tape and parafilm. Samples were warmed to room temperature prior to SFG measurements.

### Vibrational sum frequency generation spectroscopy

SFG spectra of (–)-α-pinene and the synthesized α-pinene isotopologues (**5–7**) were recorded in the C–H stretching region using standard 10–15 cm^–1^ (Northwestern University, NU)^23^,^43^–^45^ and 0.6 cm^–1^ (Pacific Northwestern National Laboratory, PNNL)^21^,^22^,^46^,^47^ spectral resolution broadband SFG laser systems. A comprehensive description of the sample configurations and experimental setups has been published previously.^8^,^18^,^24^,^45^ C–H SFG spectra of the molecular standards were collected by exposing a solid fused silica or calcium fluoride optical window to the equilibrium vapor pressure of the molecule of interest and subsequently measuring the vapor:solid interface. SFG spectra of synthetic SOM were measured in the C–H stretching region by pressing the Teflon filter containing the collected material to a solid fused silica optical substrate. Spectra were measured using the ssp polarization combination and at laboratory ambient temperature and relative humidity. The angles of incidence for the visible and IR beams were 45° and 55° from surface normal respectively for the high resolution system and 45° and 60° from surface normal respectively for the standard resolution system.

The standard resolution spectra reported are an average of 7 individual spectra each taken for 2 minutes each. For the standard resolution spectra, the IR energy distribution was accounted for by normalizing the data to the ppp-polarized nonresonant SFG signal of gold deposited onto fused silica. Frequencies were calibrated using a polystyrene film.^13^,^14^,^45^,^48^ The high-resolution spectra are reported as an average of 3–5 individual acquisitions each recorded for 5 minutes. The data points were binned by 5 points, or by 1.73 cm^–1^, in Igor Pro Version 6.11 (WaveMetrics, Lake Oswego, OR, USA). SFG intensities in the 0.6 cm^–1^ resolution spectra were normalized to the ppp-polarized nonresonant SFG response of clean z-cut α-quartz, and frequencies were calibrated again using polystyrene.

## Conflicts of interest

There are no conflicts to declare.

## Supplementary Material

Supplementary information
