# Supplementary material for: Synthesis and surface spectroscopy of α-pinene isotopologues and their corresponding secondary organic material
Source: Chem Sci. 2019 Jul 31;10(36):8390–8. doi: 10.1039/c9sc02399b (PMC6844218; doi:10.1039/c9sc02399b)
Supplement: Supplementary file 1 [file SC-010-C9SC02399B-s001.pdf]

Electronic Supplementary Material (ESI) for Chemical Science.
This journal is © The Royal Society of Chemistry 2019

Synthesis and Surface Spectroscopy of α-Pinene Isotopologues and their Corresponding Secondary Organic Material

Mary Alice Upshur,[a,‖] Marvin M. Vega,[a,‖] Ariana Gray Bé,[a,‖] Hilary M. Chase,[a] Yue Zhang,[b] Aashish Tuladhar,[c] Zizwe A. Chase,[c] Fu, L.,[c] Carlena J. Ebben,[a] Zheming Wang,[c] Scot T. Martin,[b,d] Franz M. Geiger[a,]* and Regan J. Thomson[a,]*

[a.] *Department of Chemistry, Northwestern University, Evanston, IL 60208, USA*
[b.] *John A. Paulson School of Engineering and Applied Sciences, Harvard University, Cambridge, MA 02138, USA*
[c.] *William R. Wiley Environmental Molecular Sciences Laboratory, Pacific Northwest National Laboratory, Richland, WA 99352, USA*
[d.] *Department of Earth and Planetary Sciences, Harvard University, Cambridge, MA 02138, USA*

* Corresponding authors: r-thomson@northwestern.edu, f-geiger@northwestern.edu
‖ These authors contributed equally to this work.

Supporting Information

1. General Methods S1
2. Experimental Procedures for the Synthesis of Isotopologues S2
3. ^{1}H and ^{13}C NMR Spectra S16
4. IR and Raman Spectra S41
5. High Resolution Vapor-Phase SFG spectra on CaF_2 S42
6. Standard Resolution Vapor-Phase SFG spectra on fused silica S43
7. Details on SOM collection S44
8. References S45

1. General Methods. All reactions were carried out under a nitrogen atmosphere in flame-dried glassware with magnetic stirring unless otherwise stated. THF, Et_2O and CH_2Cl_2 were purified by passage through a bed of activated alumina.[1] Reagents were purified prior to use unless otherwise stated following the guidelines of Armarego and Chai.[2] Purification of reaction products was carried out by flash chromatography using EM Reagent silica gel 60 (230-400 mesh). Analytical thin layer chromatography was performed on EM Reagent 0.25 mm silica gel 60-F plates. Visualization was accomplished with UV light and anisaldehyde stain, ceric ammonium molybdate stain, or potassium permanganate stain followed by heating. Infrared spectra were recorded using a Bruker Tensor ATR spectrometer. ^{1}H-NMR spectra were recorded on a Bruker Avance III 500 (500 MHz) or a Varian Inova 400 (400 MHz) spectrometer and are reported in ppm using solvent as an internal standard ($CDCl_3$ at 7.26 ppm). Data are reported as (app = apparent, obs = obscured, s = singlet, d = doublet, t = triplet, q = quartet, p = pentet, h = hextet, sep = septet, o = octet, m = multiplet, b = broad; coupling constant(s) in Hz; integration. Proton-decoupled 13C-NMR spectra were recorded on a Bruker Avance III 500 (500 MHz) spectrometer and are reported in ppm using solvent as an internal standard ($CDCl_3$ at 77.00 ppm). Mass spectrometric data were obtained on an Agilent 6210 Time-of-Flight LC/MS and a Thermo Finnegan Mat 900 XL High Resolution Magnetic Sector.

2. Experimental Procedures for Synthesis of Isotopologues

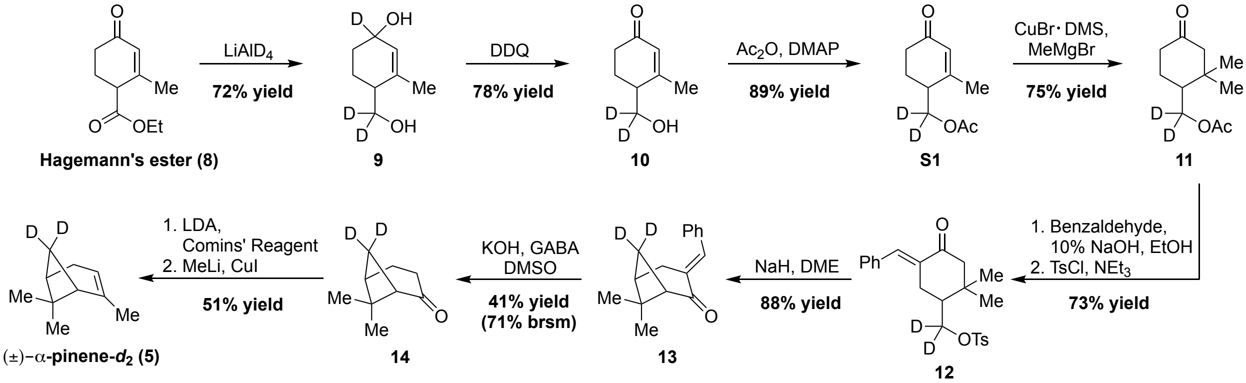

Scheme S1. Synthetic route to (±)-α-pinene-d_2 (**5**).

4-[Hydroxyl(*d*2)methyl]-3-methyl(1-*d*)cyclohex-2-en-1-ol (9):

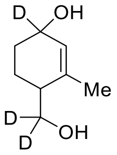

Hagemann's Ester (**8**)[3, 4] (6.98 g, 38.3 mmol) in Et_2O (40 mL) was added dropwise to a stirred solution of $LiAlD_4$ (1.76 g, 42.1 mmol) in Et_2O (140 mL) at 0 °C under N_2. After stirring for 3 hours, the reaction was carefully quenched with saturated Na_2SO_4, until bubbling subsided. The reaction was warmed to room temperature and stirred for 30 minutes and filtered over celite. Filtrate was dried with $MgSO_4$ and concentrated under reduced pressure. Flash column chromatography on silica gel using 100% EtOAc as the eluent afforded the title compound (4.00 g, 27.6 mmol, 72% yield, >99% deuterium incorporation) as a clear oil: IR (neat): 3295, 3035, 2934, 2864, 2199, 2091, 1086 cm^{-1}; Diastereomer A: ^1H NMR (500 MHz, Chloroform-*d*) δ 5.65 (t, *J* = 1.6 Hz, 1H), 2.11 (t, *J* = 5.5 Hz, 1H), 1.91 – 1.81 (m, 1H), 1.80 – 1.72 (m, 4H), 1.72 – 1.65 (m, 2H); ^{13}C NMR (126 MHz, Chloroform-*d*) δ 138.09, 128.16, 66.57 – 64.64 (m, 1C), 63.39 (dt, *J* = 43.5, 21.7 Hz, 1C), 41.51, 29.56, 22.17, 21.92; Diastereomer B: ^1H NMR (500 MHz, Chloroform-*d*) δ 5.61 (t, *J* = 1.7 Hz, 1H), 2.17 (t, *J* = 6.2 Hz, 1H), 1.96 (ddd, *J* = 12.6, 9.0, 3.1 Hz, 1H), 1.84 (dddd, *J* = 14.4, 8.9, 5.5, 3.2 Hz, 1H), 1.74 (t, *J* = 1.1 Hz, 3H), 1.66 (dddd, *J* = 13.5, 9.6, 6.6, 3.1 Hz, 1H), 1.50 (ddd, *J* = 12.7, 9.2, 3.3 Hz, 1H); ^{13}C NMR (126 MHz, Chloroform-*d*) δ 137.6, 128.5, 65.9 – 65.3 (m, 1C), 63.2 (p, *J* = 21.7 Hz, 1C), 41.6, 29.8, 22.1, 22.0; HRMS (EI): Exact mass calcd for $C_8H_9D_3O$ [M-H_2O] 127.1076. Found 127.1063.

4-[Hydroxyl(*d*2)methyl]-3-methylcyclohex-2-en-1-one (10):

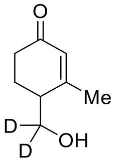

2,3-Dichloro-5,6-dicyano-1,4-benzoquinone (DDQ) (12.4 g, 54.6 mmol) was added to a stirred solution of 4-[hydroxyl(*d*2)methyl]-3-methyl(1-*d*)cyclohex-2-en-1-ol (**9**) (6.10 g, 42.0 mmol) in dioxane (140 mL). After 18 hours, the reaction mixture was concentrated under reduced pressure

and the resulting residue was diluted with chloroform and filtered over celite. Concentration under reduced pressure and flash column chromatography on silica gel using 60% EtOAc in hexanes as the eluent afforded the title compound (4.66 g, 32.8 mmol, 78% yield, >99% deuterium incorporation) as a brown/red oil: IR (neat): 3392, 3027, 2947, 2205, 2102, 1646, 1114 cm^{-1}; ^{1}H NMR (500 MHz, Chloroform-*d*) δ 5.94 (t, *J* = 1.5 Hz, 1H), 2.52 (ddd, *J* = 17.2, 8.8, 6.4 Hz, 1H), 2.47 (t, *J* = 5.3 Hz, 1H), 2.34 (dt, *J* = 17.2, 6.1 Hz, 1H), 2.16 – 2.07 (m, 2H), 2.02 (s, 3H), 1.60 (s, 1H); ^{13}C NMR (126 MHz, Chloroform-*d*) δ 199.6, 162.2, 128.8, 62.7 (p, *J* = 21.9 Hz, 1C), 42.3, 34.6, 25.3, 23.2; HRMS (ESI): Exact mass calcd for $C_8H_{11}D_2O_2$ $[M+H]^+$ 143.1036, Found 143.1036.

(2-Methyl-4-oxocyclohex-2-en-1-yl)(*d*2)methyl acetate (S1):

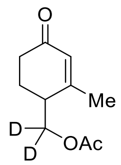

4-Dimethylaminopyridine (0.129 g, 1.06 mmol) was added to a stirred solution of 4-[hydroxyl(*d*2)methyl]-3-methylcyclohex-2-en-1-one (**10**) (3.00 g, 21.1 mmol) in dichloromethane (70 mL) followed by acetic anhydride (2.80 g, 27.4 mmol, 2.6 mL). After 12 hours, reaction was concentrated under reduced pressure. Flash column chromatography on silica gel using 20% EtOAc in hexanes as the eluent afforded the title compound (3.46 g, 18.78 mmol, 89% yield, >99% deuterium incorporation) as a clear oil: IR (neat): 3027, 2948, 2873, 2240, 1735, 1664, 1246 cm^{-1}; ^{1}H NMR (500 MHz, Chloroform-*d*) δ 5.92 (p, *J* = 1.4 Hz, 1H), 2.58 (t, *J* = 5.4 Hz, 1H), 2.48 (ddd, *J* = 17.2, 9.9, 5.2 Hz, 1H), 2.32 (ddd, *J* = 17.2, 7.1, 5.2 Hz, 1H), 2.15 – 2.04 (m, 1H), 2.05 (s, 3H), 2.03 – 1.96 (m, 1H), 1.98 (t, *J* = 1.1 Hz, 3H); ^{13}C NMR (126 MHz, Chloroform-*d*) δ 198.9, 171.1, 160.7, 129.1, 63.7 (p, *J* = 22.7 Hz, 1C), 39.2, 34.5, 25.6, 23.0, 21.1; HRMS (ESI): Exact mass calcd for $C_{10}H_{13}D_2O_3$ $[M+H]^+$ 185.1141. Found 185.1147.

(2,2-Dimethyl-4-oxocyclohexyl)(*d*2)methyl acetate (11):

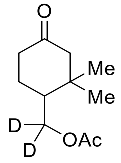

Methylmagnesium bromide (2.3 M in Et_2O, 3.35 g, 28.1 mmol) was added to a solution of CuBr•DMS (3.85 g, 18.7 mmol) in THF (55 mL) at −30 °C. After stirring for 15 minutes, a solution of (2-methyl-4-oxocyclohex-2-en-1-yl)(*d*2)methyl acetate (**S1**) (3.45 g, 18.7 mmol) in THF (20 mL) was cannulated dropwise into the reaction mixture at −30 °C. After 30 minutes, the reaction was carefully quenched with sat. NH_4Cl and stirred for an additional 5 minutes at −30 °C. After warming to room temperature, the reaction was diluted with Et_2O and filtered through celite using a 1:1 mixture of Et_2O and sat. NH_4Cl. Filtrate was then transferred to a separatory funnel and the organic phase was collected and aqueous layer extracted with Et_2O. Combined organics were washed with brine and dried with Na_2SO_4. Concentration under reduced pressure and flash column chromatography on silica gel using 20% EtOAc in hexanes as the eluent afforded the title compound (2.81 g, 14.0 mmol, 75% yield, >99% deuterium incorporation) as a clear oil: IR (neat): 2960, 2876, 2242, 2179, 1733, 1712, 1245 cm^{-1}; ^{1}H NMR (500 MHz, Chloroform-*d*) δ 2.38 (dddd, *J* = 14.5, 5.6, 3.7, 2.2 Hz, 1H), 2.35 – 2.27 (m, 1H), 2.28 (d, *J* =

13.8 Hz, 1H), 2.16 – 2.02 (m, 2H), 2.07 (s, 3H), 1.94 (dd, J = 11.4, 3.8 Hz, 1H), 1.66 (dtd, J = 13.8, 12.0, 5.4 Hz, 1H), 1.11 (s, 3H), 0.86 (s, 3H); ^{13}C NMR (126 MHz, Chloroform-*d*) δ 211.0, 171.4, 64.5 (p, J = 22.7 Hz, 1C), 56.1, 44.2, 40.4, 37.7, 30.01, 26.1, 21.6, 21.2; HRMS (ESI): Exact mass calcd for $C_{11}H_{17}D_2O_3$ $[M+H]^+$ 201.1454. Found 201.1457.

[(5*E*)-2,2-Dimethyl-4-oxo-5-(phenylmethylidene)cyclohexyl](*d*2)methyl 4-methylbenzene-1-sulfonate (12):

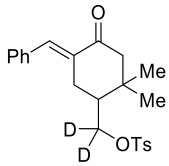

Benzaldehyde (5.78 g, 54.5 mmol) was added to a solution of (2,2-dimethyl-4-oxocyclohexyl)(*d*2)methyl acetate (**11**) (6.10 g, 24.8 mmol) in ethanol (75 mL) followed by a 10% NaOH solution (12.4 mL). After stirring for 12 hours, the reaction was diluted with H_2O and transferred to a separatory funnel. The organic phase was collected and the aqueous layer extracted with Et_2O. Combined organics were dried with $MgSO_4$ and concentrated under reduced pressure to yield the desired protected ketone, which was carried forward without further purification. Triethylamine (5.01 g, 49.5 mmol, 6.90 mL) was added to a solution of the crude material (6.10 g, 24.8 mmol) in dichloromethane (120 mL) followed by TsCl (7.08 g, 37.2 mmol). After stirring for 12 hours, the reaction was diluted with H_2O and transferred to a separatory funnel. The organic phase was collected and the aqueous layer extracted with dichloromethane. Combined organics were washed with a solution of 1:4 1 M HCl:H_2O followed by brine and dried over $MgSO_4$. Concentration under reduced pressure yielded a yellow solid. Ethanol was added to the crude material and cooled to 0 °C for 15 minutes. Filtration and rinsing cold ethanol afforded the title compound (7.24 g, 18.1 mmol, 73% yield over two steps, >99% deuterium incorporation) as a white powder: IR (neat): 3052, 2961, 2889, 2245, 1677, 1603, 1179 cm^{-1}; ^{1}H NMR (500 MHz, Chloroform-*d*) δ 7.75 (d, J = 8.3 Hz, 2H), 7.51 (t, J = 2.3 Hz, 1H), 7.45 – 7.35 (m, 5H), 7.30 (d, J = 8.0 Hz, 2H), 3.08 (ddd, J = 17.0, 5.3, 1.9 Hz, 1H), 2.56 (ddd, J = 17.0, 10.9, 2.8 Hz, 1H), 2.44 (s, 3H), 2.37 – 2.26 (m, 2H), 1.98 (dd, J = 11.0, 5.3 Hz, 1H), 1.03 (s, 3H), 0.95 (s, 3H); ^{13}C NMR (126 MHz, $CDCl_3$) δ 200.4, 145.2, 137.1, 135.4, 133.3, 133.1, 130.8, 130.1, 129.3, 128.7, 128.0, 54.6, 43.0, 33.9, 29.9, 29.2, 28.8, 22.3, 21.9; HRMS (ESI): Exact mass calcd for $C_{23}H_{25}D_2O_4S$ $[M+H]^+$ 401.1750. Found 401.1749.

(3E)-6,6-Dimethyl-3-(phenylmethylidene) (7,7,-*d*2)bicyclo[3.1.1]heptan-2-one (13):

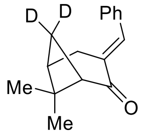

NaH (1.45 g, 36.3 mmol, 60% dispersion in mineral oil) was added to a solution of [(5*E*)-2,2-dimethyl-4-oxo-5-(phenylmethylidene)cyclohexyl](*d*2)methyl 4-methylbenzene-1-sulfonate (**12**) (7.26 g, 18.13 mmol) in dimethoxyethane (155 mL). The reaction was heated to 80 °C and stirred for 1 hour. After cooling to room temperature, the reaction was concentrated under reduced pressure. The resulting residue was diluted with Et_2O (75 mL) and H_2O (75 mL) and transferred to a separatory funnel. The organic phase was collected and the aqueous phase was extracted with Et_2O. Combined organics were washed with brine and dried with $MgSO_4$.

Concentration under reduced pressure and flash column chromatography on silica gel using 5% EtOAc in hexanes as the eluent afforded the title compound (3.64 g, 16.0 mmol, 88% yield, >99% deuterium incorporation) as a clear oil: IR (neat): 3046, 2952, 2936, 2255, 2197, 1684, 1608 cm^{-1}; ^1H NMR (500 MHz, Chloroform-*d*) δ 7.71 (t, J = 2.6 Hz, 1H), 7.62 – 7.57 (m, 2H), 7.44 – 7.39 (m, 2H), 7.38 – 7.33 (m, 1H), 2.99 (q, J = 2.4 Hz, 2H), 2.70 (d, J = 5.6 Hz, 1H), 2.36 (dt, J = 6.0, 3.2 Hz, 1H), 1.38 (s, 3H), 0.94 (s, 3H); ^{13}C NMR (126 MHz, Chloroform-*d*) δ 203.8, 136.0, 135.9, 132.9, 131.0, 129.1, 128.8, 31.1, 27.0 (p, J = 22.2 Hz, 1C), 26.5, 21.9; HRMS (ESI): Exact mass calcd for $C_{16}H_{17}D_2O$ $[M+H]^+$ 229.1556. Found 229.1554.

6,6-Dimethyl(7,7,-*d*2)bicyclo[3.1.1]heptan-2-one (nopinone-*d*2) (14):

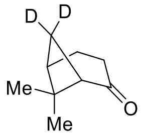

7 M KOH (4.8 mL) was added to a solution of (3E)-6,6-dimethyl-3-(phenylmethylidene) (7,7,-*d*2)bicyclo[3.1.1]heptan-2-one (**13**) (0.76 g, 3.33 mmol) and gamma-aminobutyric acid (GABA) (0.086 g, 0.83 mmol) in dimethylsulfoxide (48 mL). The reaction was heated to 100 °C and stirred for 45 minutes. After cooling to room temperature, the reaction was diluted with H_2O and transferred to a separatory funnel. The organic phase was collected and the aqueous phase was extracted with Et_2O. Combined organics were washed with brine and dried with $MgSO_4$. Concentration under reduced pressure and flash column chromatography on silica gel using 5% Et_2O in pentanes as the eluent afforded the title compound (190 mg, 1.37 mmol, 41% yield, 71% brsm, >99% deuterium incorporation) as a clear oil and starting material (320 mg, 1.40 mmol) as a yellow oil: IR (neat): 2929, 2871, 2246, 2197, 1717, 1702, 1459 cm^{-1}; ^1H NMR (500 MHz, Chloroform-*d*) δ 2.62 – 2.53 (m, 2H), 2.34 (ddd, J = 19.1, 9.2, 2.0 Hz, 1H), 2.25 – 2.20 (m, 1H), 2.05 (dddd, J = 13.3, 11.2, 4.0, 2.0 Hz, 1H), 1.94 (dddd, J = 13.5, 8.9, 6.4, 2.1 Hz, 1H), 1.33 (s, 3H), 0.85 (s, 3H); ^{13}C NMR (126 MHz, Chloroform-*d*) δ 215.3, 127.1, 58.0, 40.4, 33.0, 26.1, 24.8 (dt, J = 42.7, 21.3 Hz, 1C), 22.3, 21.6; HRMS (EI): Exact mass calcd for $C_9H_{12}D_2O$ [M] 140.1170. Found 140.1166.

2,6,6-Trimethyl(7,7-*d*2)bicyclo[3.1.1]hept-2-ene ((±)-α-pinene-*d*2) (5):

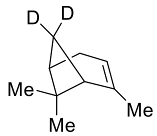

To a solution of diisopropylamine (0.82 mL, 5.9 mmol) in THF (12 mL) at 0 ºC was added *n*-BuLi (2.35 mL, 5.9 mmol, 2.5 M in hexanes) and cooled to –78 ºC, under N_2. After 15 minutes, 6,6-dimethyl(7,7-*d*2)bicyclo[3.1.1]heptan-2-one (**14**) (0.54 g, 3.9 mmol) in THF (8 mL) was added dropwise into the solution of LDA and stirred for 1 hour. At this time, a solution of Comins' reagent (3.07 g, 7.81 mmol) in THF (6 mL) was added over a period of 10 minutes. The resulting mixture was warmed to 0 ºC and stirred for 2 hours. Reaction was diluted with H_2O and Et_2O and transferred to a separatory funnel. The organic phase was collected and the aqueous layer extracted with Et_2O. The combined organics were dried with Na_2SO_4. Concentration under reduced pressure and flash column chromatography on silica gel in 3% EtOAc in hexanes as the eluent afforded the triflate intermediate (**15**). Methyl lithium (8.1 mL, 10.5 mmol, 1.3 M in Et_2O) was added to a slurry of CuI (1.43 g, 7.50 mmol) in THF (14 mL) at

0 ºC. After stirring for 10 minutes, a room temperature solution of the triflate intermediate (0.82 g, 3.0 mmol) in THF (6 mL) was added dropwise by cannula. Reaction was cooled to –5 ºC and stirred overnight. After 14 hours, the reaction was warmed to room temperature and filtered through a plug of Florisil®, flushing with pentanes. Concentration under reduced pressure afforded the title compound (270 mg, 1.99 mmol, 51% yield over two steps, >99% deuterium incorporation) as a clear oil: IR (neat): 3025, 2917, 2870, 2243, 2194, 1447, 779 cm^{-1}; ^{1}H NMR (500 MHz, Chloroform-*d*) δ 5.19 (tq, *J* = 3.3, 1.6 Hz, 1H), 2.19 (qq, *J* = 17.3, 2.6 Hz, 2H), 2.10 – 1.99 (m, 1H), 1.92 (d, *J* = 5.8 Hz, 1H), 1.66 (q, *J* = 2.0 Hz, 3H), 1.26 (s, 3H), 0.83 (s, 3H); ^{13}C NMR (126 MHz, Chloroform-*d*) δ 144.7, 116.2, 47.0, 40.7, 38.2, 31.4, 31.3 – 30.1 (m, 1C), 26.6, 23.2, 21.0; HRMS (EI): Exact mass calcd for $C_{10}H_{14}D_2$ [M] 138.1378. Found 138.1369.

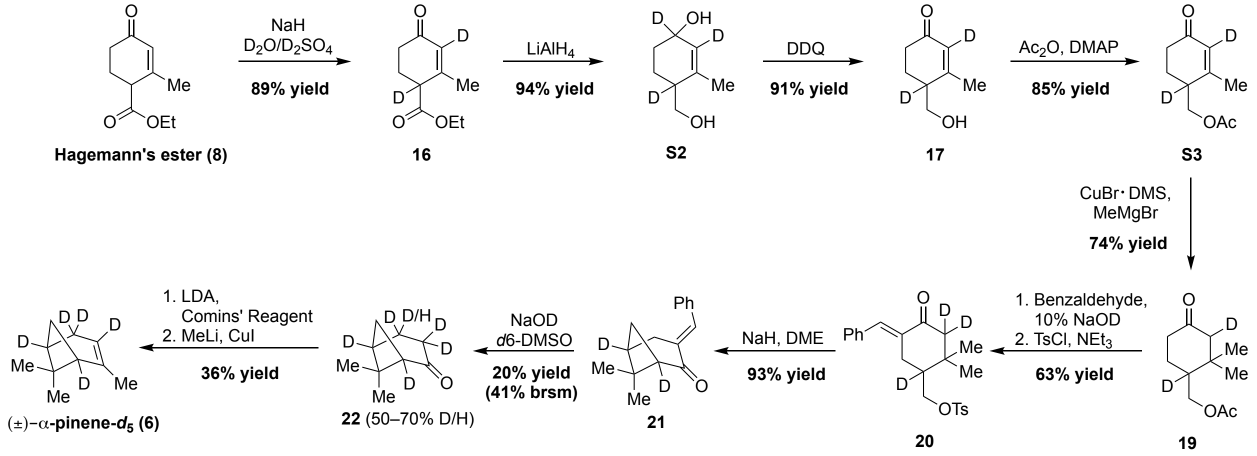

Scheme S2. Synthetic route to (±)-α-pinene-d_5 (**6**).

Ethyl 2-methyl-4-oxo(1,3-*d*2)cyclohex-2-ene-1-carboxylate (16):

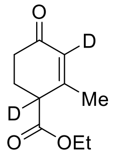

Hagemann's ester (**8**) (15.4 g, 84.5 mmol) in THF (50 mL) was cannulated dropwise into a stirred solution of NaH (13.5 g, 0.338 mol, 60% dispersion in mineral oil) in THF (230 mL) at 0 °C. After stirring for 1 hour, reaction was warmed to room temperature and stirred an additional 30 minutes. At this time, the reaction was cooled to 0 °C again and carefully quenched with D_2O until bubbling subsided. Reaction was then brought to pH 2 by adding concentrated D_2SO_4 (99.5%) dropwise until the color of the reaction solution changed from brown to light yellow. Reaction was warmed to room temperature and stirred for 1 hour, adding D_2SO_4 dropwise when necessary to maintain a pH of around 2. At this time, reaction mixture was filtered over a pad of silica gel using 100% EtOAc. The resulting filtrate was dried over $MgSO_4$ and concentrated under reduced pressure. Flash column chromatography on silica gel using 15% EtOAc in hexanes as the eluent afforded the title compound (13.9 g, 75.2 mmol, 89% yield, 99 ± 0.5% deuterium incorporation) as a yellow oil: IR (neat): 2979, 2258, 1725, 1644, 1618, 1240, 1178, 1075, 1022 cm^{-1}; ^{1}H NMR (500 MHz, Chloroform-*d*) δ 4.18 (q, J=7.1 Hz, 2H), 2.61-2.47 (m, 1H), 2.39-2.25 (m, 2H), 2.18 (m, 1H), 2.00 (s, 3H), 1.27 (t, J=7.1 Hz, 3H); ^{13}C NMR (126 MHz,

Chloroform-*d*) δ 198.4, 171.6, 156.9, 128.2 (m, 1C), 61.4, 45.6 (m, 1C), 34.2, 26.0, 23.4, 14.2; HRMS (EI): Exact mass calcd for $C_{10}H_{12}D_2O_3$ $[M+H]^+$ 185.1141. Found 185.1149.

4-[Hydroxylmethyl]-3-methyl(1,2,4-*d*3)cyclohex-2-en-1-ol (S2):

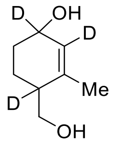

Ethyl 2-methyl-4-oxo(1,3-*d*2)cyclohex-2-ene-1-carboxylate (**16**) (6.8 g, 36.91 mmol) in Et_2O (30 mL) was added dropwise to a stirred solution of $LiAlH_4$ (1.68 g, 44.29 mmol) in Et_2O (90 mL) at 0 °C under N_2. After stirring for 3 hours, the reaction was carefully quenched with saturated Na_2SO_4, until bubbling subsided. The reaction was warmed to room temperature and stirred for 30 minutes and filtered over celite. Filtrate was dried with $MgSO_4$ and concentrated under reduced pressure. Flash column chromatography on silica gel using 80→100% EtOAc as the eluent afforded the title compound (5.00 g, 34.7 mmol, 94% yield, 99 ± 0.5% deuterium incorporation) as a clear oil: IR (neat): 3297, 2931, 2863, 2234, 2124, 2090, 1446, 1050, 1008 cm^{-1}; Diastereomer A: ^1H NMR (500 MHz, Chloroform-*d*) δ 4.12 (s, 1H), 3.61-3.75 (m, 1H) 2.14 (s, 1H), 1.81 – 1.57 (m, 6H); ^{13}C NMR (126 MHz, Chloroform-*d*) δ 137.17, 127.61 (m, 1C), 65.71, 63.65, 41.18 (m, 1C), 29.60, 21.94, 21.75; Diastereomer B: ^1H NMR (500 MHz, Chloroform-*d*) δ 4.11 (s, 1H), 3.61 – 3.75 (m, 1H), 2.07 (s, 1H), 1.81 – 1.57 (m, 6H); ^{13}C NMR (126 MHz, Chloroform-*d*) δ 137.7, 128.2 (m, 1C), 65.6, 63.6, 40.8 (m, 1C), 29.5, 21.9, 21.6; HRMS (EI): Exact mass calcd for $C_8H_9D_3O$ $[M-H_2O]$ 127.1076. Found 127.1063.

4-[Hydroxylmethyl]-3-methyl(2,4-*d*2)cyclohex-2-en-1-one (17):

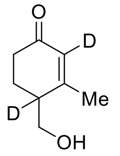

2,3-Dichloro-5,6-dicyano-1,4-benzoquinone (DDQ) (9.44 g, 41.6 mmol) was added to a stirred solution of 4-[hydroxylmethyl]-3-methyl(1,2,4-*d*3)cyclohex-2-en-1-ol (**S2**) (5.00 g, 34.7 mmol) in dioxane (115 mL). After 18 hours, the reaction mixture was concentrated under reduced pressure and the resulting residue was diluted with chloroform and filtered over celite. Concentration under reduced pressure and flash column chromatography on silica gel using 60% EtOAc in hexanes as the eluent afforded the title compound (4.49 g, 31.6 mmol, 91% yield, 99 ± 0.5% deuterium incorporation) as a brown/red oil: IR (neat): 3390, 3007, 2935, 2871, 2233, 2132, 1646, 1043, 747 cm^{-1}; ^1H NMR (500 MHz, Chloroform-*d*) δ 3.82 (q, J = 7.0 Hz, 2H), 2.50 (m, 1H), 2.32 (m, 1H), 2.12 – 2.06 (m, 2H), 2.00 (s, 3H), 1.71 (s, 1H); ^{13}C NMR (126 MHz, Chloroform-*d*) δ 199.6, 162.2, 128.2 (m, 1C), 63.1, 41.7 (m, 1C), 34.3, 24.8, 23.0; HRMS (ESI): Exact mass calcd for $C_8H_{11}D_2O_2$ $[M+H]^+$ 143.1036, Found 143.1036.

[2-Methyl-4-oxo(1,3-*d*2)cyclohex-2-en-1-yl]methyl acetate (S3):

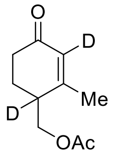

4-Dimethylaminopyridine (0.45 g, 3.69 mmol) was added to a stirred solution of 4-[hydroxylmethyl]-3-methyl(2,4-*d*2)cyclohex-2-en-1-one (**17**) (10.5 g, 73.8 mmol) in dichloromethane (240 mL) followed by acetic anhydride (9.80 g, 96.0 mmol, 9.07 mL). After 12 hours, reaction was concentrated under reduced pressure. Flash column chromatography on silica gel using 20% EtOAc in hexanes as the eluent afforded the title compound (11.6 g, 62.96 mmol, 85% yield, 99 ± 0.5% deuterium incorporation) as a clear oil: IR (neat): 2951, 2872, 2254, 2144, 1735, 1662, 1228, cm^{-1}; ^{1}H NMR (500 MHz, Chloroform-*d*) δ 4.21 (m, 2H), 2.47 (m, 1H), 2.32 (m, 1H), 2.15 – 2.04 (m, 1H), 2.05 (s, 3H), 2.03 – 1.96 (m, 1H), 1.98 (s, 3H); ^{13}C NMR (126 MHz, Chloroform-*d*) δ 198.8, 170.9, 160.4, 128.6, 129.1, 64.1, 38.7 (m, 1C), 34.2, 25.4, 22.8, 20.9; HRMS (ESI): Exact mass calcd for $C_{10}H_{13}D_2O_3$ $[M+H]^+$ 185.1141. Found 185.1147.

[2,2-Dimethyl-4-oxo(1,3-*d*2)cyclohexyl]methyl acetate (19):

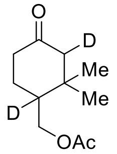

Methylmagnesium bromide (2.3 M in Et_2O, 74.9 mmol, 32.6 mL) was added to a solution of CuBr·DMS (10.3 g, 49.9 mmol) in THF (160 mL) at −30 °C. After stirring for 15 minutes, a solution of [2-methyl-4-oxo(1,3-*d*2)cyclohex-2-en-1-yl]methyl acetate (**S3**) (9.2 g, 49.9 mmol) in THF (50 mL) was cannulated dropwise into the reaction mixture at −30 °C. After 30 minutes, the reaction was carefully quenched with sat. NH_4Cl and stirred for an additional 5 minutes at −30 °C. After warming to room temperature, the reaction was diluted with Et_2O and filtered through celite using a 1:1 mixture of Et_2O and sat. NH_4Cl. Filtrate was then transferred to a separatory funnel and the organic phase was collected and aqueous layer extracted with Et_2O. Combined organics were washed with brine and dried with Na_2SO_4. Concentration under reduced pressure and flash column chromatography on silica gel using 20% EtOAc in hexanes as the eluent afforded the title compound (7.40 g, 37.0 mmol, 74% yield, 99 ± 0.5% deuterium incorporation) as a clear oil: IR (neat): 2960, 2875, 2178, 2134, 1735, 1710, 1232 cm^{-1}; ^{1}H NMR (500 MHz, Chloroform-*d*) δ 4.30 (d, J = 11.0 Hz, 1H), 3.88 (d, J = 11.0 Hz, 1H), 2.40 – 2.23 (m, 1H), 2.23 (s, 1H), 2.09 (m, 1H), 2.05 (s, 3H), 1.64 – 1.56 (m, 1H), 1.08 (s, 3H), 0.83 (s, 3H); ^{13}C NMR (126 MHz, Chloroform-*d*) δ 210.9, 171.1, 64.9, 55.6 (m, 1C), 43.6 (m, 1C), 40.2, 37.3, 29.8, 25.9, 21.3, 21.0; HRMS (ESI): Exact mass calcd for $C_{11}H_{17}D_2O_3$ $[M+H]^+$ 201.1454. Found 201.1457.

[(5*E*)-2,2-Dimethyl-4-oxo-5-(phenylmethylidene) (1,3,3-*d*3)cyclohexyl]methyl 4-methylbenzene-1-sulfonate (20):

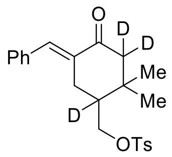

Benzaldehyde (11.6 g, 0.110 mol) was added to a solution of [2,2-dimethyl-4-oxo(1,3-*d*2)cyclohexyl]methyl acetate (**19**) (10.0 g, 49.9 mmol) in ethanol-*d* (100 mL) followed by a 10% NaOD solution (25 mL). After stirring for 12 hours, the reaction was concentrated under reduced pressure then diluted with D_2O and transferred to a separatory funnel. The organic phase was collected and the aqueous layer extracted with Et_2O. Combined organics were dried with $MgSO_4$

and concentrated under reduced pressure to yield the desired protected ketone, which was carried forward without further purification. Triethylamine (10.1 g, 99.9 mmol, 13.9 mL) was added to a solution of the crude material (12.3 g, 49.9 mmol) in dichloromethane (250 mL) followed by TsCl (14.3 g, 74.9 mmol). After stirring for 12 hours, the reaction was diluted with H_2O and transferred to a separatory funnel. The organic phase was collected and the aqueous layer extracted with dichloromethane. Combined organics were washed with a solution of 1:4 1 M HCl:H_2O followed by brine and dried over $MgSO_4$. Concentration under reduced pressure yielded a yellow solid. Ethanol was added to the crude material to form a thick slurry, which was then cooled to 0 °C for 30 minutes. Filtration and rinsing cold ethanol afforded the title compound (12.6 g, 31.4 mmol, 63% yield over two steps, 99 ± 0.5% deuterium incorporation) as a white powder: IR (neat): 3051, 2963, 2848, 2216, 2161, 1675, 1602, 1355, 1172 cm^{-1}; ^{1}H NMR (500 MHz, Chloroform-*d*) δ 7.75 (d, *J* = 8.2 Hz, 2H), 7.50 (t, *J* = 2.2 Hz, 1H), 7.43 – 7.36 (m, 5H), 7.31 (d, *J* = 8.0 Hz, 2H), 4.29 (d, *J* = 9.8 Hz, 1H), 3.99 (d, *J* = 9.9 Hz, 1H), 3.09 (dd, *J* = 17.0, 1.9 Hz, 1H), 2.57 (dd, *J* = 17.0, 2.8 Hz, 1H), 2.44 (s, 3H), 1.03 (s, 3H), 0.95 (s, 3H); ^{13}C NMR (126 MHz, $CDCl_3$) δ 200.2, 145.0, 136.8, 135.2, 133.1, 132.8, 130.6, 129.9, 129.0, 128.5, 127.8, 70.7, 53.6 (m, 1C), 42.4 (m, 1C), 33.4, 29.7, 28.9, 28.5, 22.0, 21.7; HRMS (ESI): Exact mass calcd for $C_{23}H_{25}D_2O_4S$ $[M+H]^+$ 401.1750. Found 401.1749.

(3*E*)-6,6-Dimethyl-3-(phenylmethylidene) (1,5-*d*2)bicyclo[3.1.1]heptan-2-one (21):

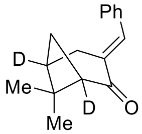

NaH (1.10 g, 27.5 mmol, 60% dispersion in mineral oil) was added to a solution of [(5*E*)-2,2-dimethyl-4-oxo-5-(benzylidene) (1,3,3-*d*3)cyclohexyl]methyl 4-methylbenzene-1-sulfonate (**20**) (5.5 g, 13.7 mmol) in dimethoxyethane (114 mL). The reaction was heated to 80 °C and stirred for 1 hour. After cooling to room temperature, the reaction was concentrated under reduced pressure. The resulting residue was diluted with Et_2O (X mL) and H_2O (X mL) and transferred to a separatory funnel. The organic phase was collected and the aqueous phase was extracted with Et_2O. Combined organics were washed with brine and dried with $MgSO_4$. Concentration under reduced pressure and flash column chromatography on silica gel using 5% EtOAc in hexanes as the eluent afforded the title compound (2.92 g, 12.8 mmol, 93% yield, 99 ± 0.5% deuterium incorporation) as a clear oil: IR (neat): 3044, 2962, 2868, 2203, 2185, 1680, 1607, 1269 cm^{-1}; ^{1}H NMR (500 MHz, Chloroform-*d*) δ 7.69 (d, *J* = 2.5 Hz, 1H), 7.59 (d, *J* = 7.7 Hz, 2H), 7.40 (dd, *J* = 8.4, 6.8 Hz, 2H), 7.34 (m, 1H), 2.94 (s, 1H), 2.60 (dd, *J* = 10.5, 1.8 Hz, 1H), 1.53 – 1.44 (m, 2H), 1.36 (s, 3H), 0.91 (s, 3H); ^{13}C NMR (126 MHz, Chloroform-*d*) δ 203.6, 135.8, 135.8, 132.6, 130.8, 128.9, 128.6, 55.5 (m, 1C), 40.6, 30.5 (m, 1C), 27.2, 26.1, 21.7; HRMS (ESI): Exact mass calcd for $C_{16}H_{17}D_2O$ $[M+H]^+$ 229.1556. Found 229.1554.

6,6-Dimethyl(1,3,3,4,5-*d*5)bicyclo[3.1.1]heptan-2-one (nopinone-*d*6) (22):

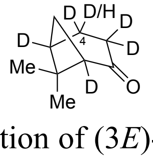

7 M NaOD (8.76 mL) was added to a solution of (3*E*)-6,6-dimethyl-3-(phenylmethylidene) (1,5-*d*2)bicyclo[3.1.1]heptan-2-one (**21**) (2.00 g, 8.76 mmol) in dimethylsulfoxide-*d*6 (25 mL). The reaction was heated to 90 °C and stirred for 45 minutes. After cooling to room temperature, the

reaction was diluted with D_2O and transferred to a separatory funnel. The organic phase was collected and the aqueous phase was extracted with Et_2O. Combined organics were dried with $MgSO_4$. Concentration under reduced pressure and flash column chromatography on silica gel using 5→10% Et_2O in pentanes as the eluent separated recovered starting material (1.04 g, 4.56 mmol, 52% yield) as a yellow oil and the title compound (250 mg, 1.73 mmol, 20% yield) as a clear oil. From several runs the percent incorporation of deuterium at C4 was 50–70%. IR (neat): 2970, 2953, 2870, 2210, 2193, 2113, 1702, 1265 cm^{-1}; ^1H NMR (500 MHz, Chloroform-*d*) δ 2.56 – 2.45 (m, 1H), 1.89 (s, 3:7 H:D, 1H), 1.58 – 1.50 (m, 1H), 1.30 (s, 3H), 0.83 (s, 3H); ^{13}C NMR (126 MHz, Chloroform-*d*) δ 215.2, 128.8 (m, 1C), 57.6 (m, 1C), 41.0, 25.8, 25.1, 25.0, 22.1, 20.8 (m, 1C); HRMS (EI): Exact mass calcd for $C_9H_{12}D_2O$ [M] 140.1170. Found 140.1166.

2,6,6-Trimethyl(1,3,4,5-*d*4)bicyclo[3.1.1]hept-2-ene ((±)-α-pinene-*d*5) (6):

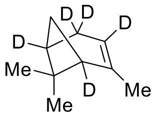

To a solution of diisopropylamine (0.53 mL, 3.80 mmol) in THF (8 mL) at 0 ºC was added *n*-BuLi (2.25 mL, 3.80 mmol, 1.7 M in hexanes) and cooled to –78 ºC, under N_2. After 15 minutes, 6,6-dimethyl(1,3,3,4,5-*d*5)bicyclo[3.1.1]heptan-2-one (**22**) (0.363 g, 2.53 mmol) in THF (4 mL) was added dropwise into the solution of LDA and stirred for 1 hour. At this time, a solution of Comins' reagent (1.97 g, 5.06 mmol) in THF (4 mL) was added over a period of 10 minutes. The resulting mixture was warmed to 0 ºC and stirred for 3 hours. Reaction was diluted with H_2O and Et_2O and transferred to a separatory funnel. The organic phase was collected and the aqueous layer extracted with Et_2O. The combined organics were dried with $MgSO_4$. Concentration under reduced pressure and flash column chromatography on silica gel in 3% EtOAc in hexanes as the eluent afforded the triflate intermediate. Methyl lithium (3.81 mL, 4.95 mmol, 1.3 M in Et_2O) was added to a slurry of CuI (0.674 g, 3.54 mmol) in THF (7 mL) at 0 ºC. After stirring for 10 minutes, a room temperature solution of the triflate intermediate (0.388 g, 1.42 mmol) in THF (3 mL) was added dropwise by cannula. Reaction was cooled to –5 ºC and stirred overnight. After 14 hours, the reaction was warmed to room temperature and filtered through a plug of Florisil®, flushing with pentanes. Concentration under reduced pressure followed by two more filtrations through Florisil® afforded the title compound (129 mg, 0.911 mmol, 36% yield over two steps) as a clear oil. The percent incorporation of deuterium at C4 was 50–70%. IR (neat): 2997, 2933, 2884, 2251, 2189, 1456, 1370 cm^{-1}; ^1H NMR (500 MHz, Chloroform-*d*) δ 2.30 (m, 1H), 2.11 (m, 1:1 H:D, 1H), 1.64 (s, 3H), 1.12 (dd, *J* = 8.4, 4.3 Hz, 1H), 1.24 (s, 3H), 0.81 (s, 3H); ^{13}C NMR (126 MHz, Chloroform-*d*) δ 144.4, 115.6 (m, 1C), 46.5 (m, 1C), 40.1 (m, 1C), 37.7, 31.2, 30.8 – 30.5 (m, 1C), 26.3, 22.9, 20.8; HRMS (EI): Exact mass calcd for $C_{10}H_{14}D_2$ [M] 138.1378. Found 138.1369.

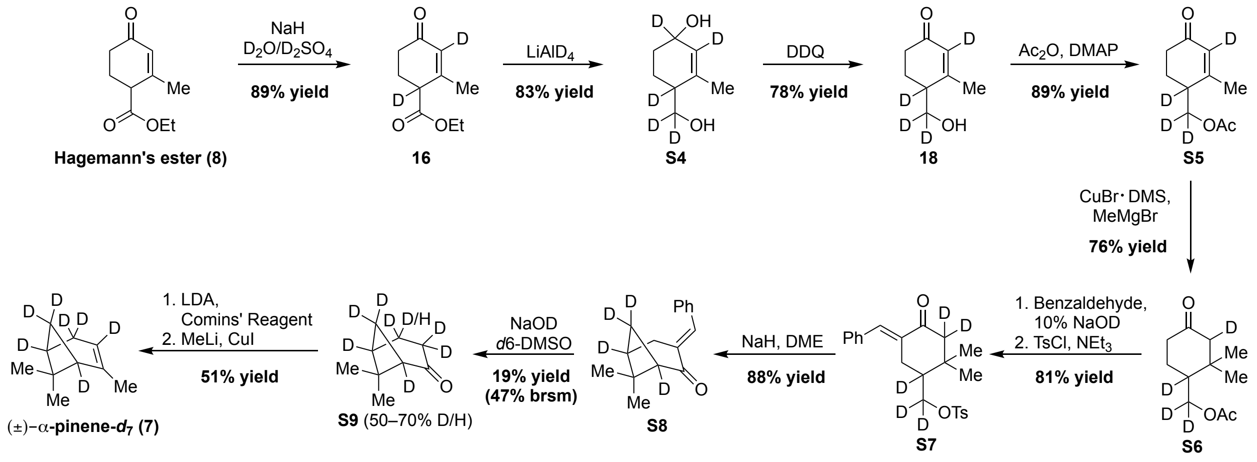

Scheme S3. Synthetic route to (±)-α-pinene-d_7 (**7**).

4-[Hydroxyl(*d*2)methyl]-3-methyl(1,2,4-*d*3)cyclohex-2-en-1-ol (S4):

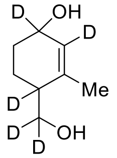

Ethyl 2-methyl-4-oxo(1,3-*d*2)cyclohex-2-ene-1-carboxylate (**16**) (10.1 g, 54.8 mmol) in Et_2O (40 mL) was added dropwise to a stirred solution of $LiAlD_4$ (2.1 g, 50.3 mmol) in Et_2O (150 mL) at 0 °C under N_2. After stirring for 3 hours, the reaction was carefully quenched with saturated Na_2SO_4, until bubbling subsided. The reaction was warmed to room temperature and stirred for 30 minutes and filtered over celite. Filtrate was dried with $MgSO_4$ and concentrated under reduced pressure. Flash column chromatography on silica gel using 80→100% EtOAc as the eluent afforded the title compound (6.70 g, 45.5 mmol, 83% yield, 99 ± 0.5% deuterium incorporation) as a clear oil: IR (neat): 3303, 2914, 2861, 2196, 2091, 1377, 972 cm^{-1}; Diastereomer A: ^{1}H NMR (500 MHz, Chloroform-*d*) δ 1.85 – 1.80 (m, 1H), 1.73 – 1.70 (m, 4H), 1.66 – 1.61 (m, 2H); ^{13}C NMR (126 MHz, Chloroform-*d*) δ 137.73, 127.97 (m, 1C), 66.39 – 64.96 (m, 1C), 63.42 – 62.74 (dt, J = 43.8, 21.5 Hz, 1C), 40.83 – 40.53 (m, 1C), 28.70 – 28.39 (m, 1C), 21.61, 21.34 – 21.06 (m, 1C); Diastereomer B: ^{1}H NMR (500 MHz, Chloroform-*d*) δ 1.85 – 1.80 (m, 1H), 1.73 – 1.70 (m, 4H), 1.66 – 1.61 (m, 2H); ^{13}C NMR (126 MHz, Chloroform-*d*) δ 137.3, 127.8 (m, 1C), 65.3 – 65.0 (m, 1C), 63.5 – 62.8 (p, J = 43.8, 21.5 Hz, 1C), 40.8 – 40.5 (m, 1C), 29.1 – 28.7 (m, 1C), 21.8, 21.3 – 21.1 (m, 1C); HRMS (EI): Exact mass calcd for $C_8H_9D_3O$ [M-H_2O] 127.1076. Found 127.1063.

4-[Hydroxyl(*d*2)methyl]-3-methyl(2,4-*d*2)cyclohex-2-en-1-one (18):

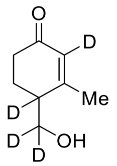

2,3-Dichloro-5,6-dicyano-1,4-benzoquinone (DDQ) (27.2 g, 120 mmol) was added to a stirred solution of 4-[hydroxyl(*d*2)methyl]-3-methyl(1,2,4-*d*3)cyclohex-2-en-1-ol (**S4**) (14.7 g, 99.9 mmol) in dioxane (300 mL). After 18 hours, the reaction mixture was concentrated under

reduced pressure and the resulting residue was diluted with chloroform and filtered over celite. Concentration under reduced pressure and flash column chromatography on silica gel using 60% EtOAc in hexanes as the eluent afforded the title compound (11.2 g, 77.9 mmol, 78% yield, 99 ± 0.5% deuterium incorporation) as a brown/red oil: IR (neat): 3389, 2946, 2869, 2203, 2080, 1645, 1123 cm^{-1}; ^{1}H NMR (500 MHz, Chloroform-*d*) δ 2.55 – 2.41 (m, 1H), 2.31 (dt, J = 17.3, 6.0 Hz, 1H) 2.15 – 2.05 (m, 2H), 2.00 (s, 3H), 1.69 (s, 1H); ^{13}C NMR (126 MHz, Chloroform-*d*) δ 199.5, 162.1, 128.2 (m, 1C), 62.7 (p, J = 21.8 Hz, 1C), 41.6 (m, 1C), 34.3, 24.9, 23.0; HRMS (ESI): Exact mass calcd for $C_8H_{11}D_2O_2$ $[M+H]^+$ 143.1036, Found 143.1036.

[2-Methyl-4-oxo(1,3-*d*2)cyclohex-2-en-1-yl](*d*2)methyl acetate (S5):

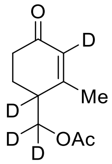

4-Dimethylaminopyridine (1.10 g, 9.00 mmol) was added to a stirred solution of 4-[hydroxyl(*d*2)methyl]-3-methyl(2,4-*d*2)cyclohex-2-en-1-one (**18**) (19.7 g, 0.140 mol) in dichloromethane (300 mL) followed by acetic anhydride (18.1 g, 0.180 mol, 16.8 mL). After 12 hours, reaction was concentrated under reduced pressure. Flash column chromatography on silica gel using 20% EtOAc in hexanes as the eluent afforded the title compound (23.2 g, 0.125 mol, 89% yield, 99 ± 0.5% deuterium incorporation) as a clear oil: IR (neat): 2945, 2872, 2243, 2191, 2122, 1735, 1661, 1248 cm^{-1}; ^{1}H NMR (500 MHz, Chloroform-*d*) δ 2.52 – 2.42 (m, 1H), 2.37 – 2.27 (m, 1H), 2.07 (m, 2H), 2.05 (s, 3H), 1.98 (s, 3H); ^{13}C NMR (126 MHz, Chloroform-*d*) δ 198.9, 170.9, 160.5, 128.5 (m, 1C), 63.5 (m, 1C), 38.5 (m, 1C), 34.2, 25.3, 22.8, 20.9; HRMS (ESI): Exact mass calcd for $C_{10}H_{13}D_2O_3$ $[M+H]^+$ 185.1141. Found 185.1147.

[2,2-Dimethyl-4-oxo(1,3-*d*2)cyclohexyl]methyl acetate (S6):

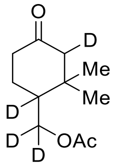

Methylmagnesium bromide (2.3 M in Et_2O, 19.8 g, 0.166 mol) was added to a solution of CuBr•DMS (22.74 g, 0.110 mol) in THF (350 mL) at −30 °C. After stirring for 15 minutes, a solution of [2-methyl-4-oxo(1,3-*d*2)cyclohex-2-en-1-yl](*d*2)methyl acetate (**S5**) (20.6 g, 0.110 mol) in THF (100 mL) was cannulated dropwise into the reaction mixture at −30 °C. After 30 minutes, the reaction was carefully quenched with sat. NH_4Cl and stirred for an additional 5 minutes at −30 °C. After warming to room temperature, the reaction was diluted with Et_2O and filtered through celite using a 1:1 mixture of Et_2O and sat. NH_4Cl. Filtrate was then transferred to a separatory funnel and the organic phase was collected and aqueous layer extracted with Et_2O. Combined organics were washed with brine and dried with Na_2SO_4. Concentration under reduced pressure and flash column chromatography on silica gel using 20% EtOAc in hexanes as the eluent afforded the title compound (16.9 g, 83.6 mmol, 76% yield, 99 ± 0.5% deuterium incorporation) as a clear oil: IR (neat): 2960, 2875, 2241, 2186, 2131, 1734, 1710, 1248 cm^{-1}; ^{1}H NMR (500 MHz, Chloroform-*d*) δ 2.40 – 2.23 (m, 2H), 2.15 – 2.00 (m, 2H), 2.05 (s, 3H), 1.08 (s, 3H), 0.83 (s, 3H); ^{13}C NMR (126 MHz, Chloroform-*d*) δ 210.9, 171.1, 64,2 (p, J = 22.6 Hz,

1C), 55.6 (m, 1C), 43.4 (m, 1C), 40.2, 37.3, 29.7, 25.8, 21.3, 21.0; HRMS (ESI): Exact mass calcd for $C_{11}H_{17}D_2O_3$ $[M+H]^+$ 201.1454. Found 201.1457.

[(5*E*)-2,2-Dimethyl-4-oxo-5-(phenylmethylidene) (1,3,3-d3)cyclohexyl](*d*2)methyl 4-methylbenzene-1-sulfonate (S7):

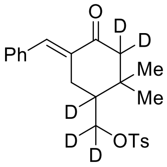

Benzaldehyde (18.2 g, 0.172 mol) was added to a solution of [2,2-dimethyl-4-oxo(1,3-*d*2)cyclohexyl]methyl acetate (**S6**) (15.8 g, 78.1 mmol) in ethanol-*d* (94 mL) followed by a 10% NaOD solution (39.5 mL). After stirring for 12 hours, the reaction was concentrated under reduced pressure then diluted with D_2O and transferred to a separatory funnel. The organic phase was collected and the aqueous layer extracted with Et_2O. Combined organics were dried with $MgSO_4$ and concentrated under reduced pressure to yield the desired protected ketone, which was carried forward without further purification. Triethylamine (16.8 g, 0.166 mol, 23.2 mL) was added to a solution of the crude material (19.4 g, 78.1 mmol) in dichloromethane (415 mL) followed by TsCl (23.8 g, 0.125 mol). After stirring for 12 hours, the reaction was diluted with H_2O and transferred to a separatory funnel. The organic phase was collected and the aqueous layer extracted with dichloromethane. Combined organics were washed with a solution of 1:4 1 M HCl:H_2O followed by brine and dried over $MgSO_4$. Concentration under reduced pressure yielded a yellow solid. Ethanol was added to the crude material until a slurry was formed and cooled to 0 °C for 15 minutes. Filtration and rinsing with cold ethanol afforded the title compound (25.5 g, 63.3 mmol, 81% yield over two steps, 99 ± 0.5% deuterium incorporation) as a white powder: IR (neat): 3052, 2962, 2883, 2848, 2251, 2216, 2160, 1675, 1603, 1355, 1171 cm^{-1}; ^1H NMR (500 MHz, Chloroform-*d*) δ 7.73 (d, J = 8.2 Hz, 2H), 7.49 (t, J = 2.2 Hz, 1H), 7.39 (m, 5H), 7.28 (d, J = 8.0 Hz, 2H), 3.07 (dd, J = 17.0, 1.9 Hz, 1H), 2.55 (dd, J = 17.0, 2.8 Hz, 1H), 2.42 (s, 3H), 1.01 (s, 3H), 0.93 (s, 3H); ^{13}C NMR (126 MHz, $CDCl_3$) δ 200.2, 144.9, 136.8, 135.2, 133.1, 132.9, 130.6, 129.9, 129.0, 128.5, 127.8, 53.6 (m, 1C), 42.2 (m, 1C), 33.4, 28.9, 28.5, 22.0, 21.7; HRMS (ESI): Exact mass calcd for $C_{23}H_{25}D_2O_4S$ $[M+H]^+$ 401.1750. Found 401.1749.

(3*E*)-6,6-Dimethyl-3-(phenylmethylidene) (1,5,7,7-*d*4)bicyclo[3.1.1]heptan-2-one (S8):

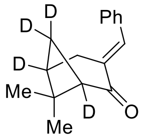

NaH (0.99 g, 24.8 mmol, 60% dispersion in mineral oil) was added to a solution of [(5*E*)-2,2-dimethyl-4-oxo-5-(benzylidene) (1,3,3-d3)cyclohexyl](*d*2)methyl 4-methylbenzene-1-sulfonate (**S7**) (5.0 g, 12.4 mmol) in dimethoxyethane (105 mL). The reaction was heated to 80 °C and stirred for 1 hour. After cooling to room temperature, the reaction was concentrated under reduced pressure. The resulting residue was diluted with Et_2O and H_2O and transferred to a separatory funnel. The organic phase was collected and the aqueous phase was extracted with Et_2O. Combined organics were washed with brine and dried with $MgSO_4$. Concentration under reduced pressure and flash column chromatography on silica gel using 5% EtOAc in hexanes as

the eluent afforded the title compound (2.54 g, 11.0 mmol, 89% yield, 99 ± 0.5% deuterium incorporation) as a clear oil: IR (neat): 3045, 2954, 2922, 2867, 2254, 2188, 1682, 1608, 1443, 1256 cm^{-1}; ^{1}H NMR (500 MHz, Chloroform-*d*) δ 7.69 (t, J = 2.6 Hz, 1H), 7.61 – 7.55 (m, 2H), 7.45 – 7.36 (m, 2H), 7.38 – 7.28 (m, 1H), 2.96 (t, J = 2.0 Hz, 2H), 1.36 (s, 3H), 0.91 (s, 3H); ^{13}C NMR (126 MHz, Chloroform-*d*) δ 203.6, 135.7, 135.7, 132.7, 130.8, 128.9, 128.6, 55.7, 40.6, 38.7 (m, 1C), 30.8, 26.2, 26.2, 21.7; HRMS (ESI): Exact mass calcd for $C_{16}H_{17}D_2O$ $[M+H]^+$ 229.1556. Found 229.1554.

6,6-Dimethyl(1,3,3,4,5,7,7-*d*6)bicyclo[3.1.1]heptan-2-one (nopinone-*d*8) (S9):

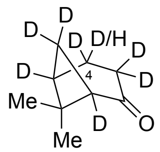

7 M NaOD (3.39 mL) was added to a solution of (3*E*)-6,6-dimethyl-3-(phenylmethylidene) (1,5,7,7-*d*4)bicyclo[3.1.1]heptan-2-one (**S8**) (1.3 g, 5.64 mmol) in dimethylsulfoxide-*d*6 (16 mL). The reaction was heated to 90 °C and stirred for 45 minutes. After cooling to room temperature, the reaction was diluted with D_2O and transferred to a separatory funnel. The organic phase was collected and the aqueous phase was extracted with Et_2O. Combined organics were dried with $MgSO_4$. Concentration under reduced pressure and flash column chromatography on silica gel using 5→10% Et_2O in pentanes as the eluent separated recovered starting material (0.779 g, 3.38 mmol, 60% recovery) as a yellow oil and the title compound (0.156 g, 1.07 mmol, 19% yield) as a clear oil. From several runs the percent incorporation of deuterium at C4 was 50–70%. IR (neat): 2956, 2925, 2870, 2246, 2188, 1717, 1653, 1559, 1457 cm^{-1}; ^{1}H NMR (500 MHz, Chloroform-*d*) δ 2.52 (s, 1:4 H:D, 1H), 1.88 (s, 1:1 H:D, 1H), 1.30 (s, 3H), 0.83 (s, 3H); ^{13}C NMR (126 MHz, Chloroform-*d*) δ 215.2, 128.4 (m, 1C), 57.5 (m, 1C), 40.9, 29.7, 25.8, 24.3 (m, 1C) 22.1, 20.9 (m, 1C); HRMS (EI): Exact mass calcd for $C_9H_{12}D_2O$ [M] 140.1170. Found 140.1166.

2,6,6-Trimethyl(1,3,4,5,7,7-*d*6)bicyclo[3.1.1]hept-2-ene ((±)-α-pinene-*d*7) (7):

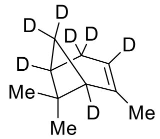

To a solution of diisopropylamine (0.61 mL, 4.38 mmol) in THF (9 mL) at 0 ºC was added *n*-BuLi (2.19 mL, 4.38 mmol, 2.0 M in hexanes) and cooled to –78 ºC, under N_2. After 15 minutes, 6,6-dimethyl(1,3,3,4,5,7,7-*d*6)bicyclo[3.1.1]heptan-2-one (**22**) (0.424 g, 2.92 mmol) in THF (6.5 mL) was added dropwise into the solution of LDA and stirred for 1 hour. At this time, a solution of Comins' reagent (2.29 g, 5.84 mmol) in THF (4.5 mL) was added over a period of 10 minutes. The resulting mixture was warmed to 0 ºC and stirred for 2 hours. Reaction was diluted with H_2O and Et_2O and transferred to a separatory funnel. The organic phase was collected and the aqueous layer extracted with Et_2O. The combined organics were dried with Na_2SO_4. Concentration under reduced pressure and flash column chromatography on silica gel in 3% EtOAc in hexanes as the eluent afforded the triflate intermediate. Methyl lithium (3.97 mL, 5.95 mmol, 1.5 M in Et_2O) was added to a slurry of CuI (0.810 g, 4.25 mmol) in THF (8 mL) at 0 ºC. After stirring for 10 minutes, a room temperature solution of the triflate intermediate (0.47 g, 1.70 mmol) in THF (11.5 mL) was added dropwise by cannula. Reaction was cooled to –5 ºC

and stirred overnight. After 14 hours, the reaction was warmed to room temperature and filtered through a plug of Florisil®, flushing with pentanes. Concentration under reduced pressure followed by two more filtrations through Florisil® afforded the title compound (0.212 g, 1.49 mmol, 51% yield over two steps) as a clear oil. The percent incorporation of deuterium at C4 was 50–70%. IR (gas): 2933, 2878, 2251, 2182, 2094, 1447, 1370 cm^{-1}; ^{1}H NMR (500 MHz, Chloroform-*d*) δ 2.10 (s, 1:1 H:D, 1H) 1.89 (s, 15:85 H:D, 1H), 1.64 (s, 3H), 1.24 (s, 3H), 0.81 (s, 3H); ^{13}C NMR (126 MHz, Chloroform-*d*) δ 144.4 (m, 1C), 115.6 (m, 1C), 46.3 (m, 1C), 39.9 (m, 1C), 37.7 (m, 1C), 31.0, 30.6 (m, 1C), 26.3, 22.9, 20.8; HRMS (EI): Exact mass calcd for $C_{10}H_{14}D_2$ [M] 138.1378. Found 138.1369.

3. ^{1}H and ^{13}C NMR Spectra

4-[Hydroxyl(*d*2)methyl]-3-methyl(1-*d*)cyclohex-2-en-1-ol (9):

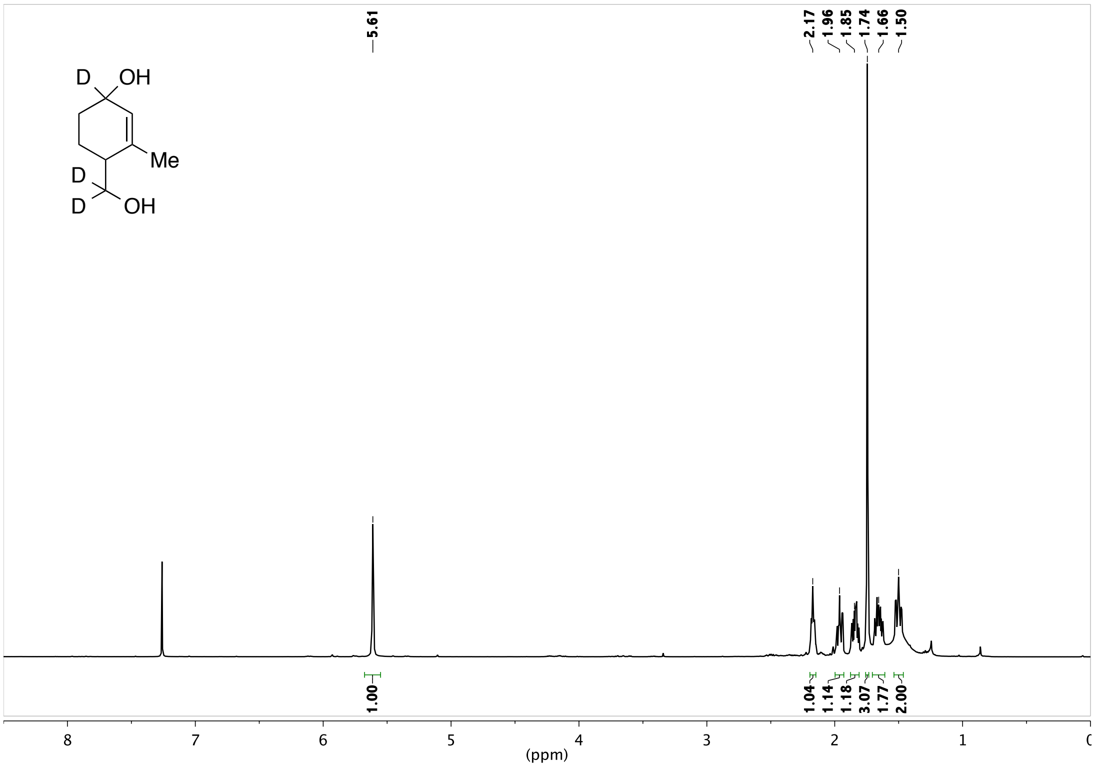

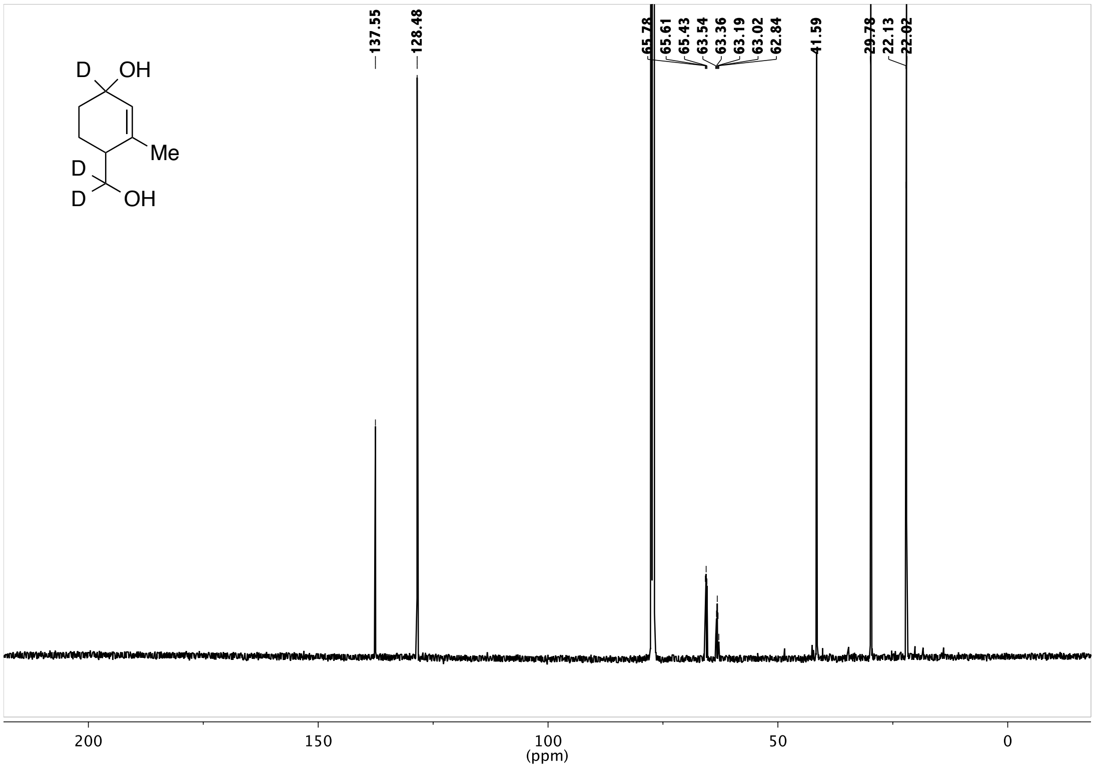

4-[Hydroxyl(*d*2)methyl]-3-methylcyclohex-2-en-1-one (10):

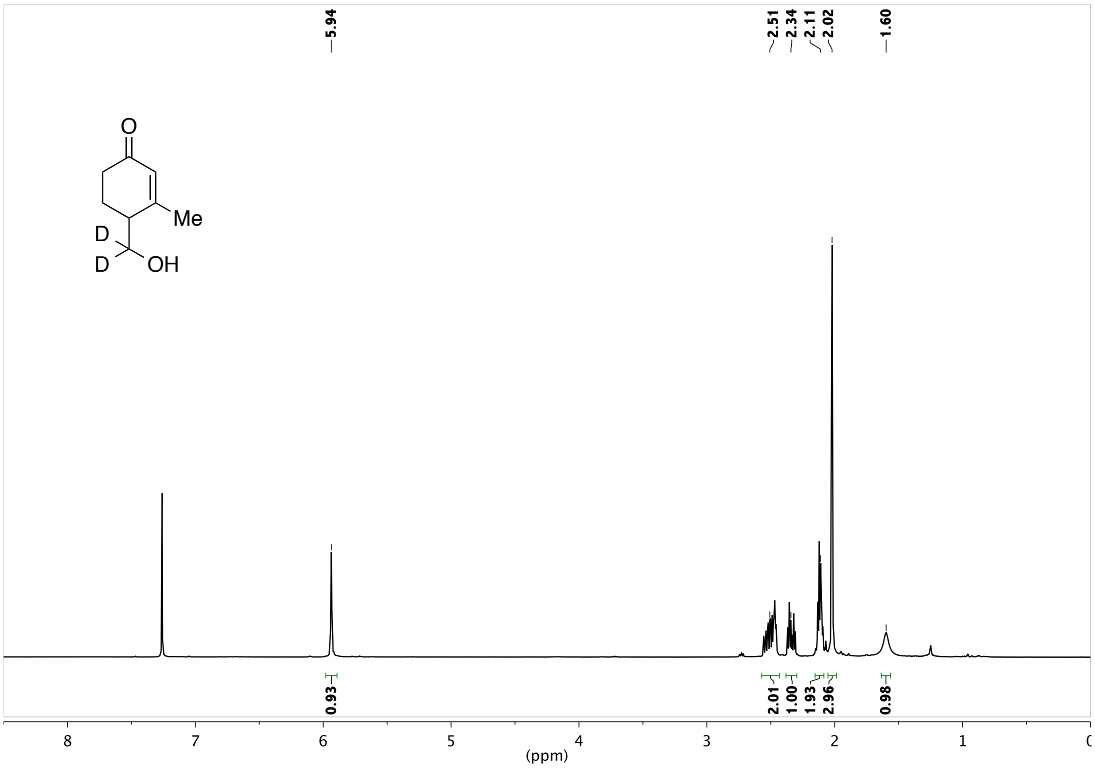

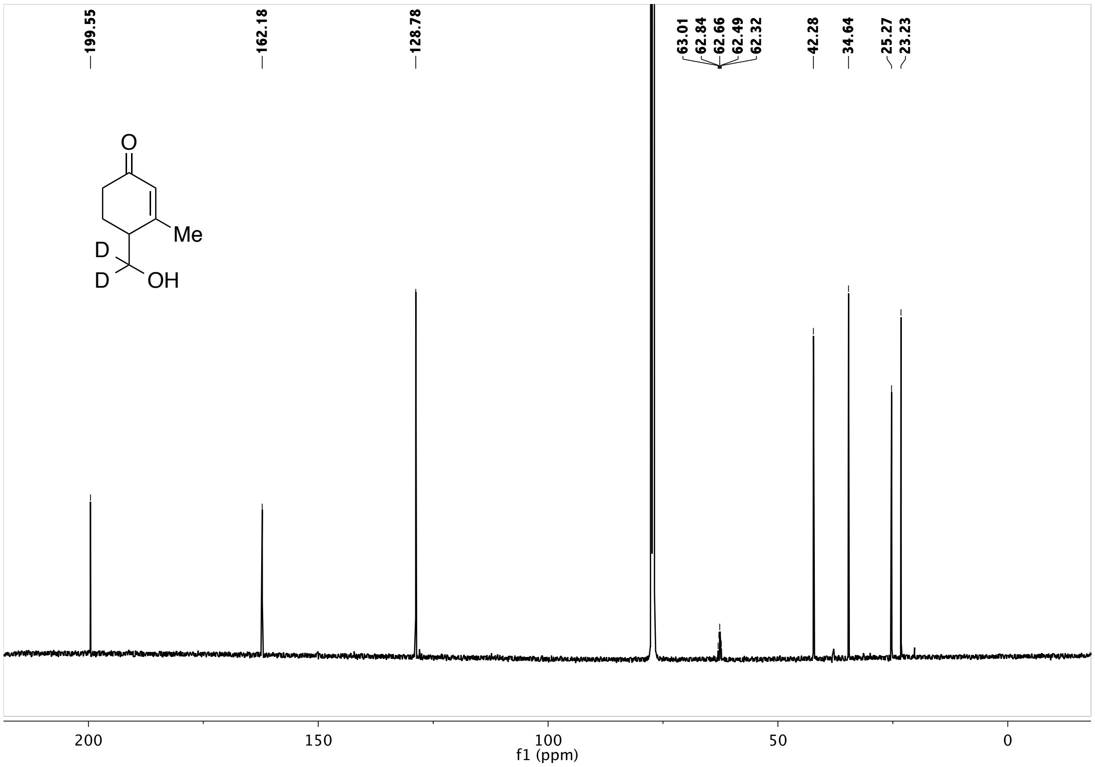

(2-Methyl-4-oxocyclohex-2-en-1-yl)(*d*2)methyl acetate (S1):

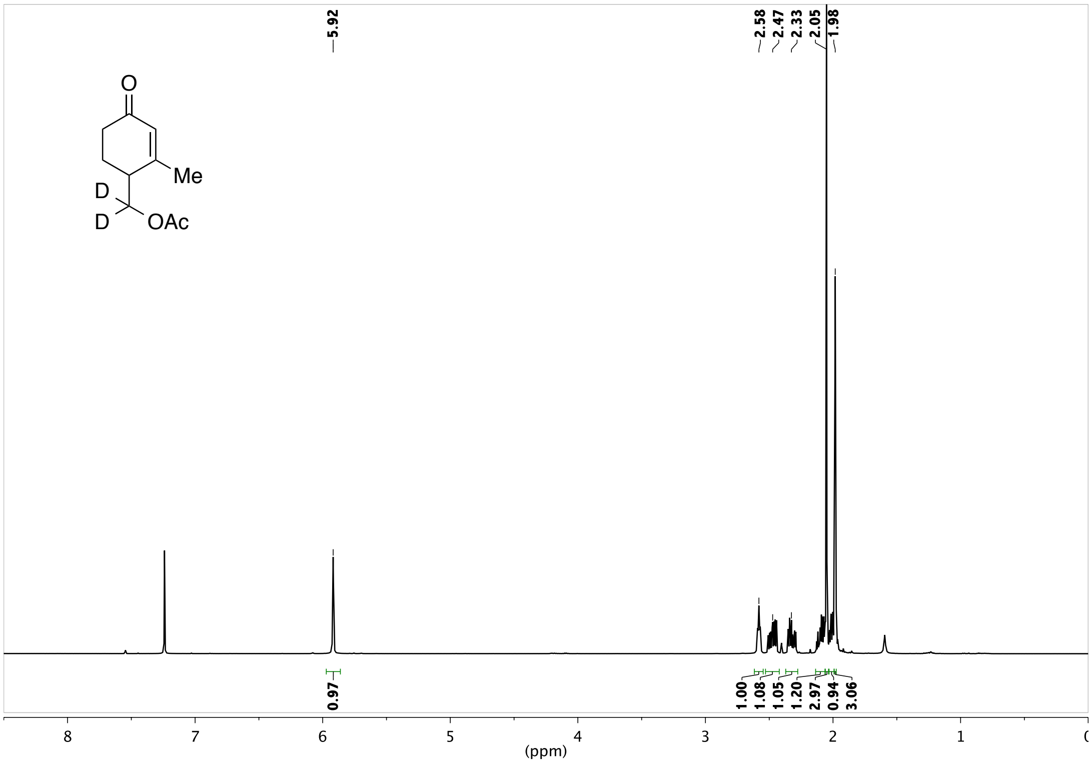

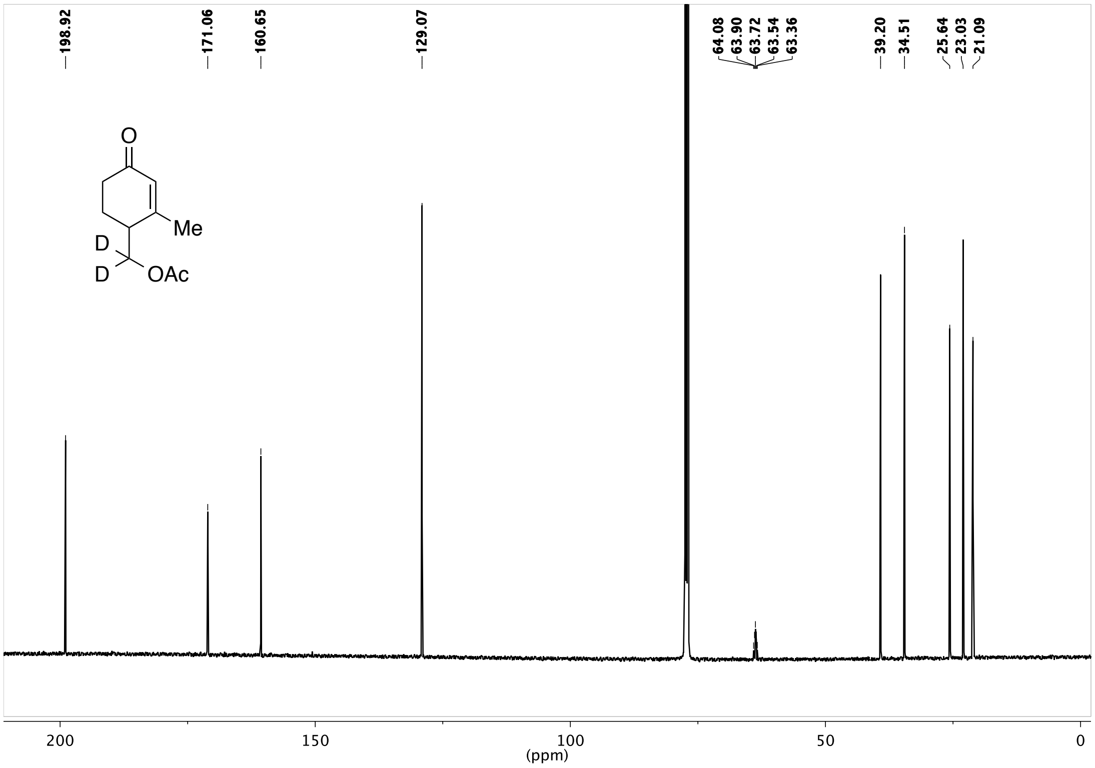

(2,2-Dimethyl-4-oxocyclohexyl)(*d*2)methyl acetate (11):

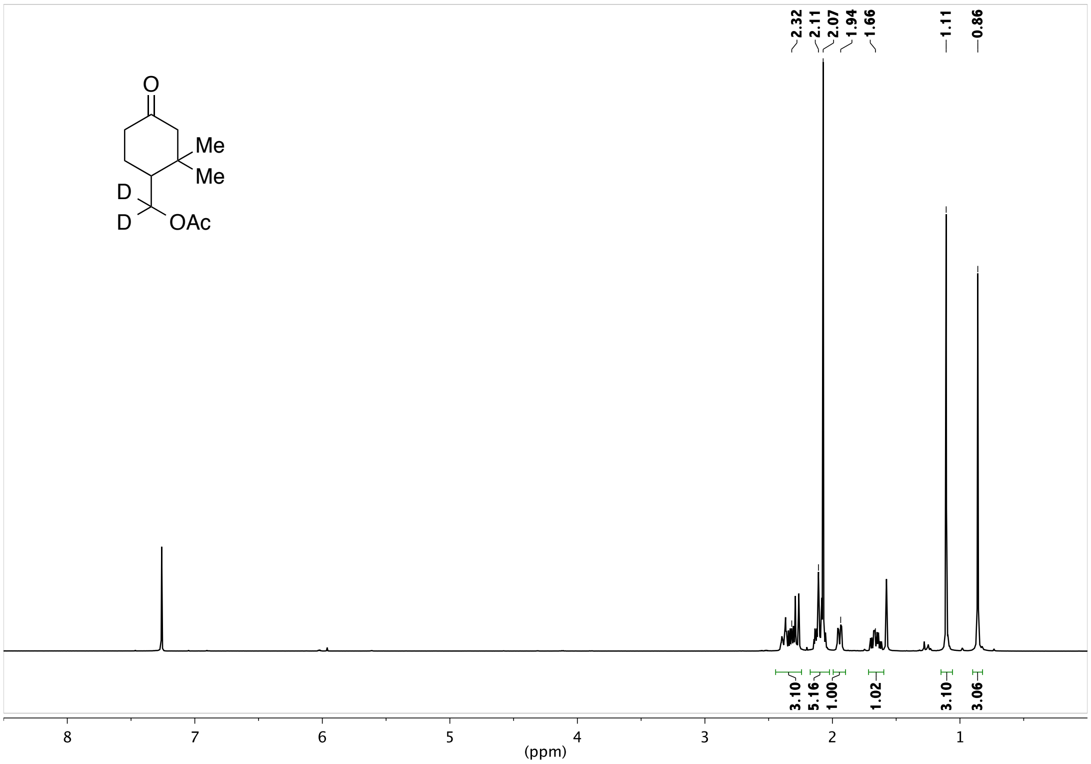

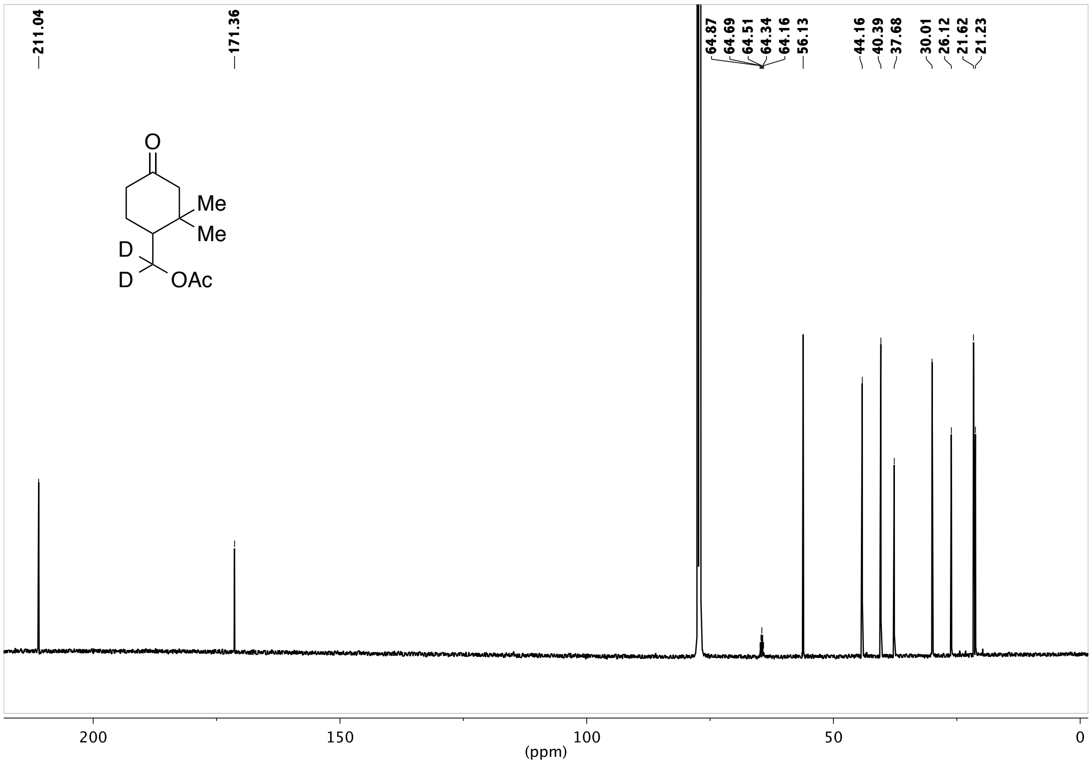

[(5*E*)-2,2-Dimethyl-4-oxo-5-(phenylmethylidene)cyclohexyl](*d*2)methyl 4-methylbenzene-1-sulfonate (12):

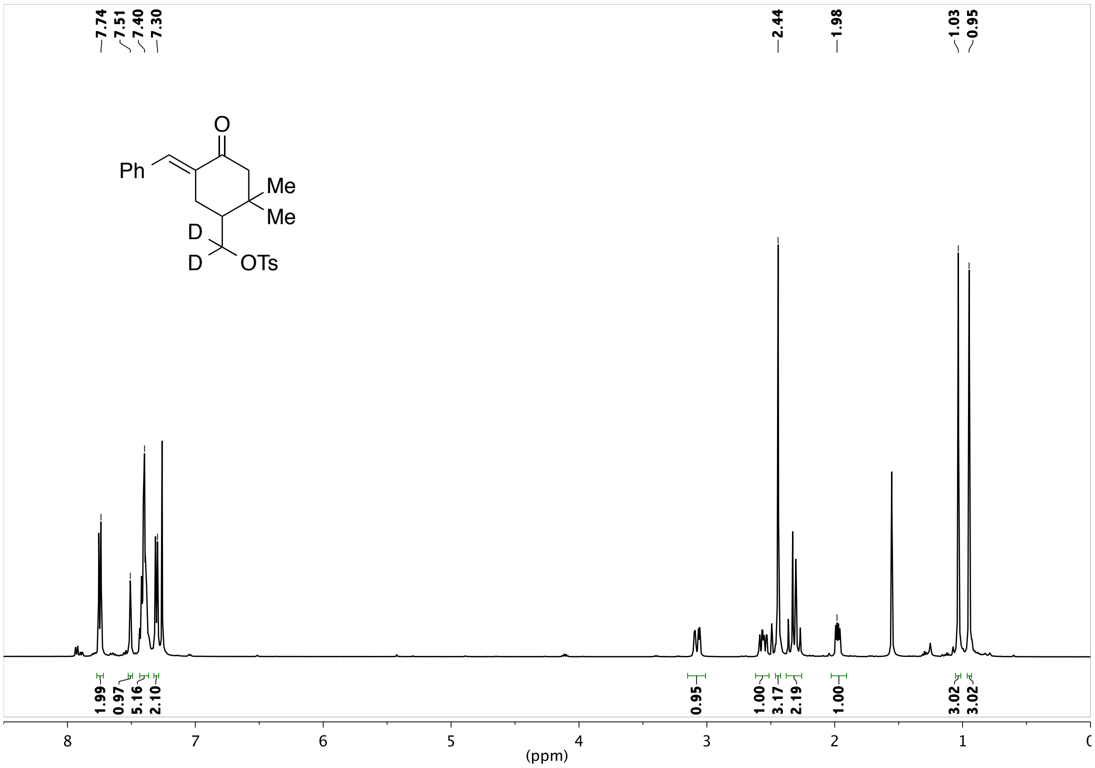

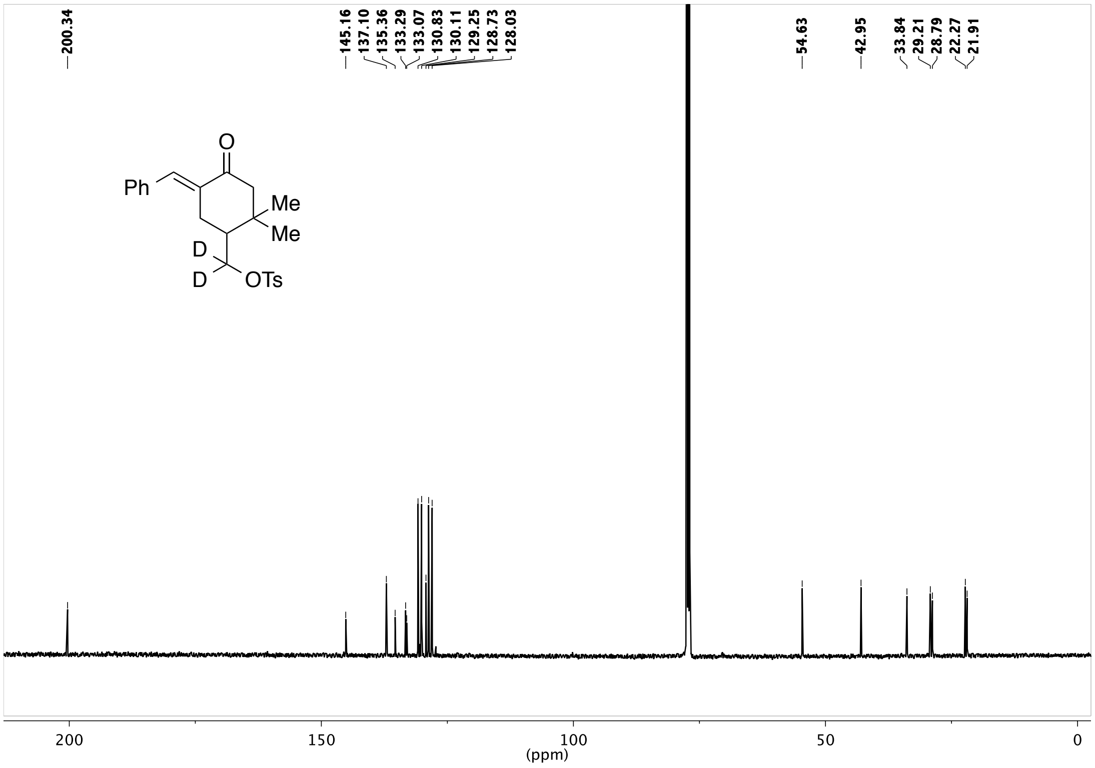

(3E)-6,6-Dimethyl-3-(phenylmethylidene) (7,7,-*d*2)bicyclo[3.1.1]heptan-2-one (13):

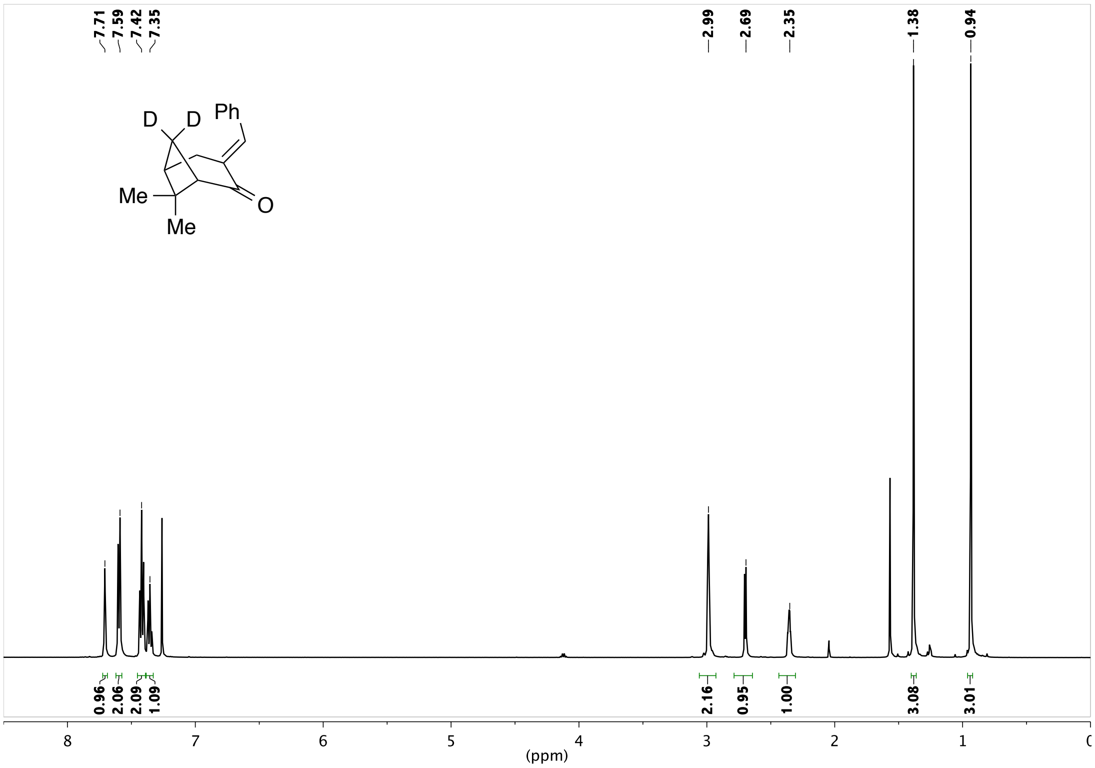

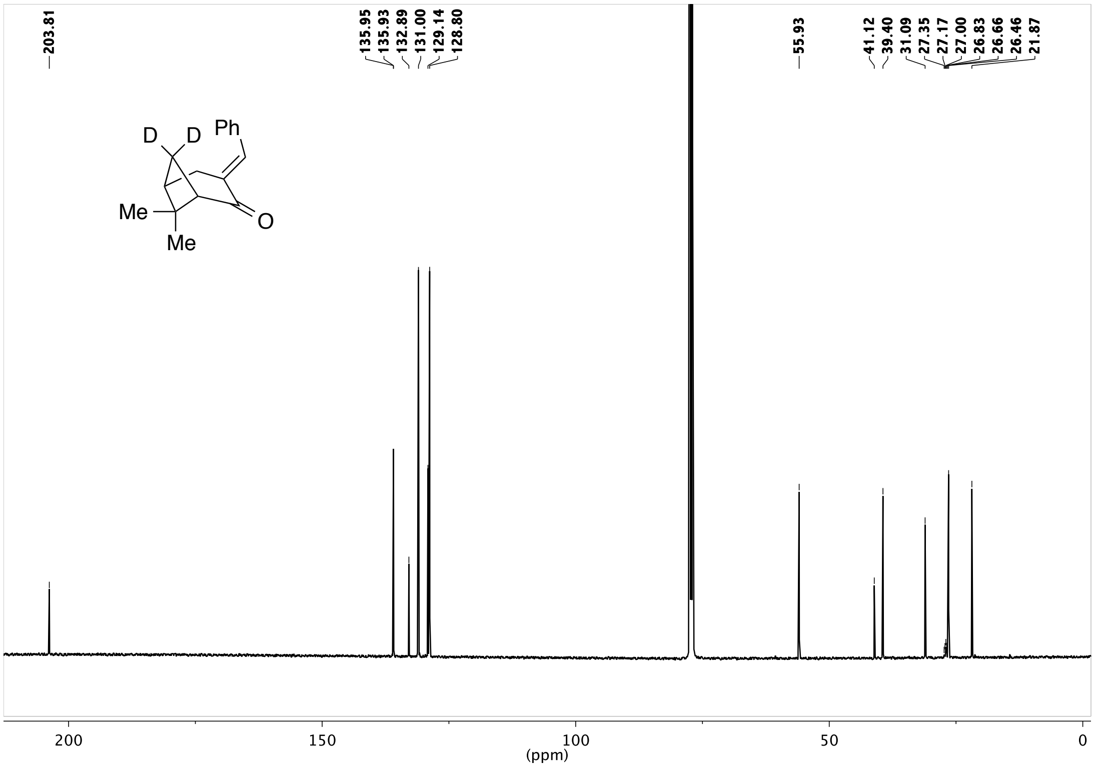

6,6-Dimethyl(7,7,-*d*2)bicyclo[3.1.1]heptan-2-one (nopinone-*d*2) (14):

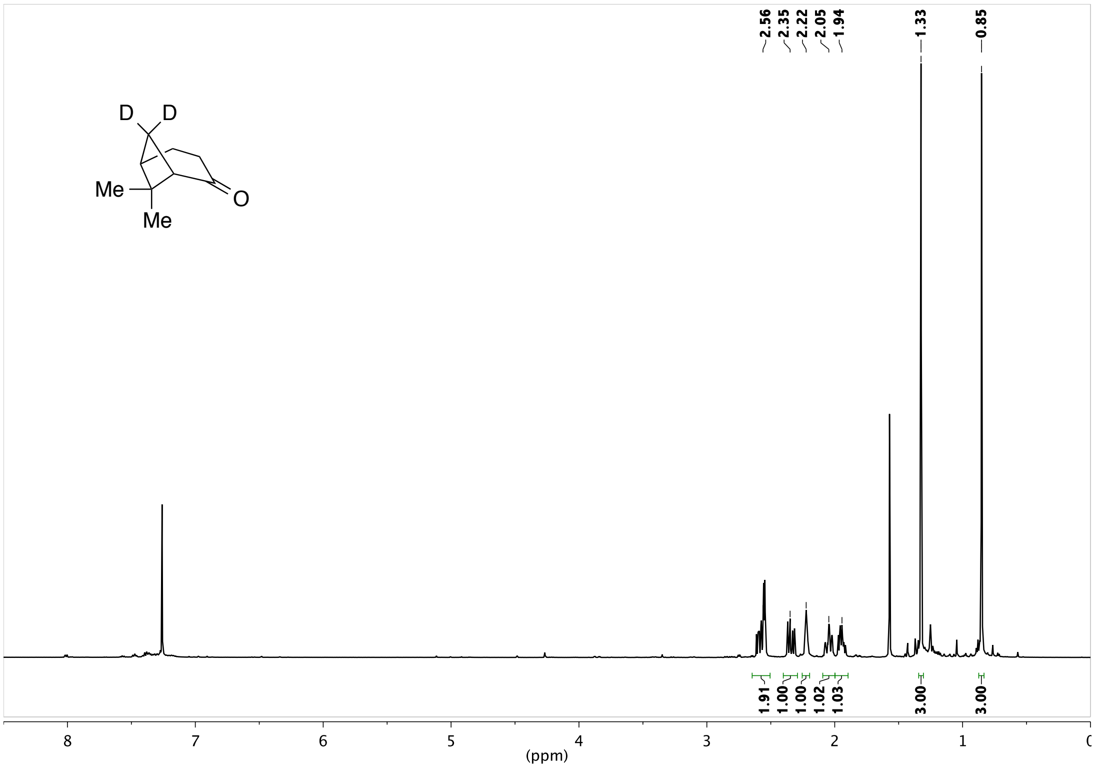

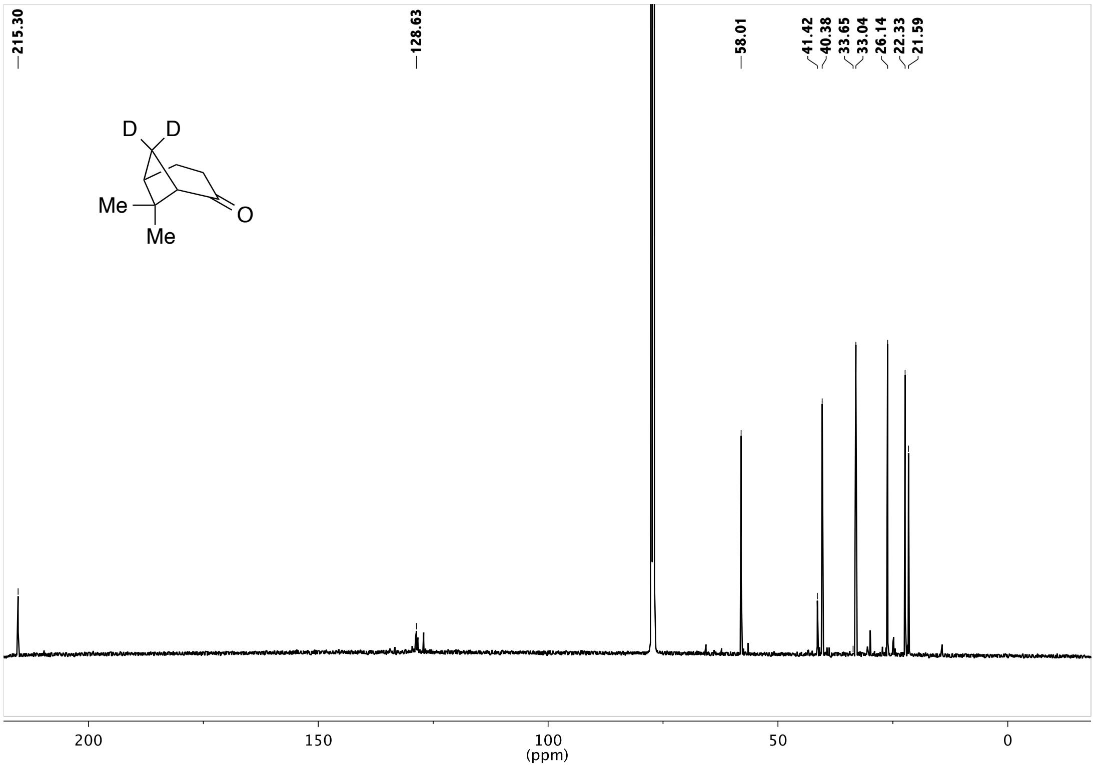

2,6,6-Trimethyl(7,7-*d*2)bicyclo[3.1.1]hept-2-ene ((±)-α-pinene-*d*2) (5):

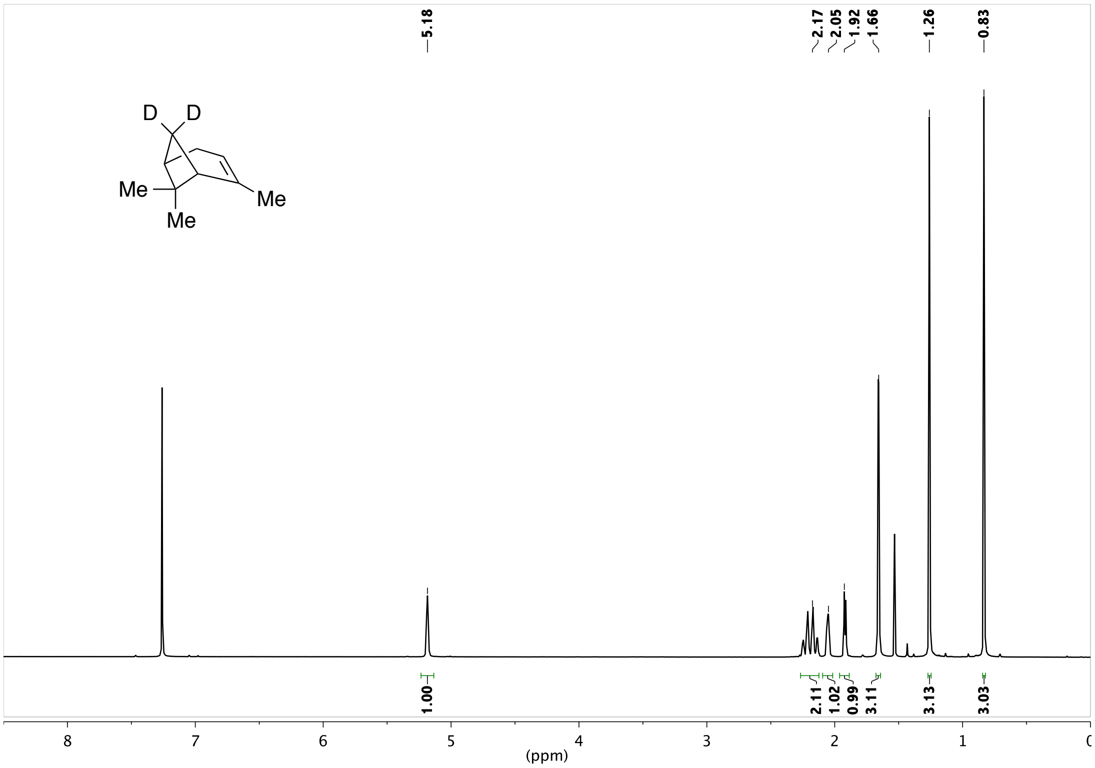

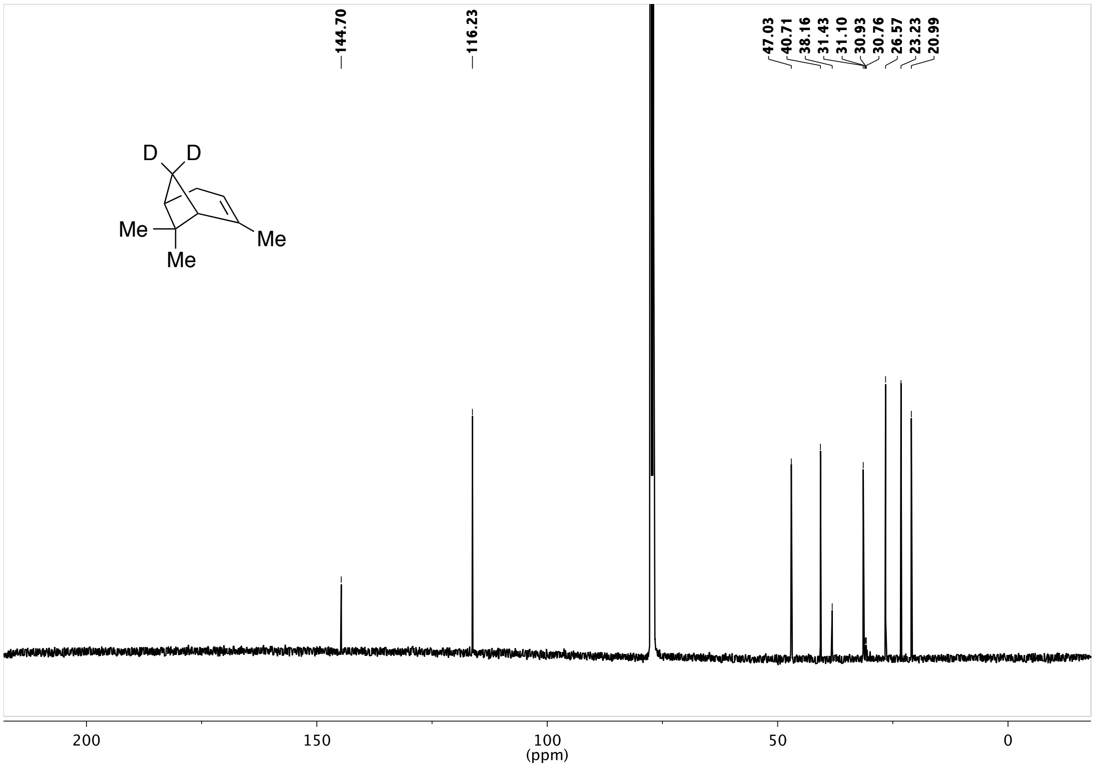

Ethyl 2-methyl-4-oxo(1,3-*d*2)cyclohex-2-ene-1-carboxylate (16):

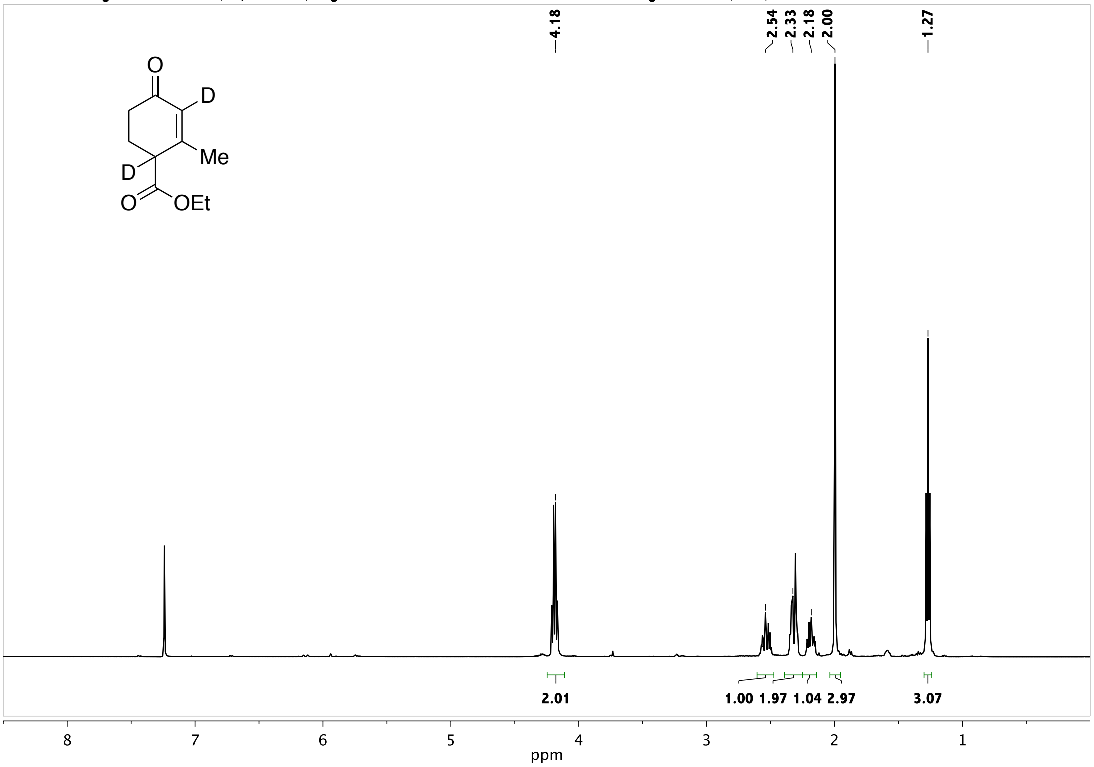

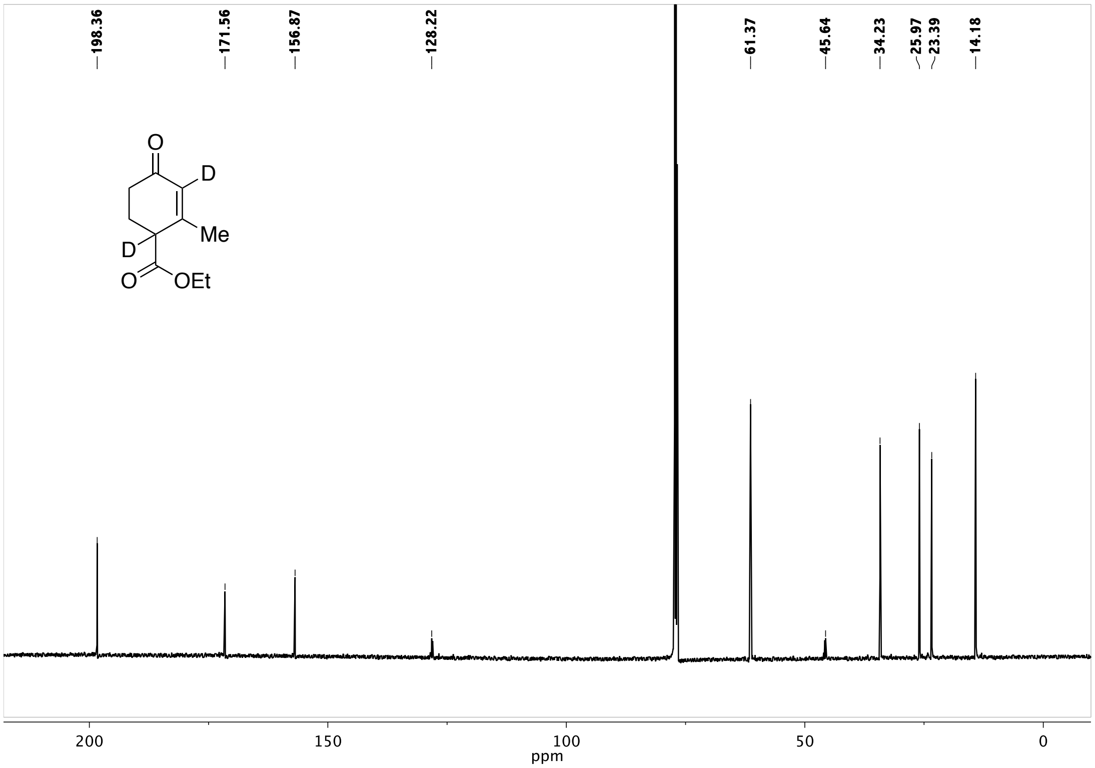

4-[Hydroxylmethyl]-3-methyl(1,2,4-*d*3)cyclohex-2-en-1-ol (S2):

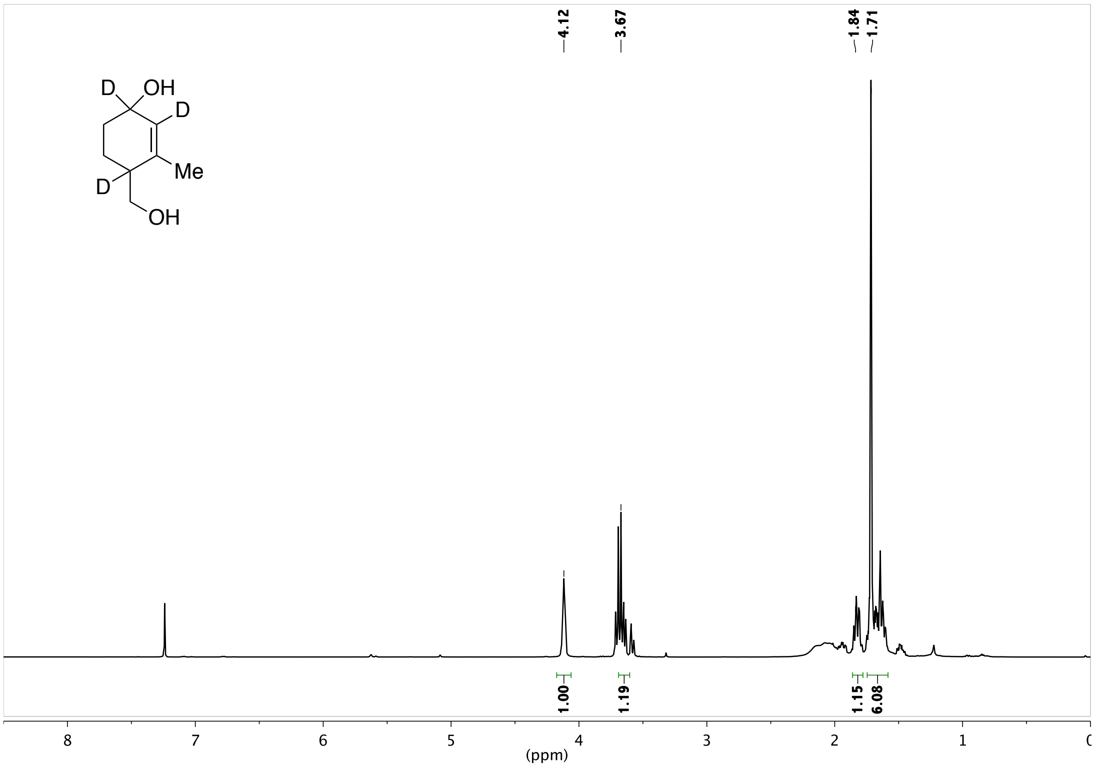

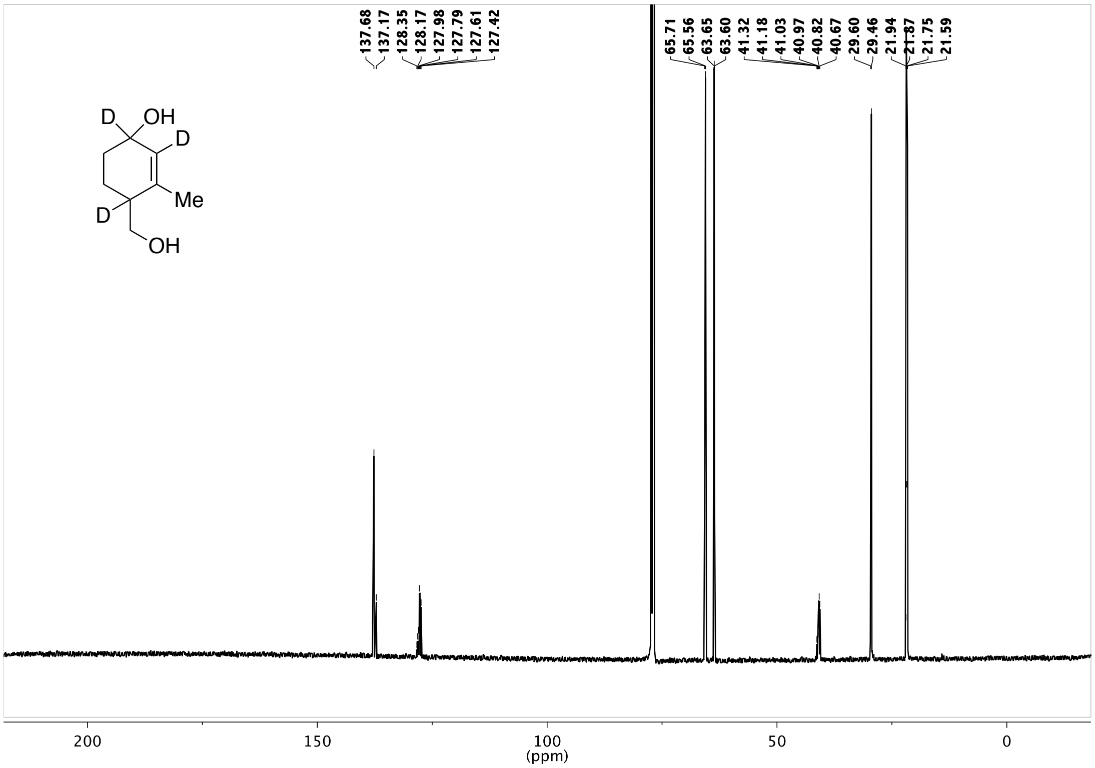

4-[Hydroxylmethyl]-3-methyl(2,4-*d*2)cyclohex-2-en-1-one (17):

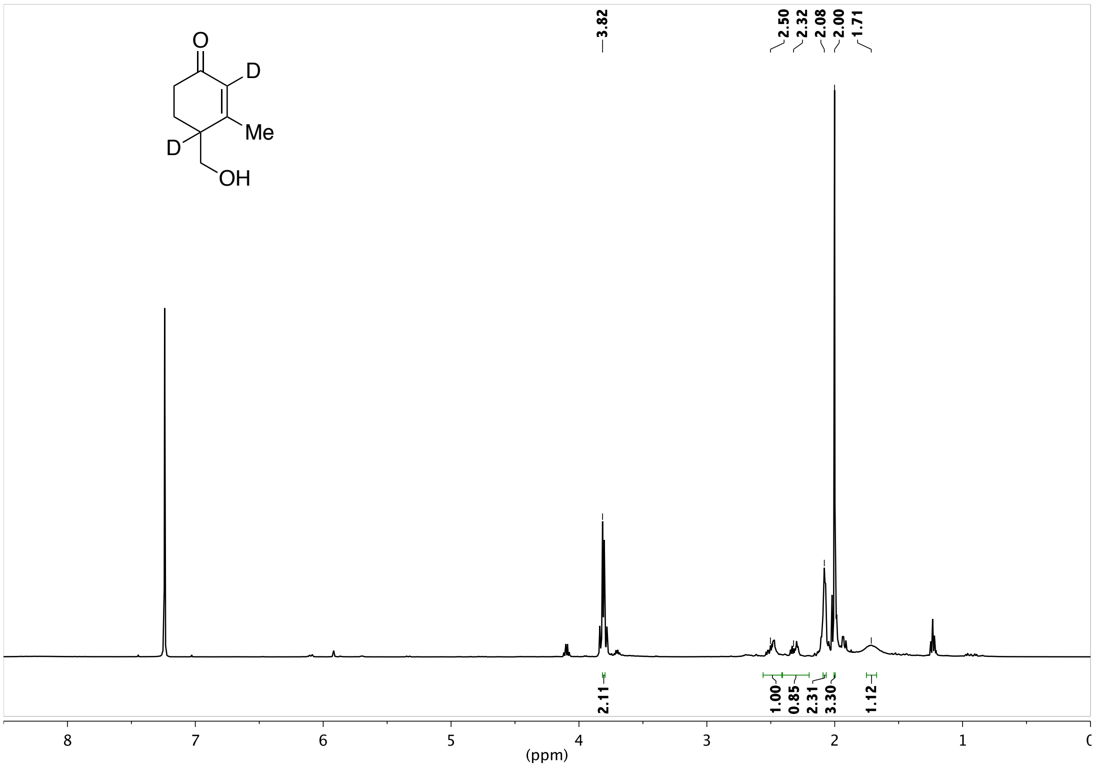

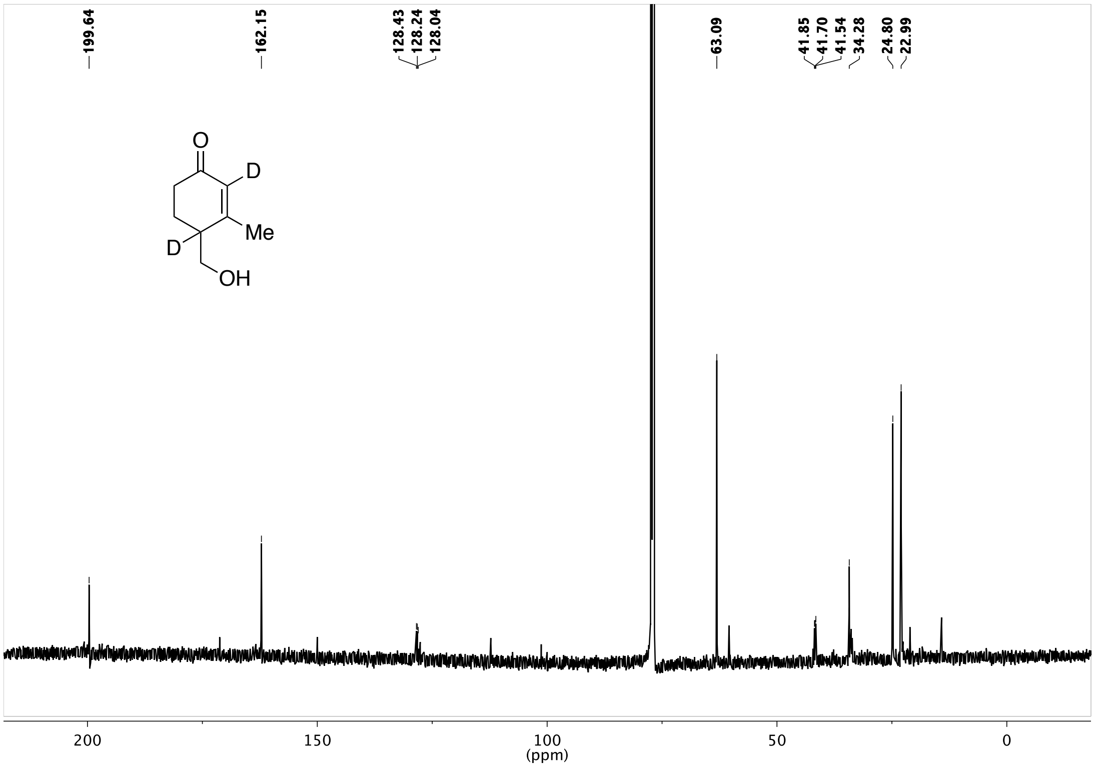

[2-Methyl-4-oxo(1,3-*d*2)cyclohex-2-en-1-yl]methyl acetate (S3):

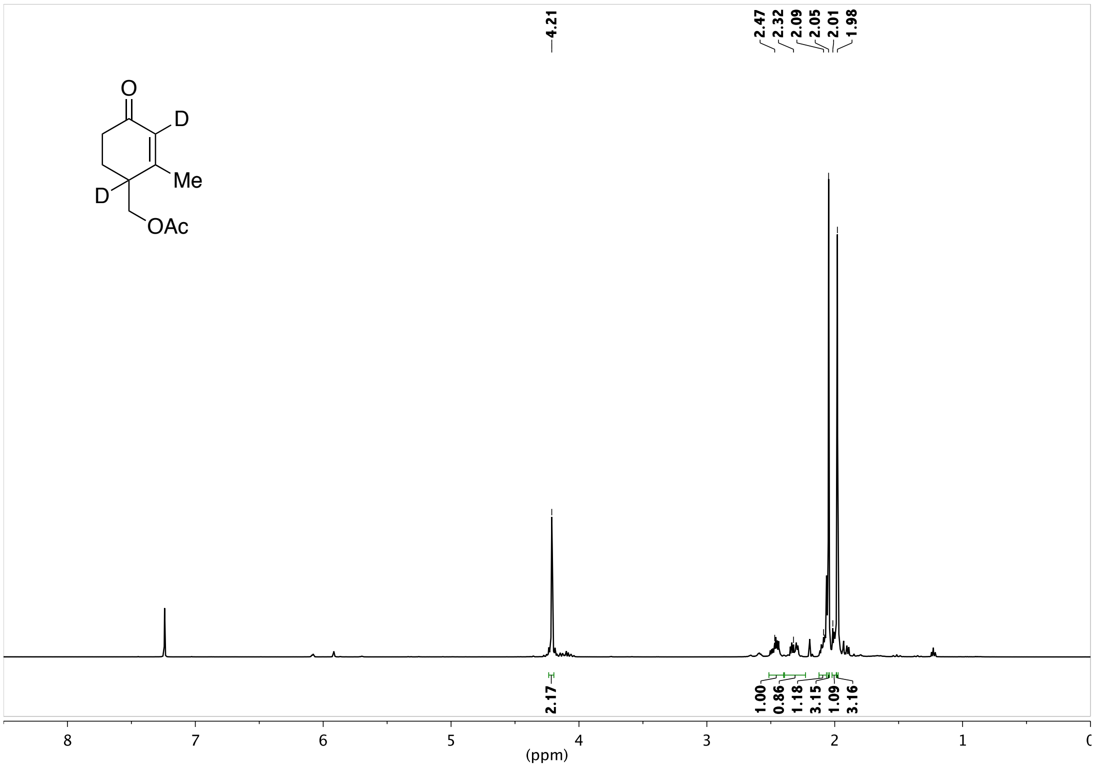

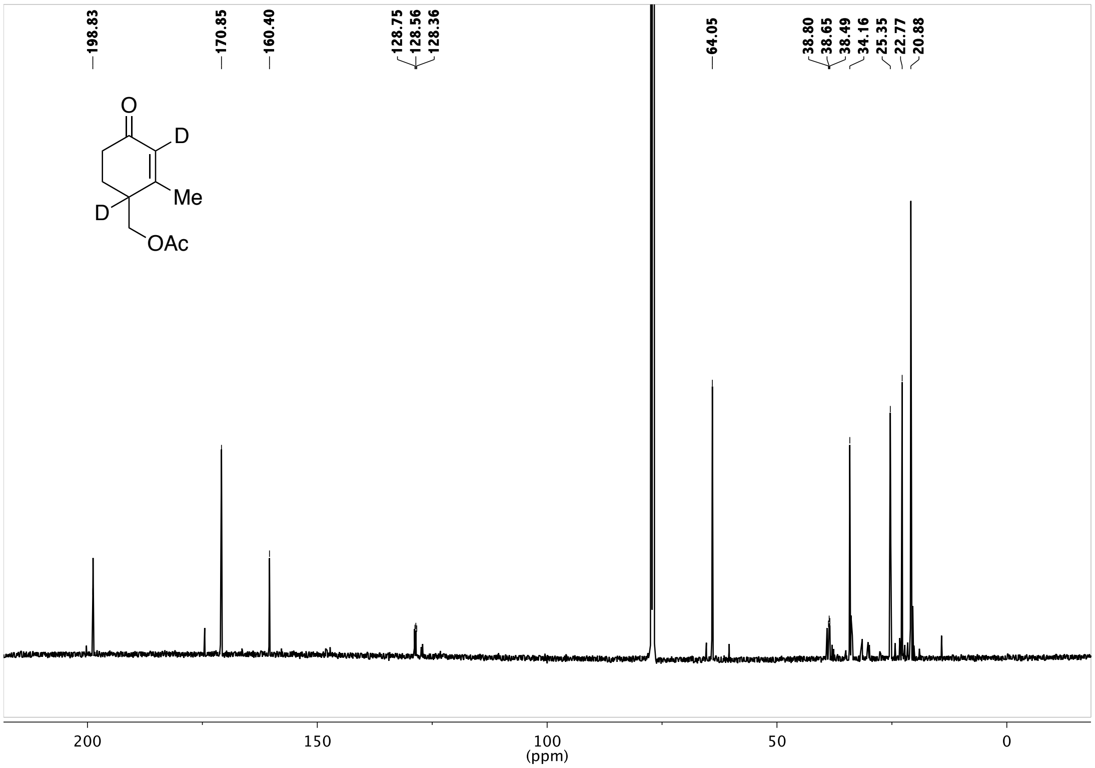

[2,2-Dimethyl-4-oxo(1,3-*d*2)cyclohexyl]methyl acetate (19):

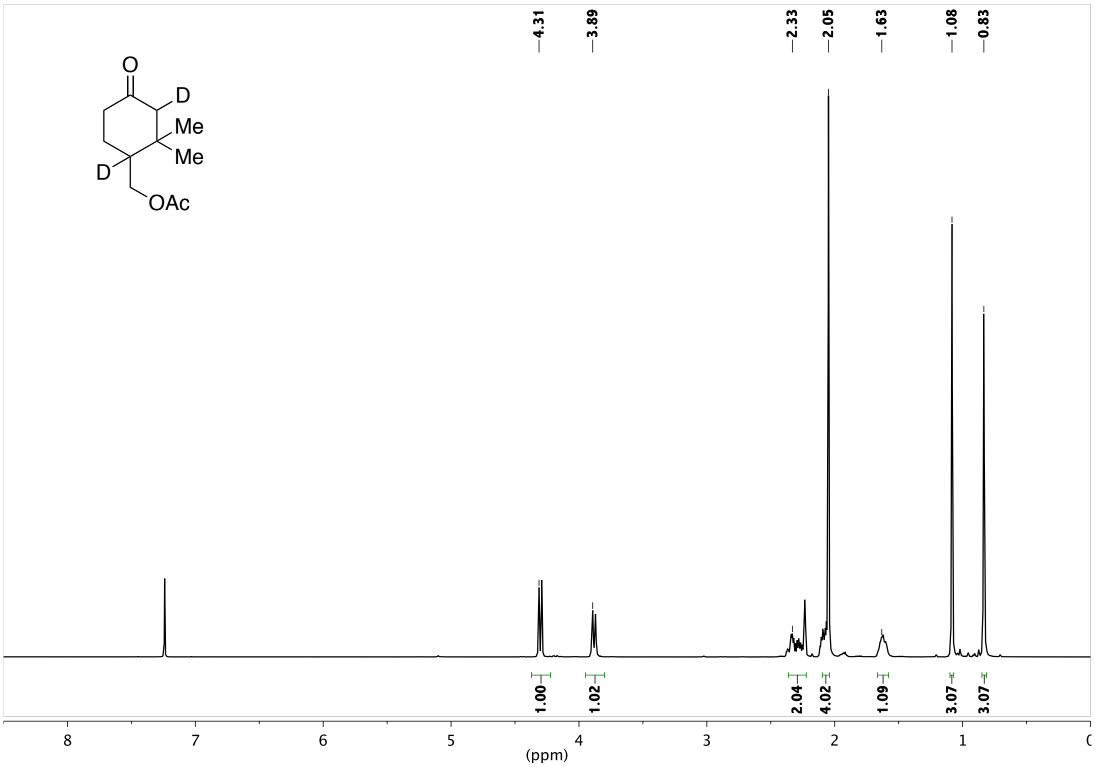

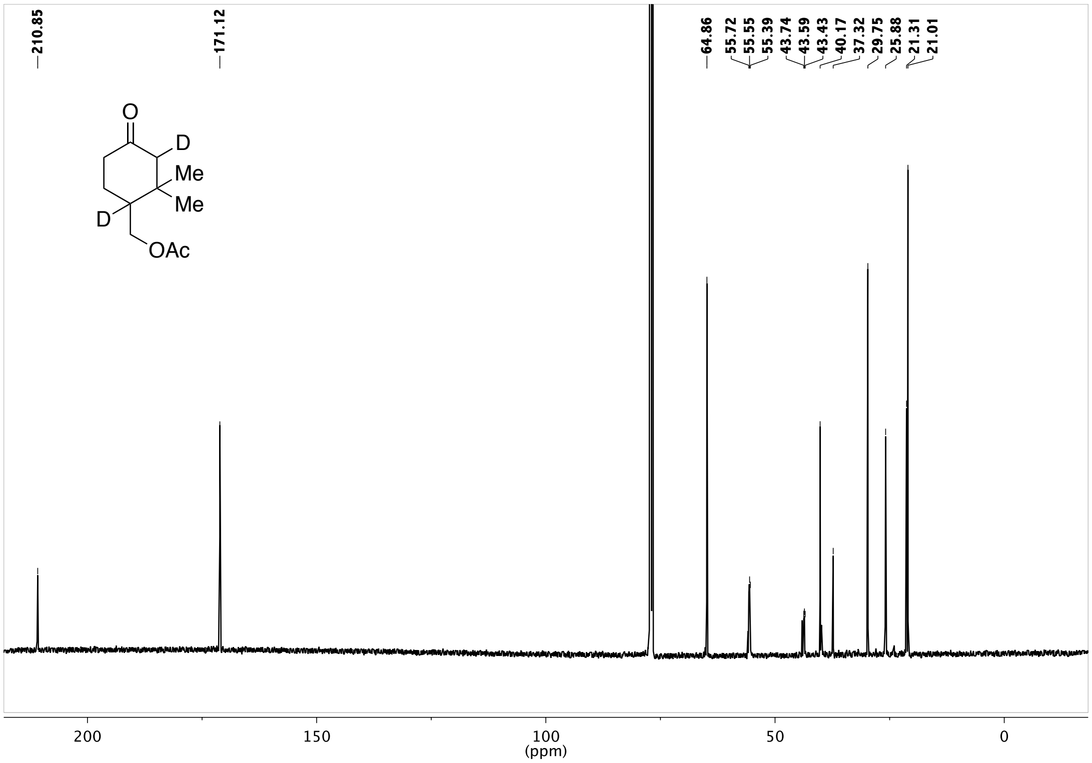

[(5*E*)-2,2-Dimethyl-4-oxo-5-(phenylmethylidene) (1,3,3-*d*3)cyclohexyl]methyl 4-methylbenzene-1-sulfonate (20):

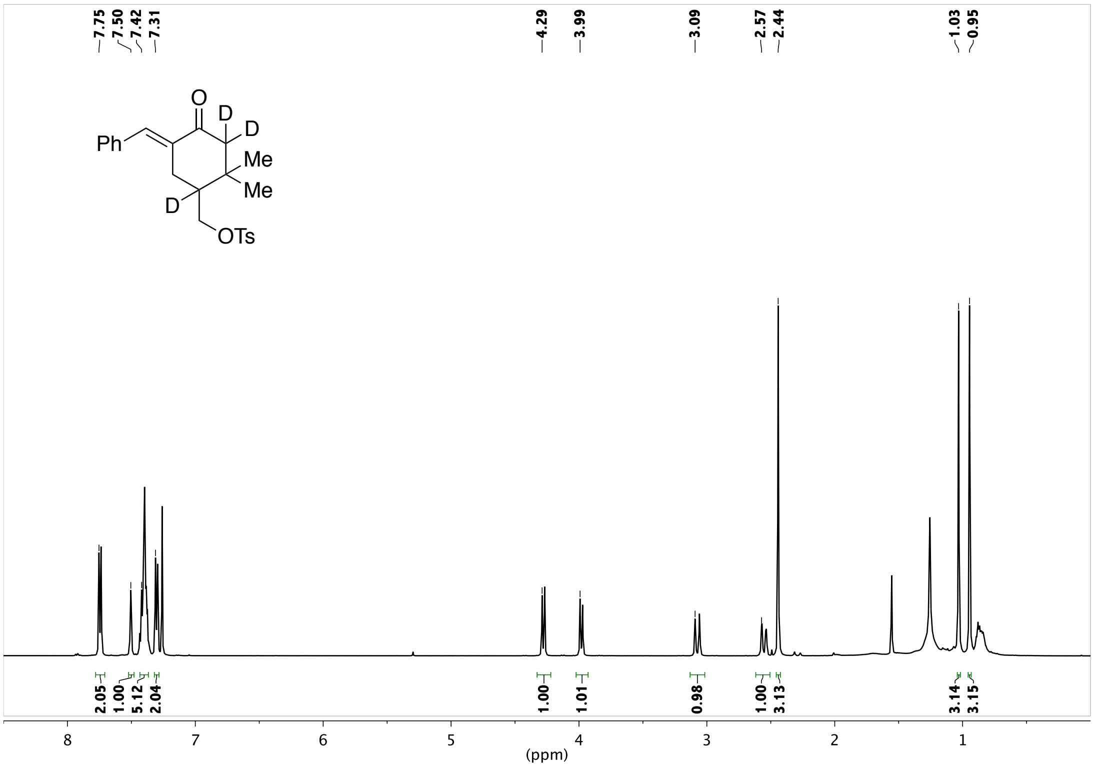

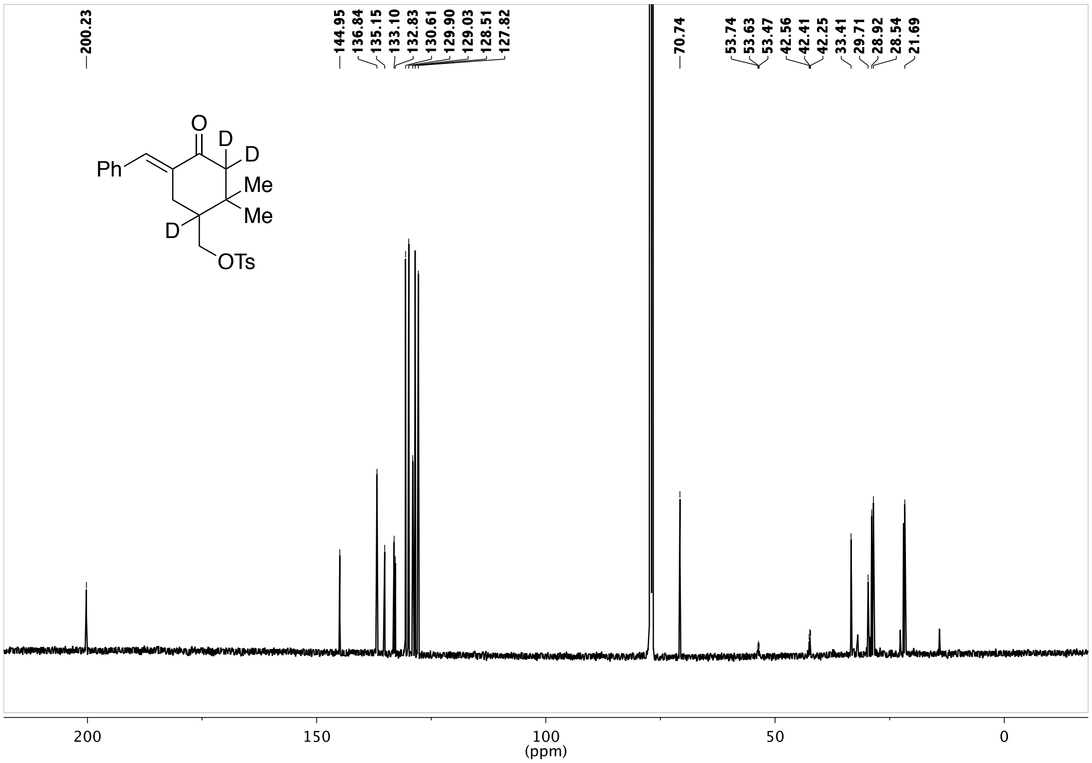

(3*E*)-6,6-Dimethyl-3-(phenylmethylidene) (1,5-*d*2)bicyclo[3.1.1]heptan-2-one (21):

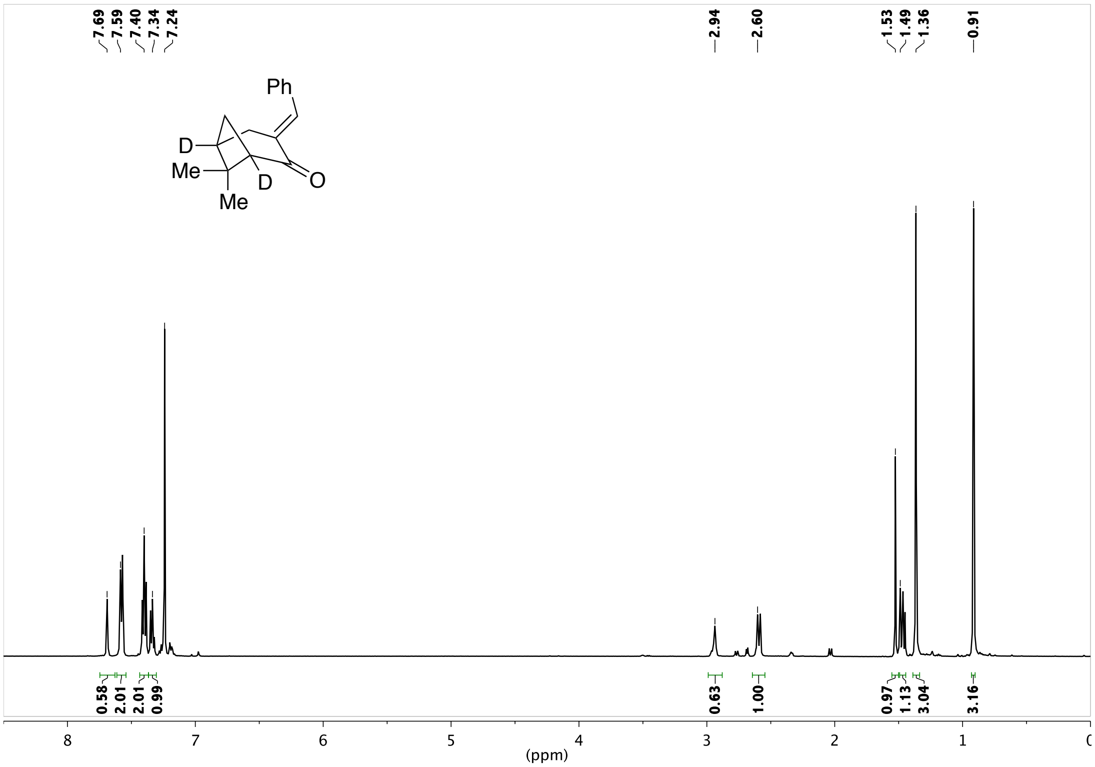

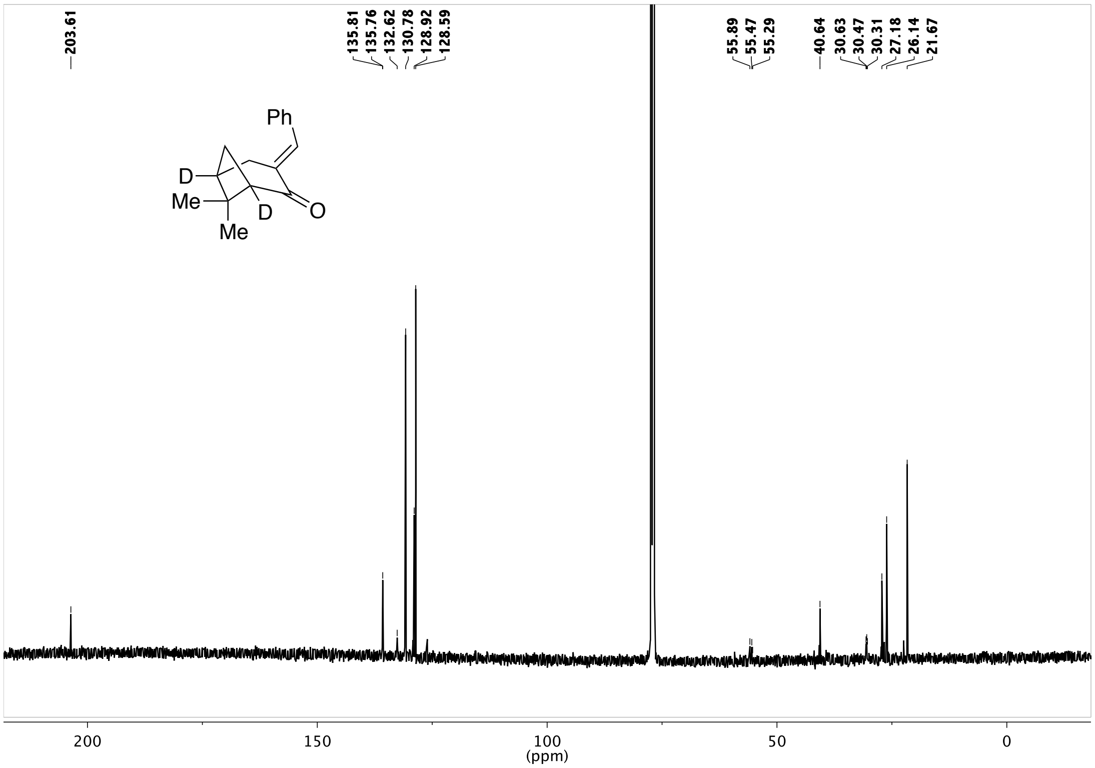

6,6-Dimethyl(1,3,3,4,5-*d*5)bicyclo[3.1.1]heptan-2-one (nopinone-*d*6) (22):

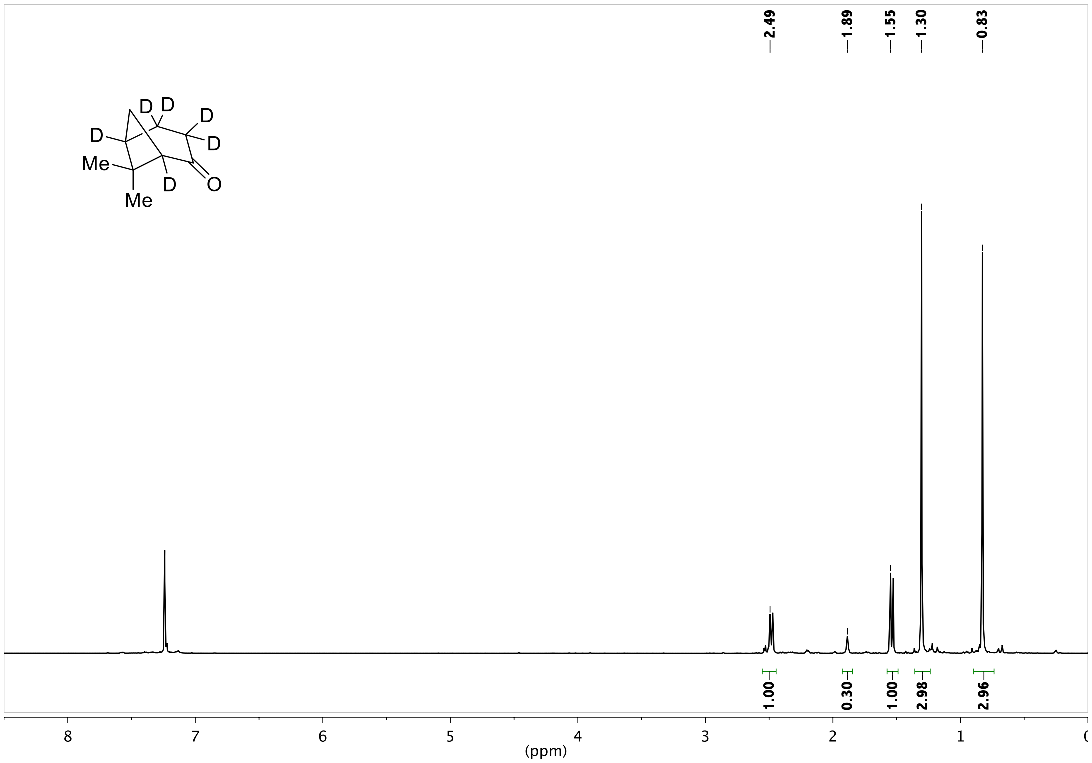

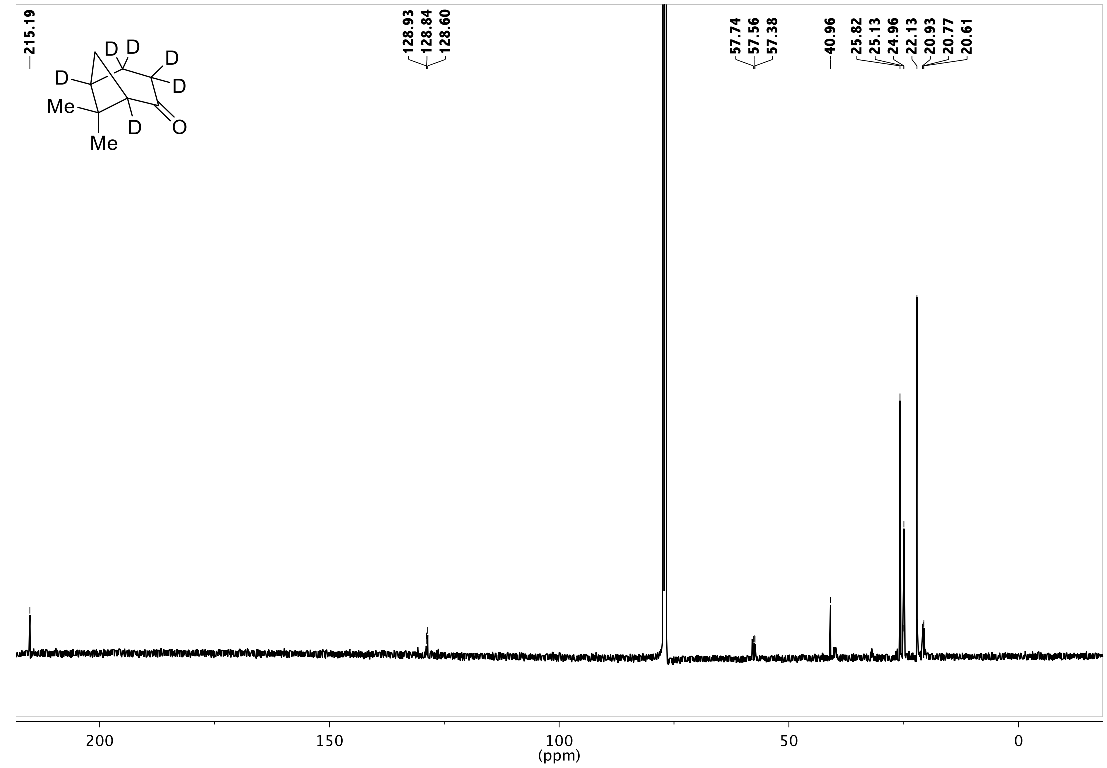

2,6,6-Trimethyl(1,3,4,5-*d*4)bicyclo[3.1.1]hept-2-ene ((±)-α-pinene-*d*5) (6):

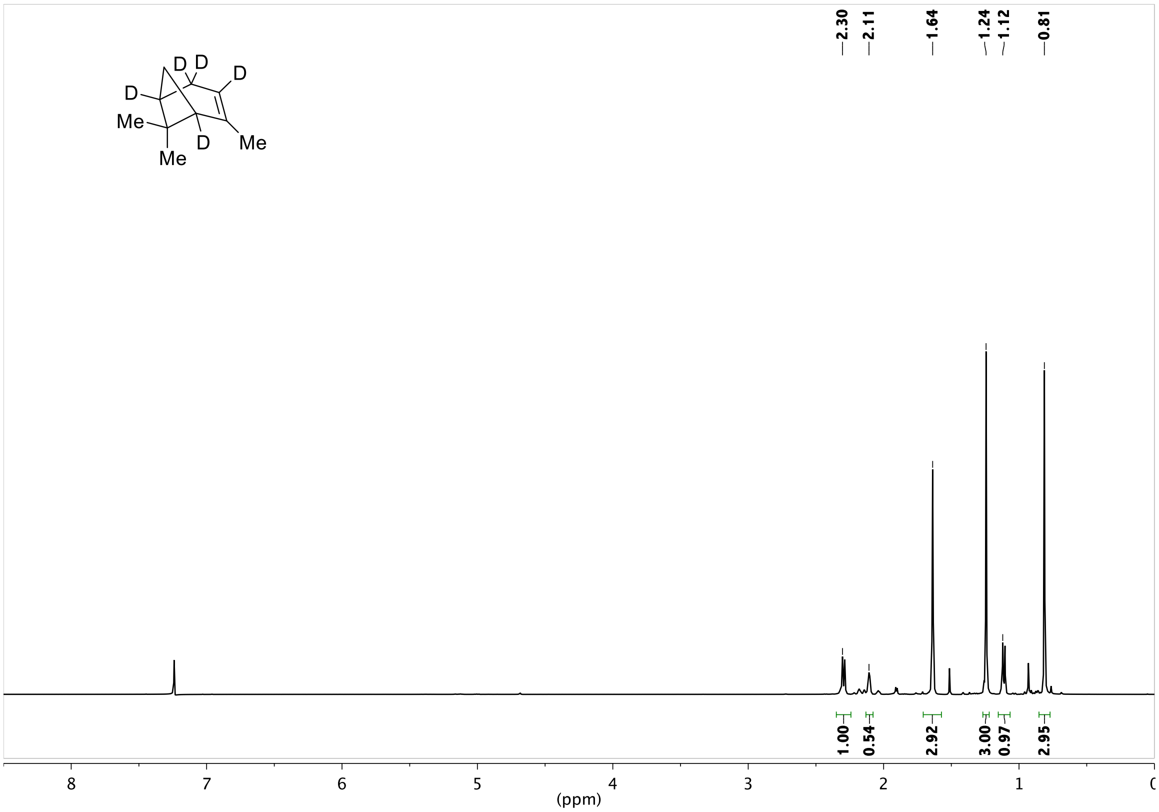

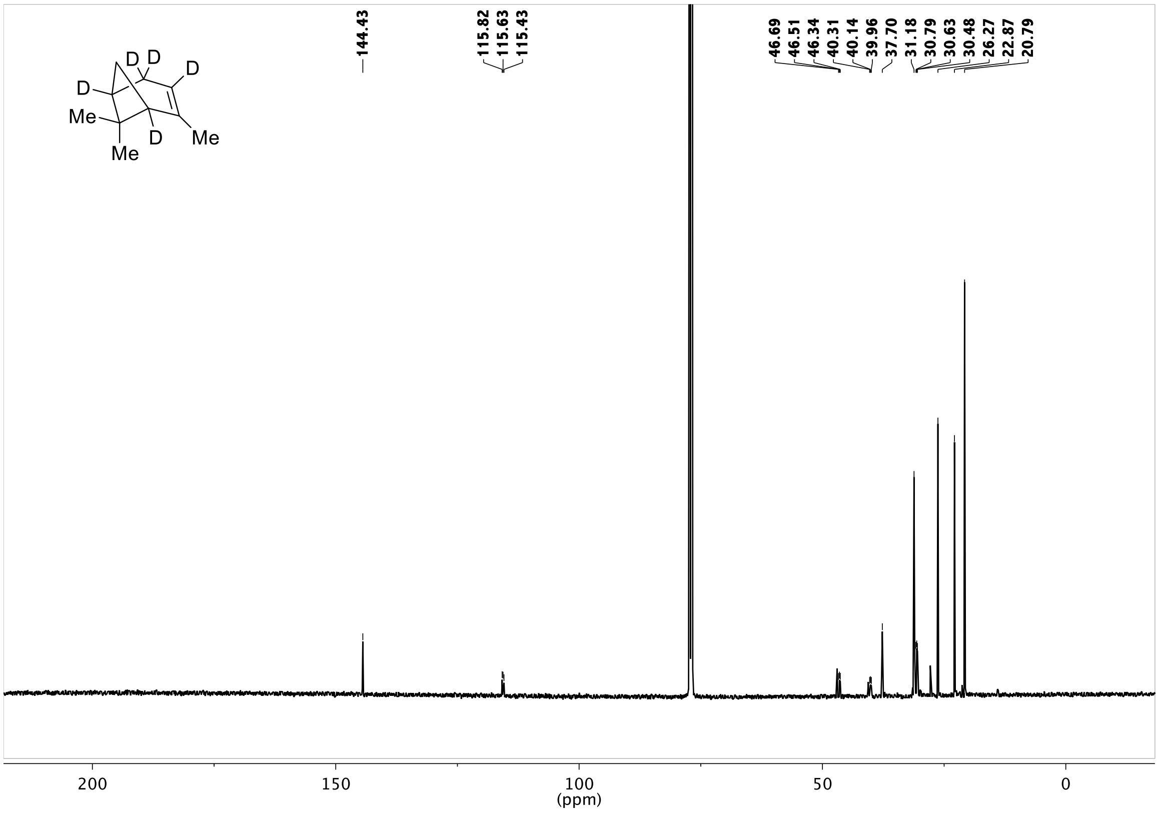

4-[Hydroxyl(*d*2)methyl]-3-methyl(1,2,4-*d*3)cyclohex-2-en-1-ol (S4):

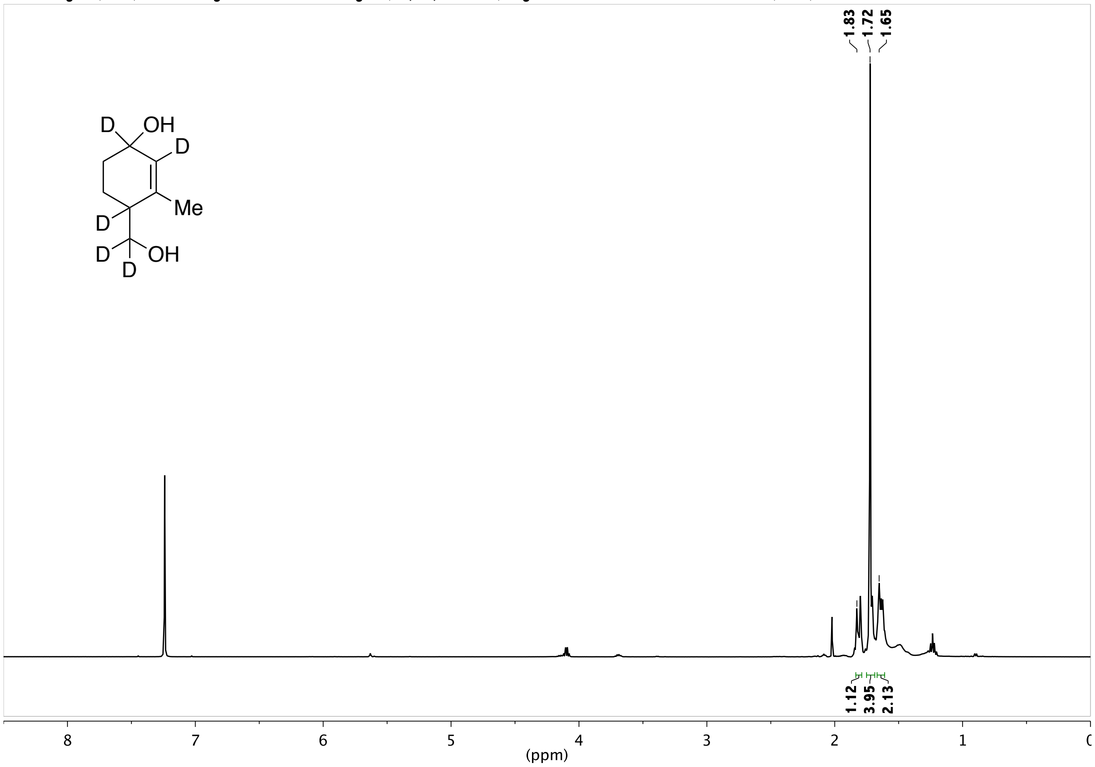

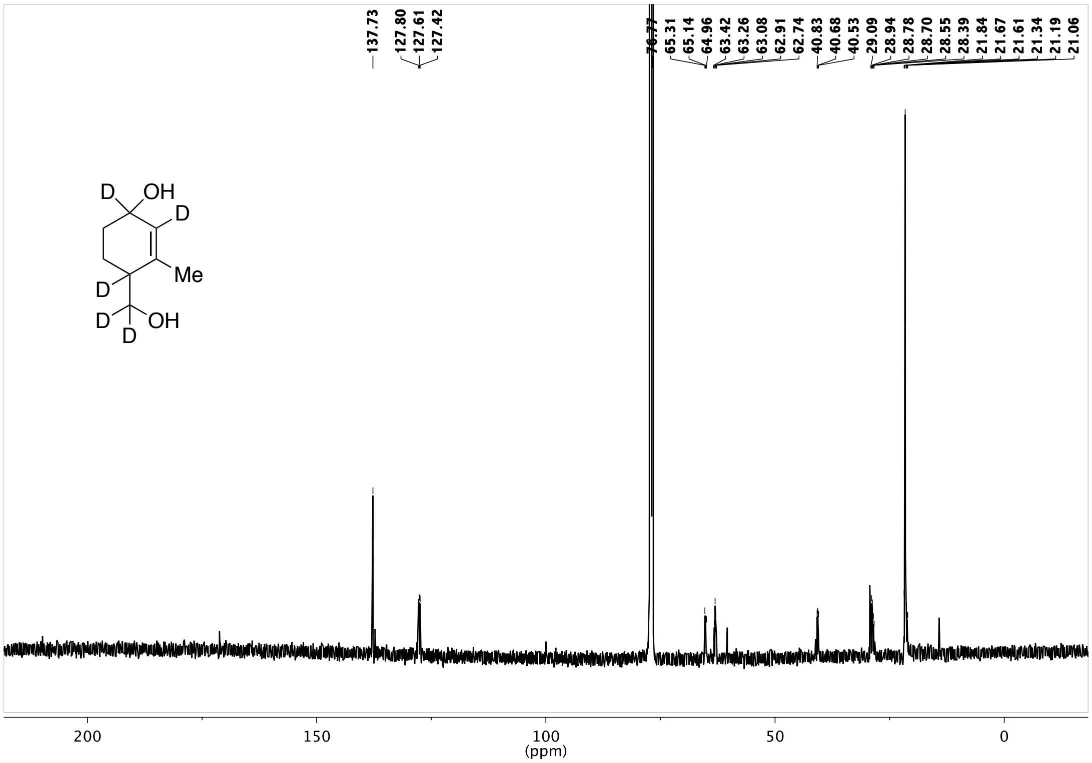

4-[Hydroxyl(*d*2)methyl]-3-methyl(2,4-*d*2)cyclohex-2-en-1-one (18):

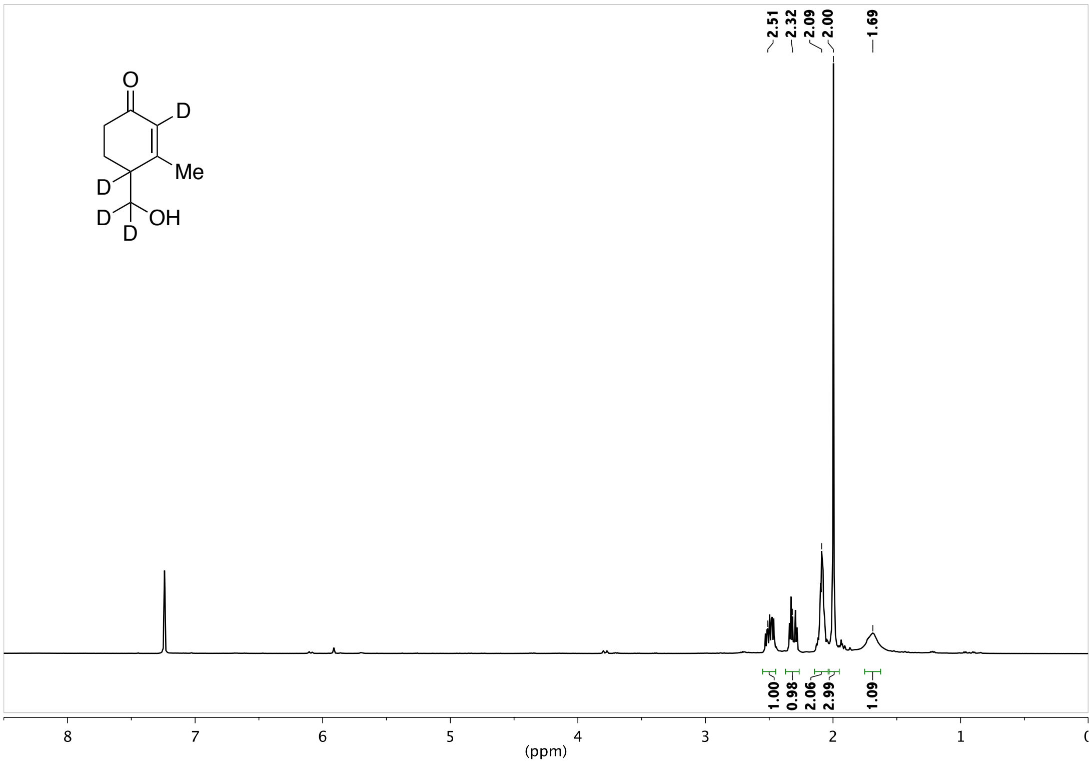

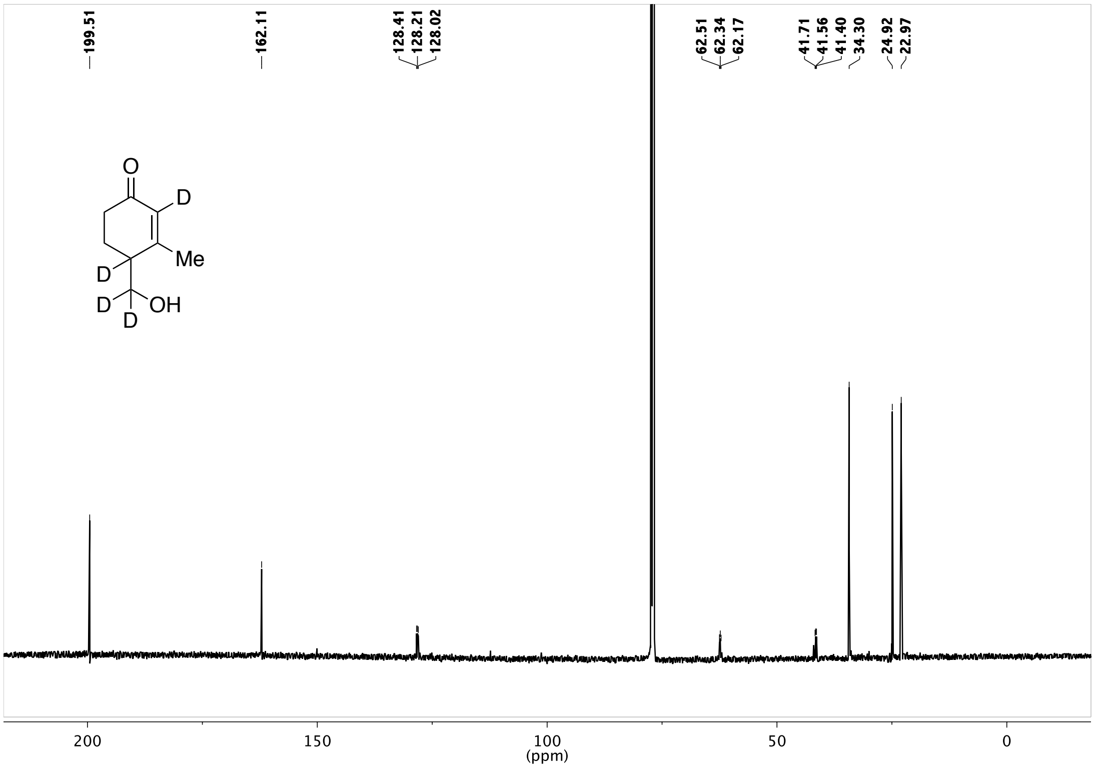

[2-Methyl-4-oxo(1,3-*d*2)cyclohex-2-en-1-yl](*d*2)methyl acetate (S5):

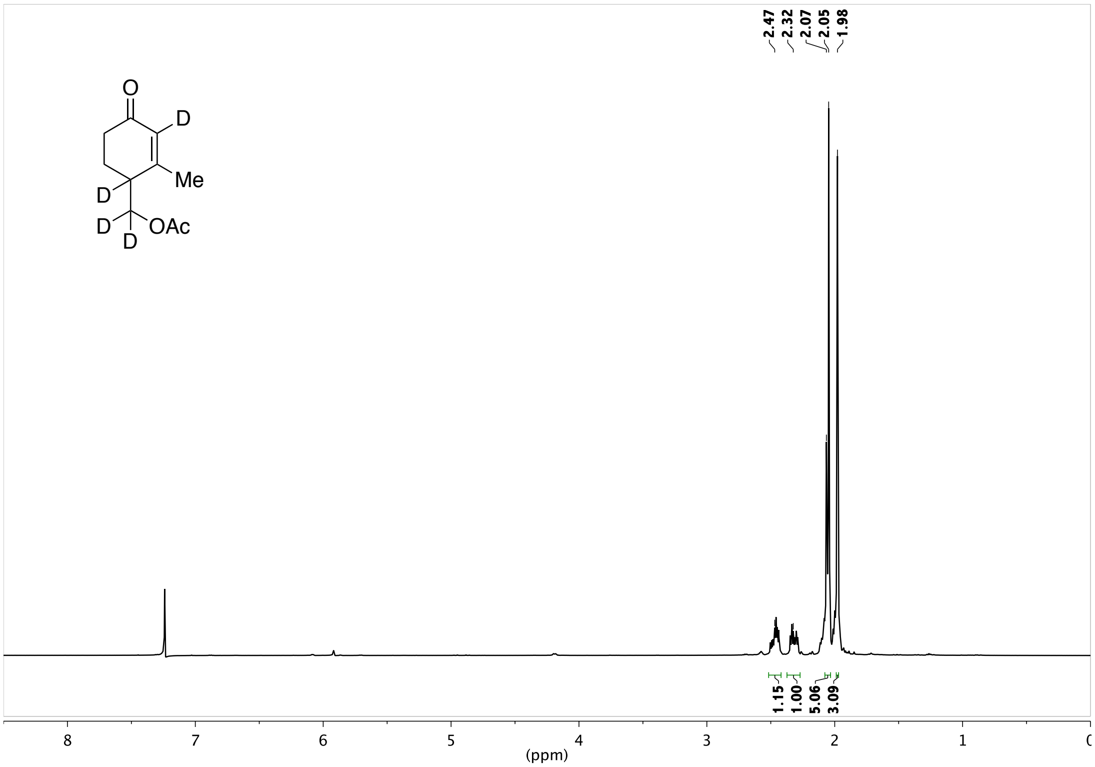

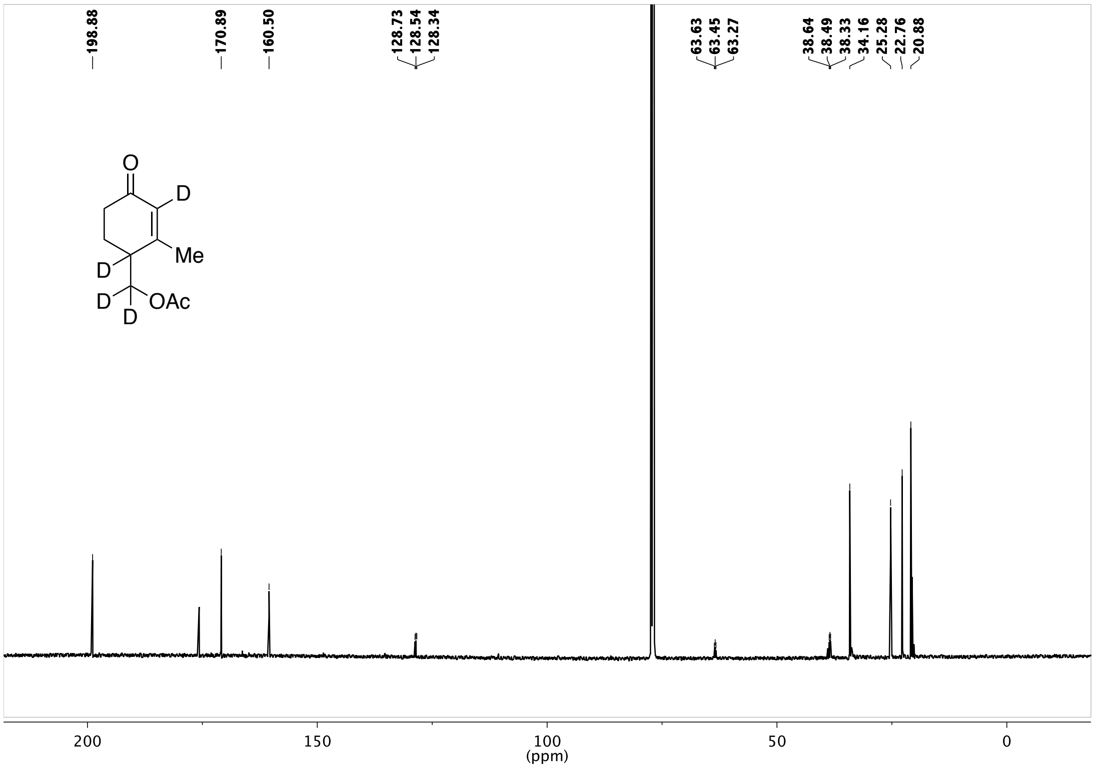

[2,2-Dimethyl-4-oxo(1,3-*d*2)cyclohexyl]methyl acetate (19):

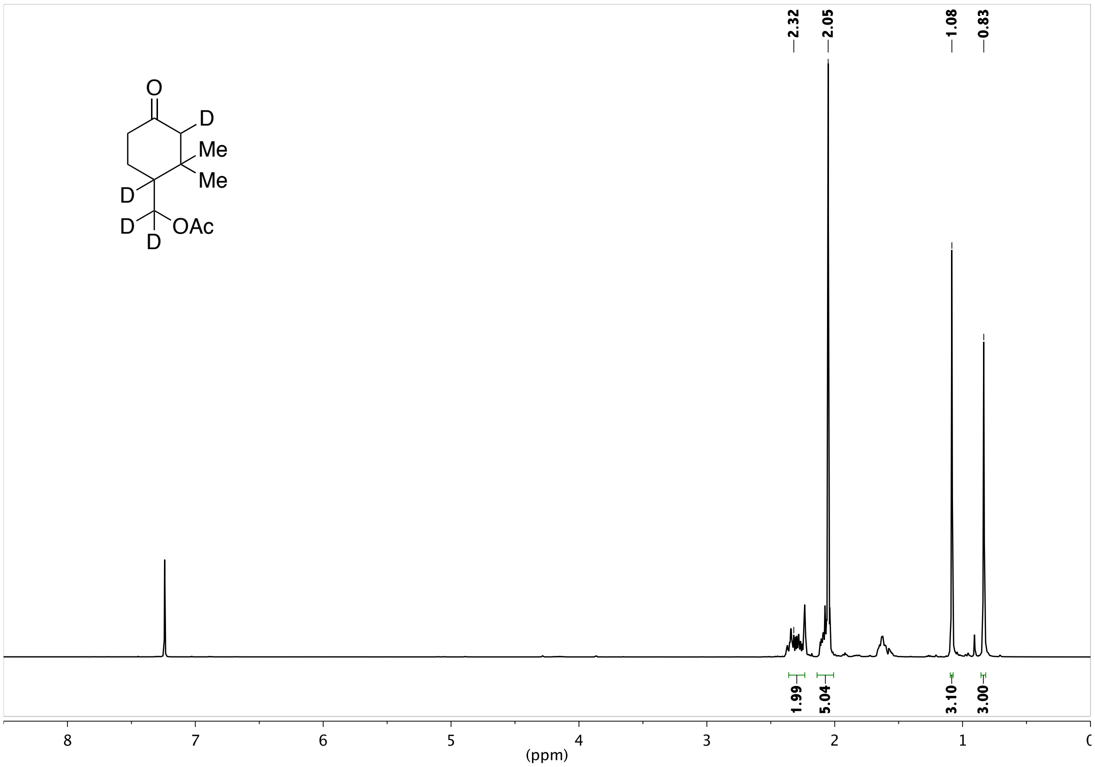

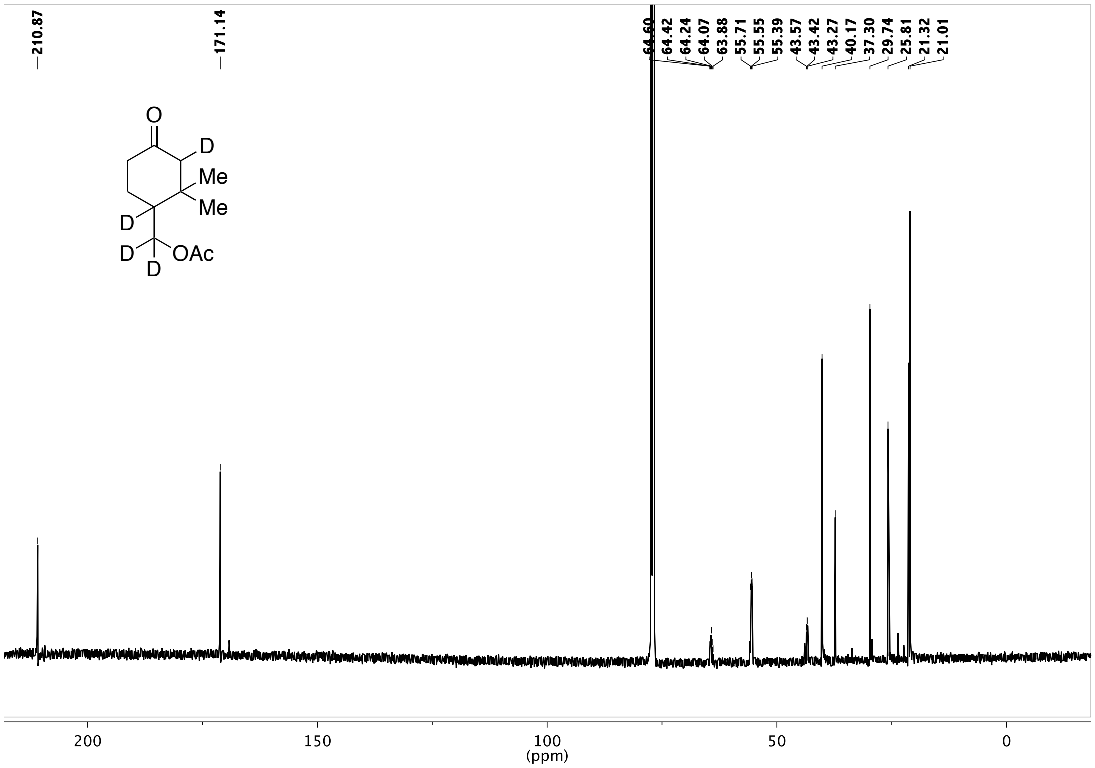

[(5*E*)-2,2-Dimethyl-4-oxo-5-(phenylmethylidene) (1,3,3-d3)cyclohexyl](*d*2)methyl 4-methylbenzene-1-sulfonate (20):

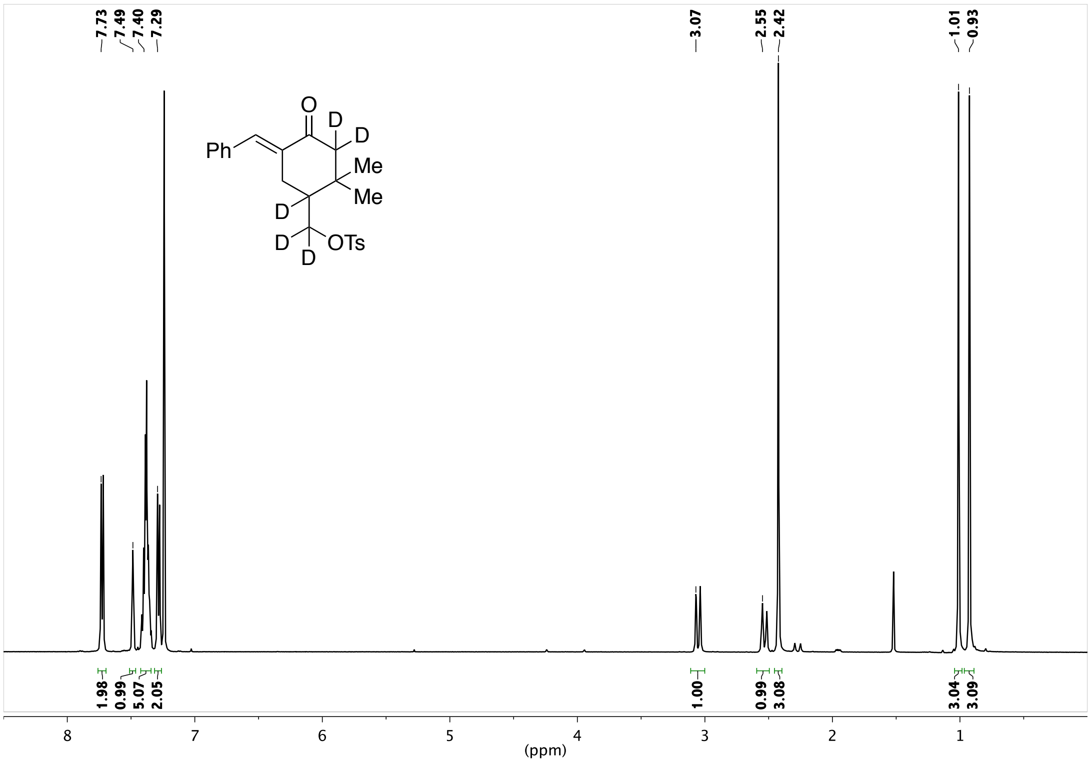

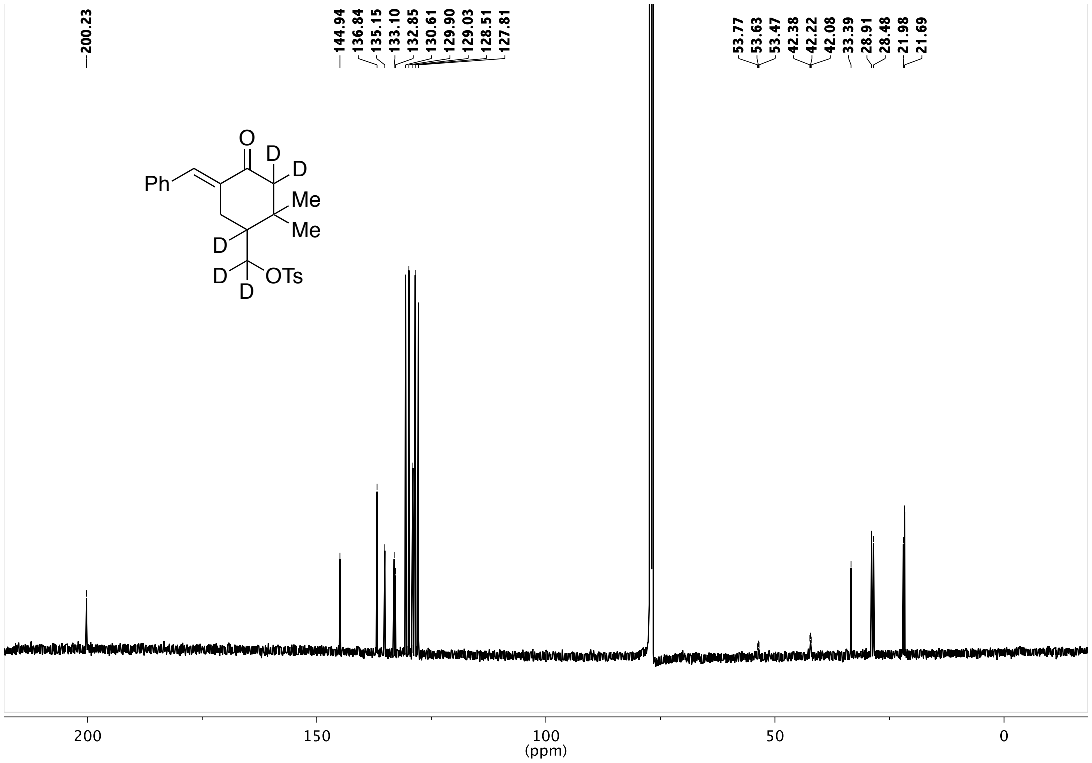

(3*E*)-6,6-Dimethyl-3-(phenylmethylidene) (1,5,7,7-*d*4)bicyclo[3.1.1]heptan-2-one (21):

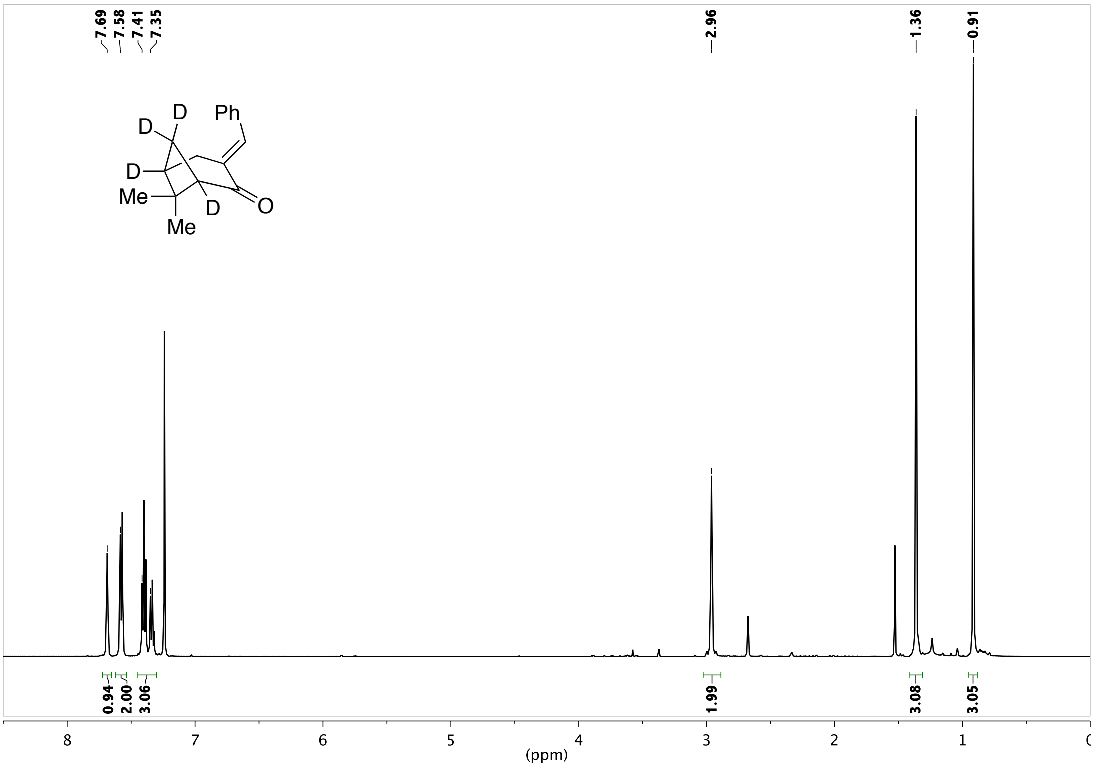

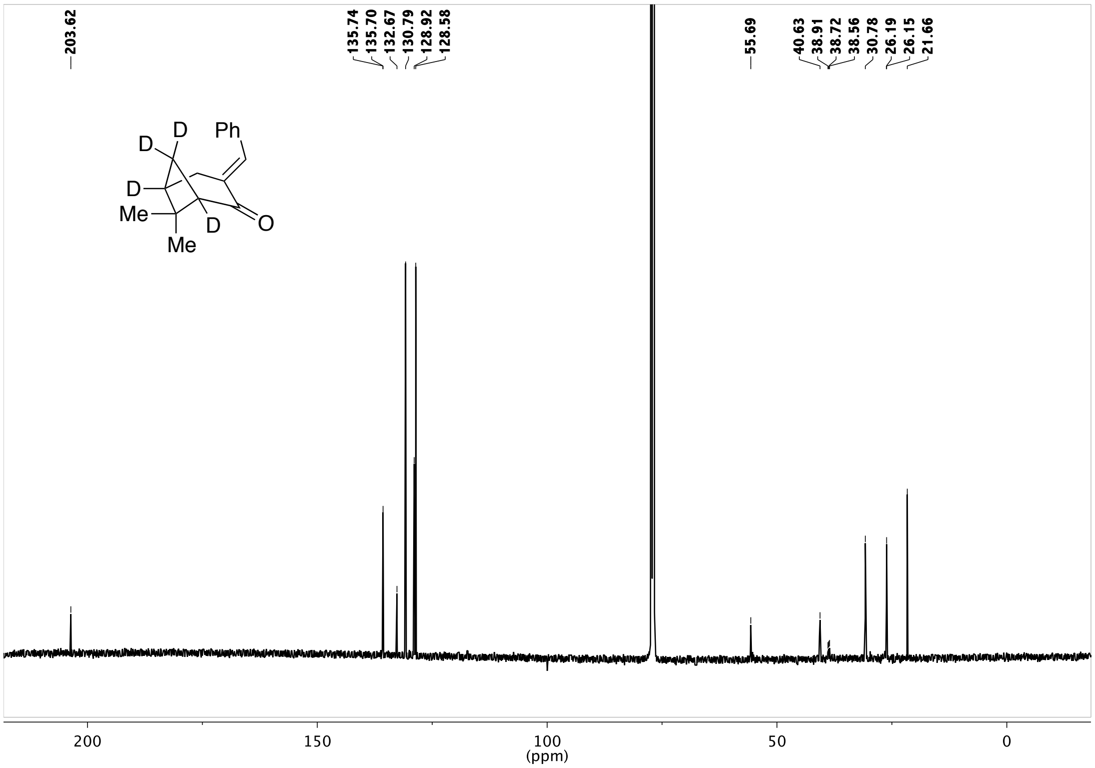

6,6-Dimethyl(1,3,3,4,5,7,7-*d*6)bicyclo[3.1.1]heptan-2-one (nopinone-*d*8) (22):

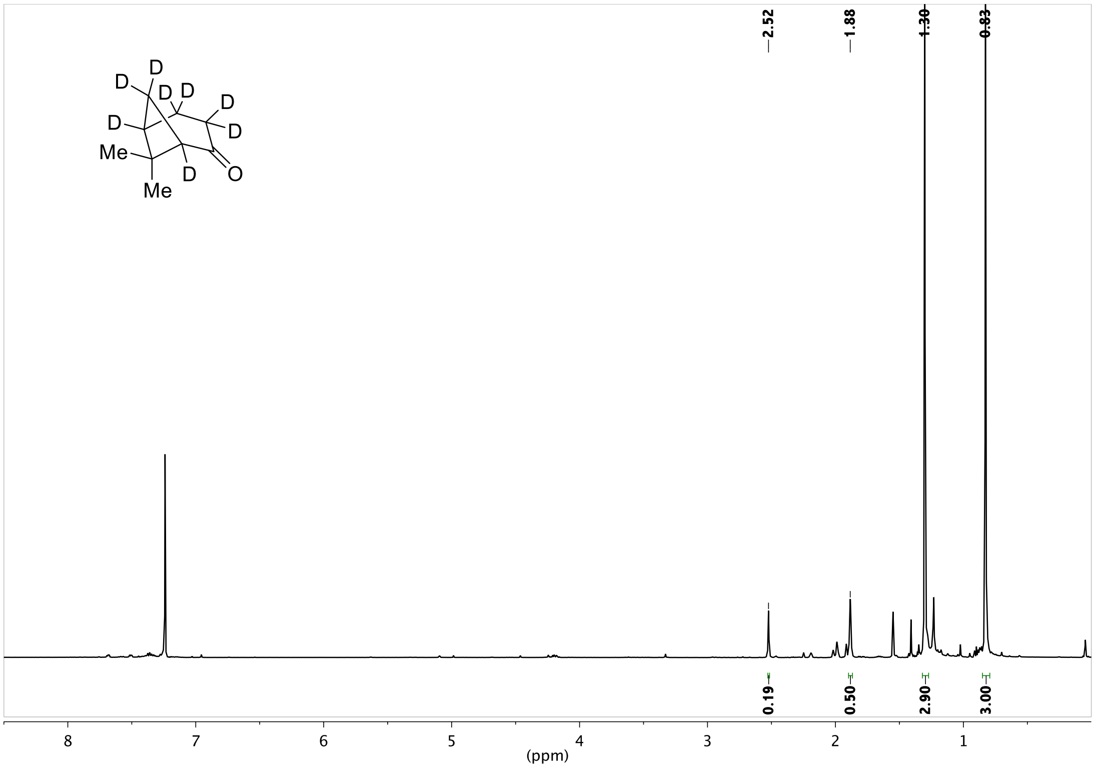

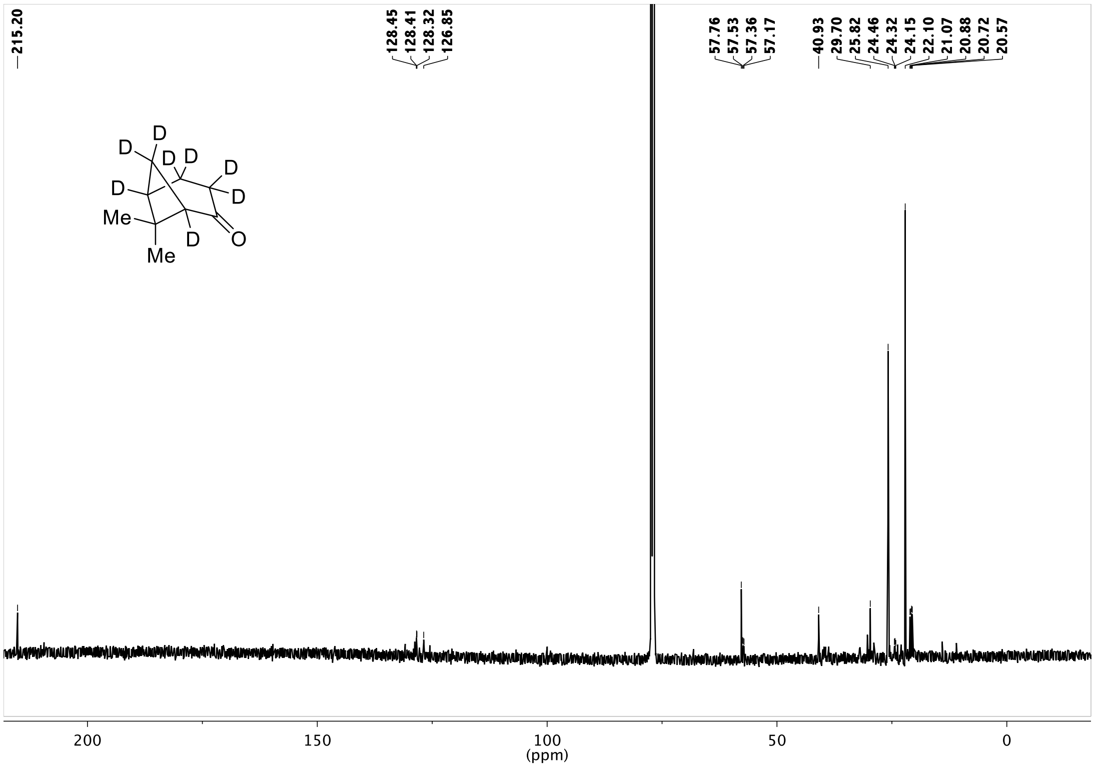

2,6,6-Trimethyl(1,3,4,5,7,7-*d*6)bicyclo[3.1.1]hept-2-ene ((±)-α-pinene-*d*7) (7):

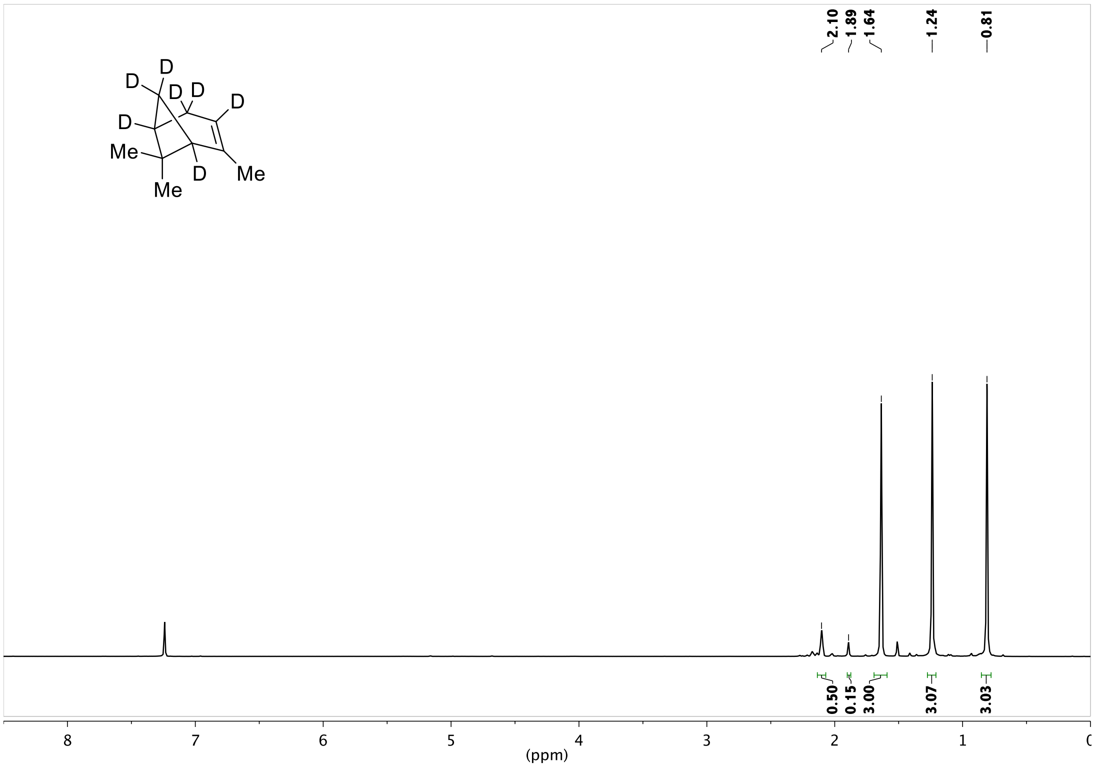

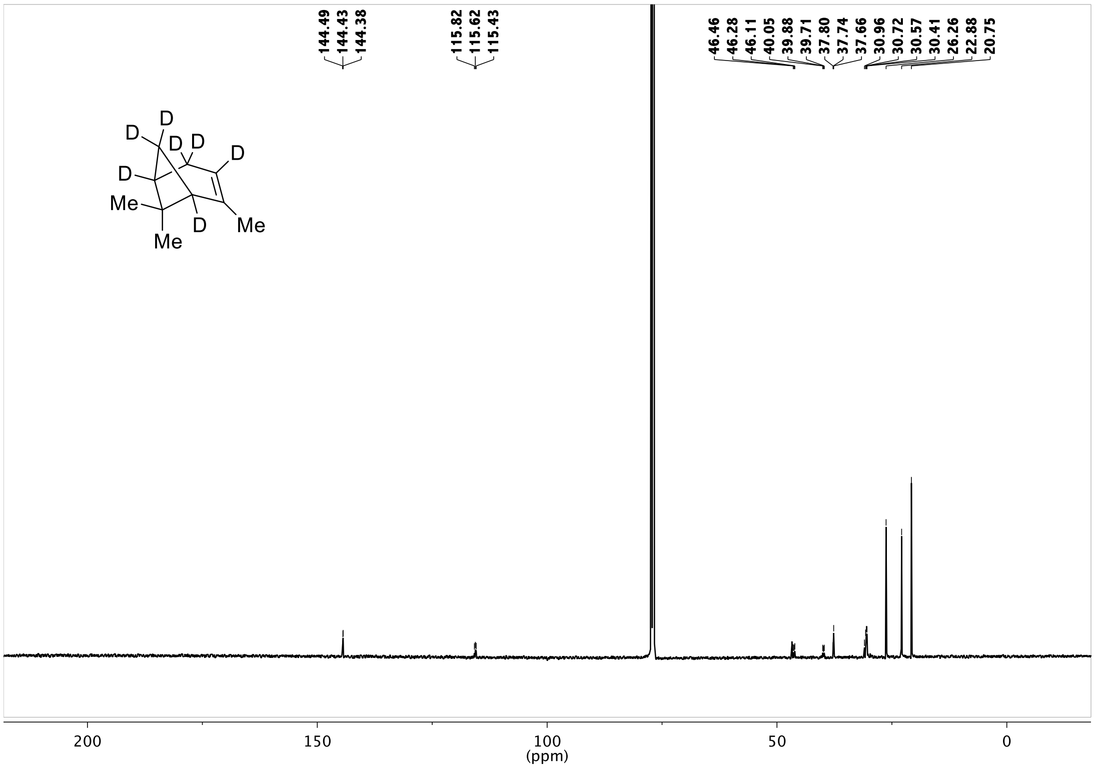

4. IR and Raman Spectra

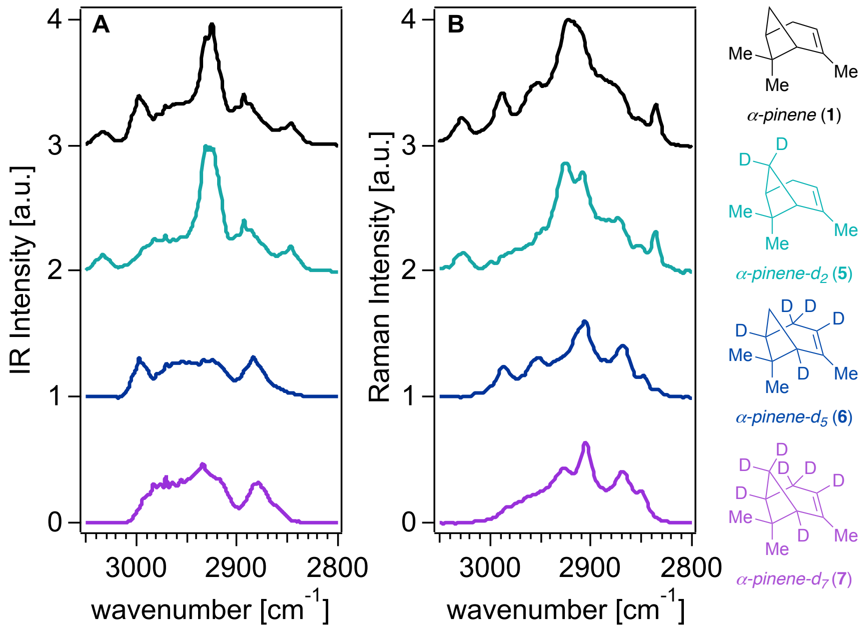

Figure S1. A) IR and B) Raman spectra of synthesized isotopologues **5**–**7**.

5. High Resolution Vapor-Phase SFG spectra on CaF_2

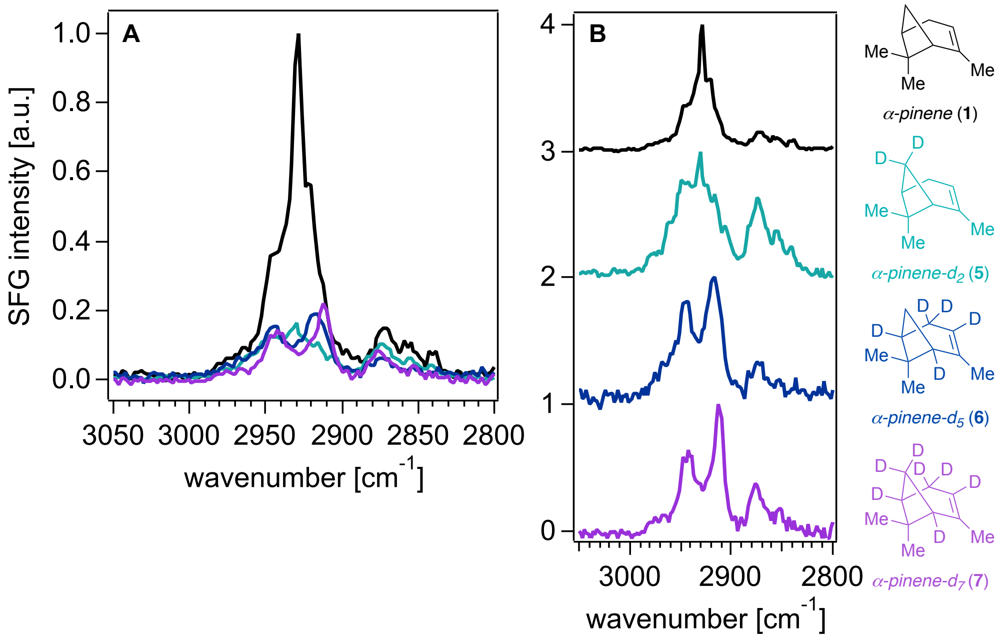

Figure S2. High resolution *ssp*-polarized vapor phase SFG spectra of (–)-α-pinene and α-pinene isotopologues **5**–**7** in contact with CaF_2. A) Intensities are scaled based the highest intensity peak of the vapor (−)-α-pinene SFG spectrum collected on the same day as each of the isotopologues. B) All maximum intensities have been normalized to 1 and offset for clarity.

6. Standard Resolution Vapor-Phase SFG spectra on fused silica

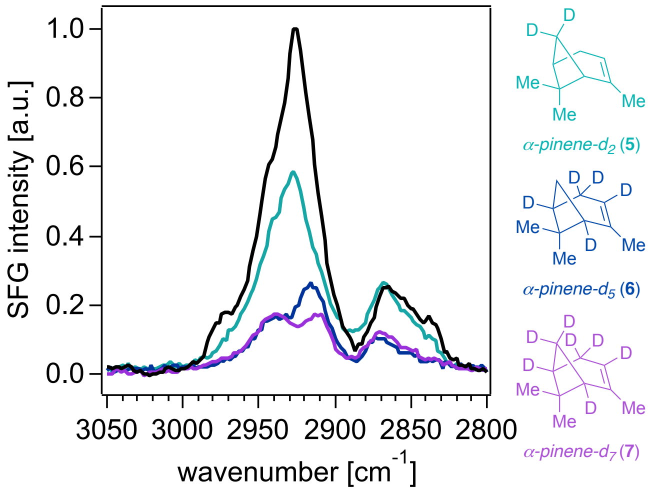

Figure S3. Standard resolution *ssp*-polarized vapor-phase SFG spectra of (–)-α-pinene and α-pinene isotopologues **5**–**7** in contact with fused silica. Intensities are scaled based the highest intensity peak of the vapor (−)-α-pinene SFG spectrum collected on the same day as each of the isotopologues.

7. Details on SOM collection. The experimental and collection conditions for producing (–)-α-pinene and deuterium substituted α-pinene SOM.

VOC Precursor	Conc. (ppb)	Mass loading (μg m^{-3})	Collection time (hr)	Mode diameter (nm)	Number concentration (# cm^{-3})	Mass collected (mg)
(–)-α-pinene (**1**)	125	62 ± 2	10	24 ± 4	$(6.7 \pm 0.3) \times 10^6$	0.1
(–)-α-pinene (**1**)	300	$(1.0 \pm 0.3) \times 10^3$	10	60 ± 5	$(2.8 \pm 0.4) \times 10^6$	1.0
α-pinene-d_3 (**2**)	125	97 ± 4	11	25± 4	$(7.0 \pm 0.6) \times 10^6$	0.1
α-pinene-vinyl-d_3 (**3**)	125	145 ± 7	6.5	26 ± 3	$(7.6 \pm 0.2) \times 10^6$	0.2
α-pinene-d_6 (**4**)	125	142 ± 7	8.5	25 ± 5	$(8.2 \pm 0.3) \times 10^6$	0.1
α-pinene-d_2 (**5**)	300	$(1.1 \pm 0.3) \times 10^3$	9	62 ± 4	$(2.9 \pm 0.3) \times 10^6$	1.0
α-pinene-d_5 (**6**)	300	$(1.1 \pm 0.2) \times 10^3$	9	59 ± 4	$(2.7 \pm 0.5) \times 10^6$	1.1
α-pinene-d_7 (**7**)	300	$(1.4 \pm 0.4) \times 10^3$	9	115 ± 5	$(9.7 \pm 0.8) \times 10^5$	1.8

8. References

1. A. B. Pangborn, M. A. Giardello, R. H. Grubbs, R. K. Rosen and F. J. Timmers, *Organometallics*, 1996, **15**, 1518-1520.
2. W. L. F. Armarego and C. L. L. Chai, *Purification of Laboratory Chemicals*, Butterworth-Heinemann, Oxford, 5th Edition edn., 2003.
3. M. T. Thomas and A. G. Fallis, *J. Am. Chem. Soc.*, 1976, **98**, 1227-1231.
4. M. T. Thomas and A. G. Fallis, *Tetrahedron Lett.*, 1973, **14**, 4687-4690.
